# Supplementary figures and images for: PIMT is a novel and potent suppressor of endothelial activation (part 1 of 2)
Source: eLife. 2023 Apr 18;12:e85754. doi: 10.7554/eLife.85754 (PMC10112892; doi:10.7554/eLife.85754)

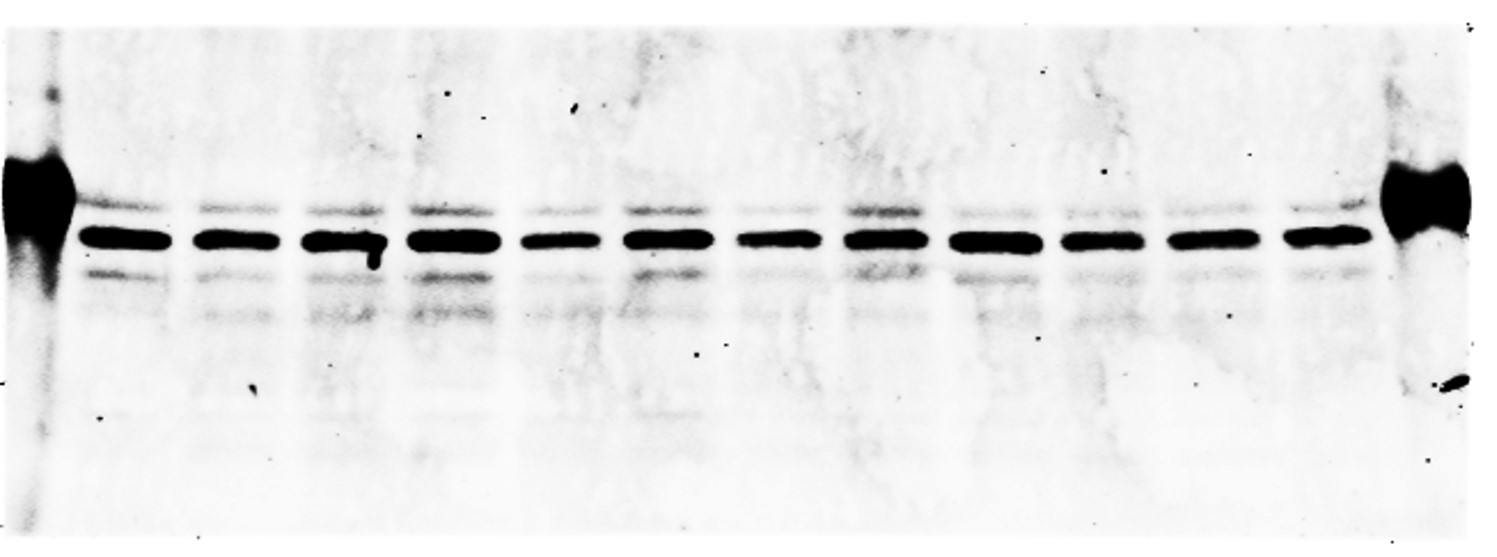

Supplement: Figure 1—source data 1. [file elife-85754-fig1-data1.zip › Figure 1-source data 1/Fig 1E GAPDH.jpg]

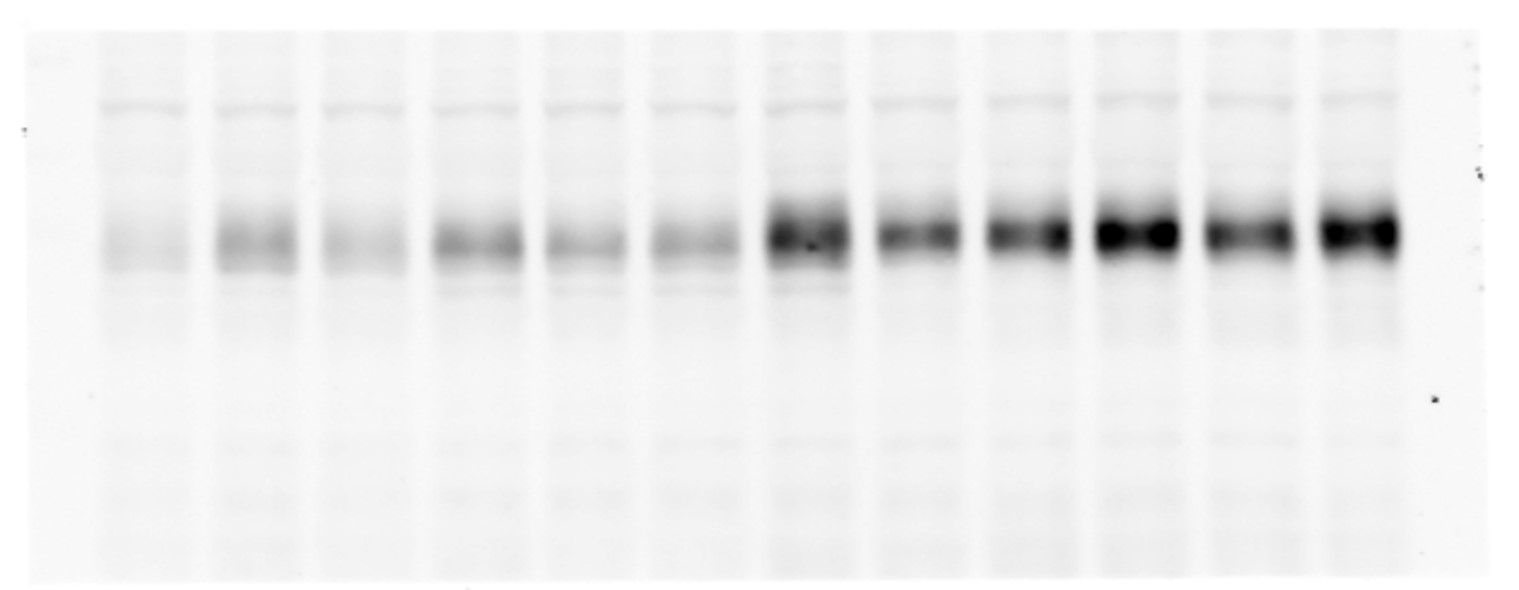

Supplement: Figure 1—source data 1. [file elife-85754-fig1-data1.zip › Figure 1-source data 1/Fig 1E ICAM1.jpg]

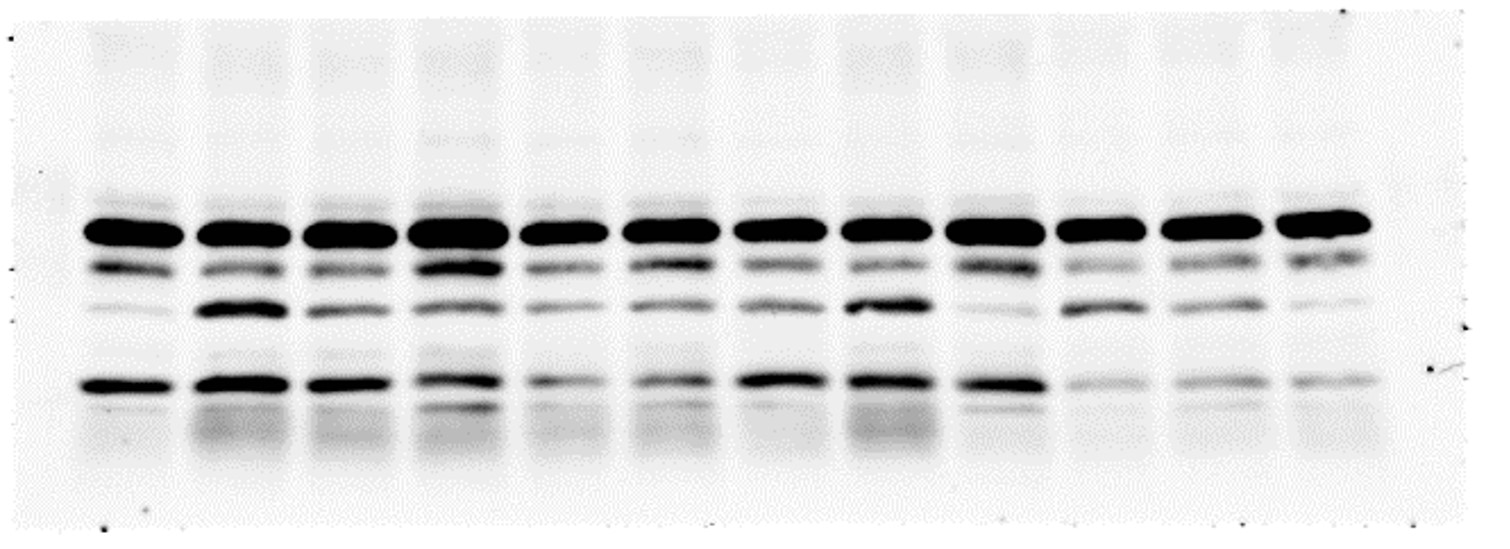

Supplement: Figure 1—source data 1. [file elife-85754-fig1-data1.zip › Figure 1-source data 1/Fig 1E PIMT.jpg]

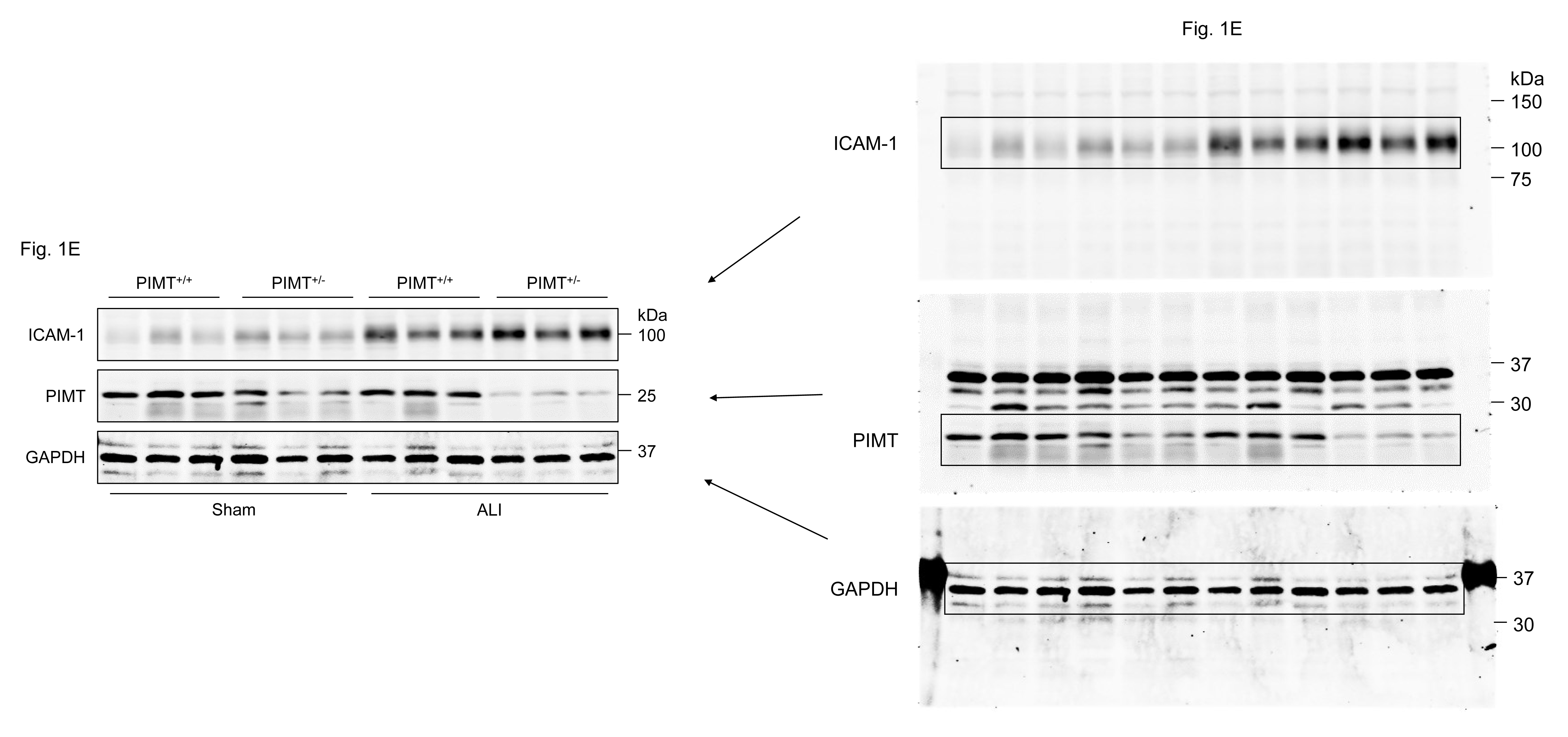

Supplement: Figure 1—source data 1. [file elife-85754-fig1-data1.zip › Figure 1-source data 1/Figure 1E.tif]

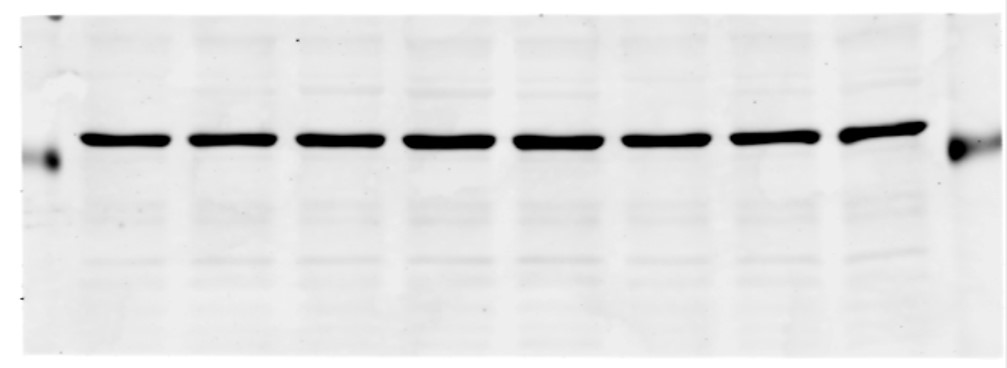

Supplement: Figure 2—source data 1. [file elife-85754-fig2-data1.zip › Figure 2-source data 1/Fig 2A GAPDH.jpg]

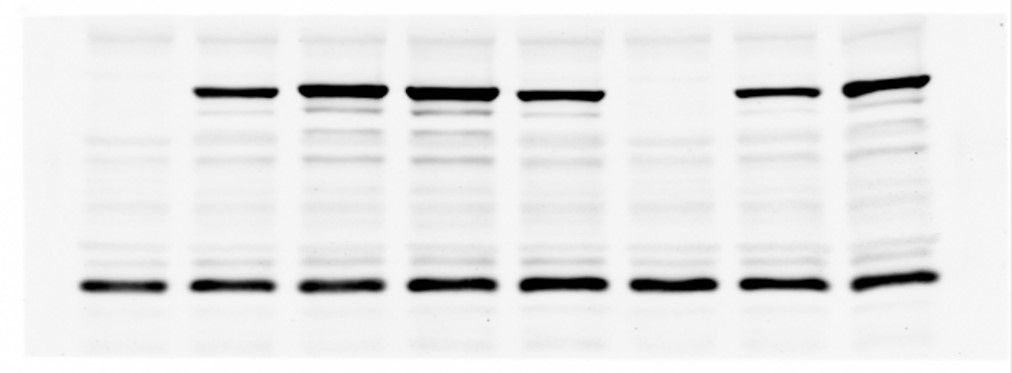

Supplement: Figure 2—source data 1. [file elife-85754-fig2-data1.zip › Figure 2-source data 1/Fig 2A PIMT.jpg]

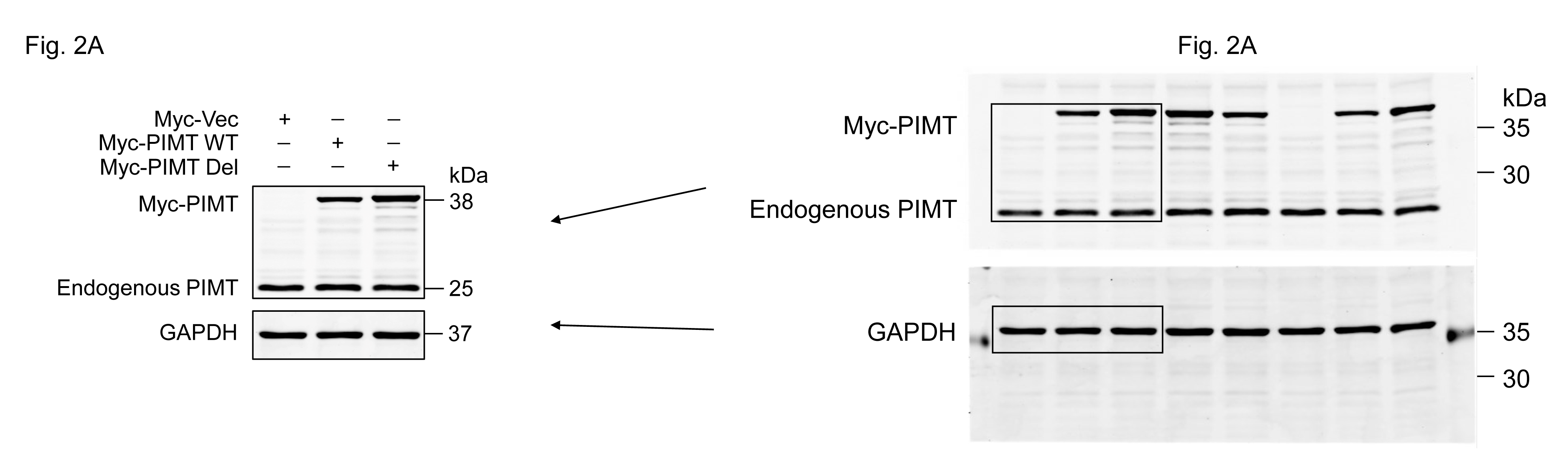

Supplement: Figure 2—source data 1. [file elife-85754-fig2-data1.zip › Figure 2-source data 1/Figure 2A.tif]

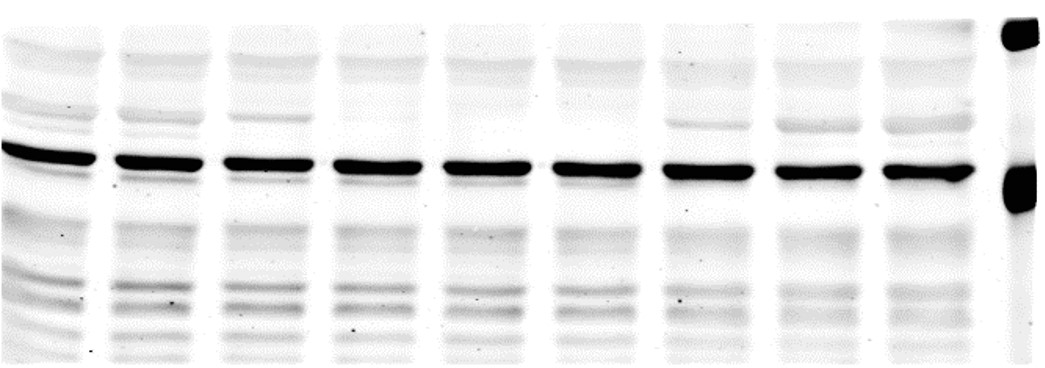

Supplement: Figure 2—source data 2. [file elife-85754-fig2-data2.zip › Figure 2- souce data 2/Fig 2B GAPDH.jpg]

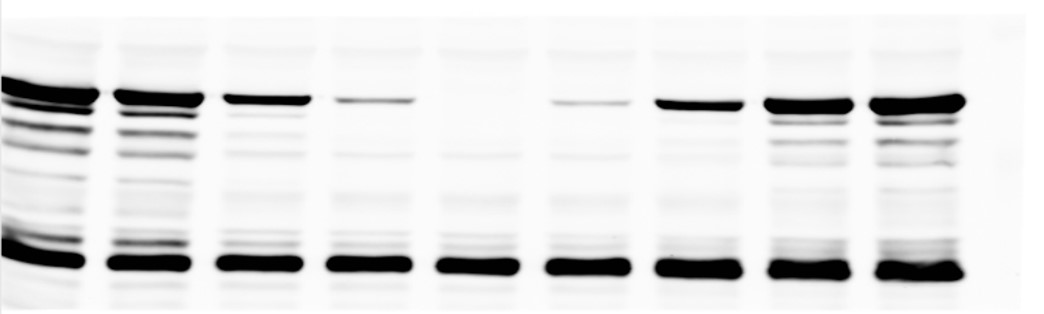

Supplement: Figure 2—source data 2. [file elife-85754-fig2-data2.zip › Figure 2- souce data 2/Fig 2B PIMT.jpg]

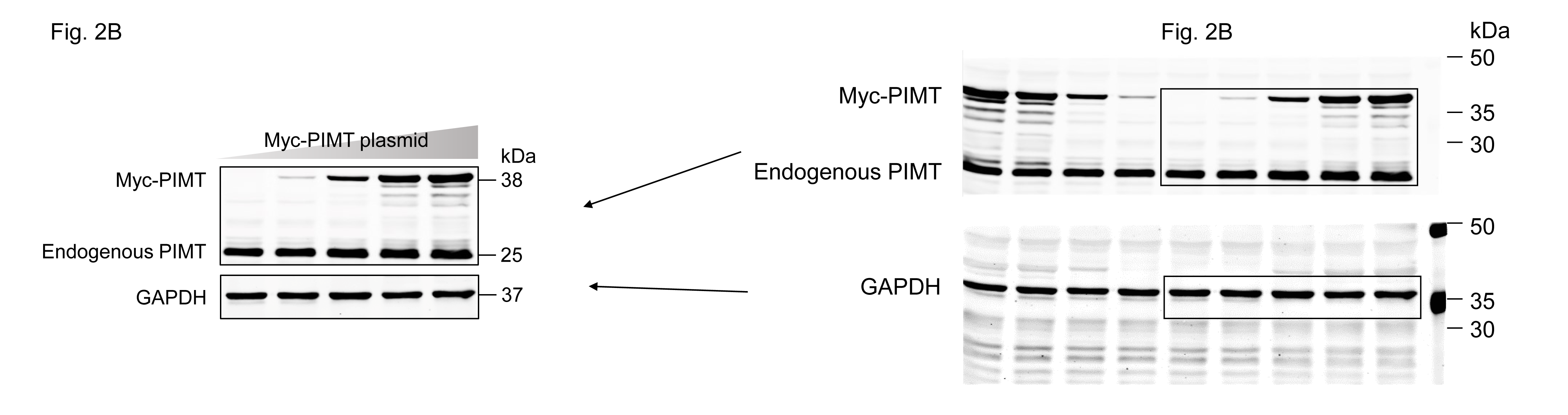

Supplement: Figure 2—source data 2. [file elife-85754-fig2-data2.zip › Figure 2- souce data 2/Figure 2B.tif]

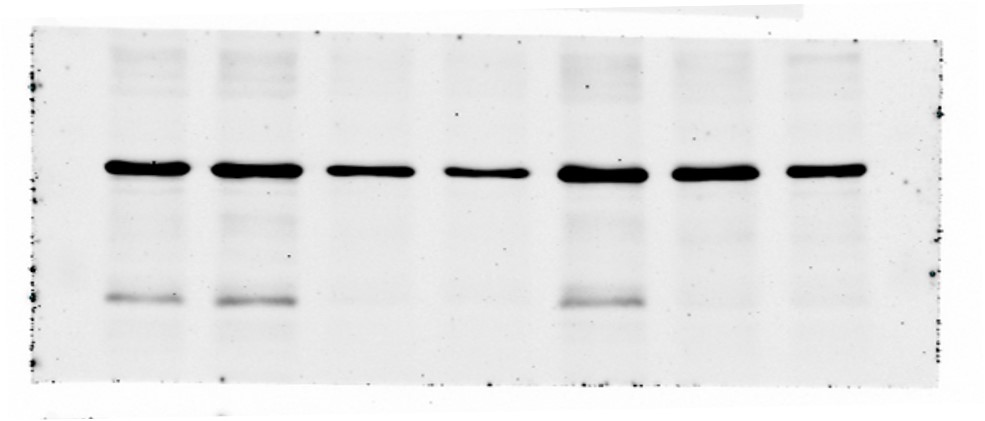

Supplement: Figure 2—source data 3. [file elife-85754-fig2-data3.zip › Figure 2- souce data 3/Fig 2C GAPDH.jpg]

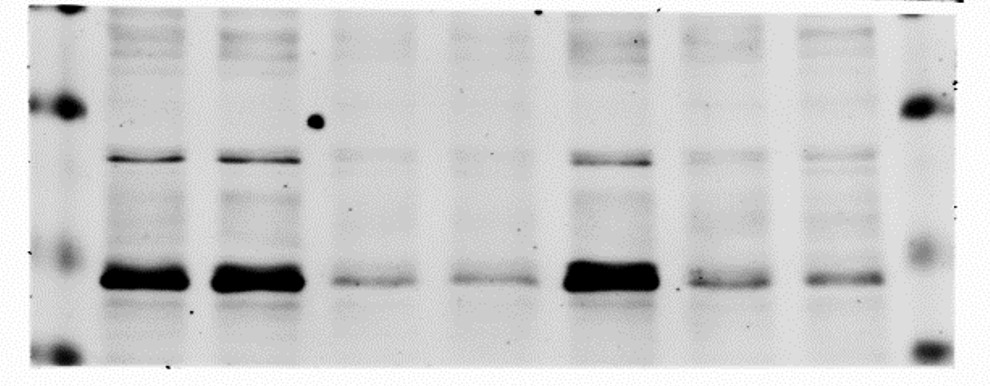

Supplement: Figure 2—source data 3. [file elife-85754-fig2-data3.zip › Figure 2- souce data 3/Fig 2C PIMT.jpg]

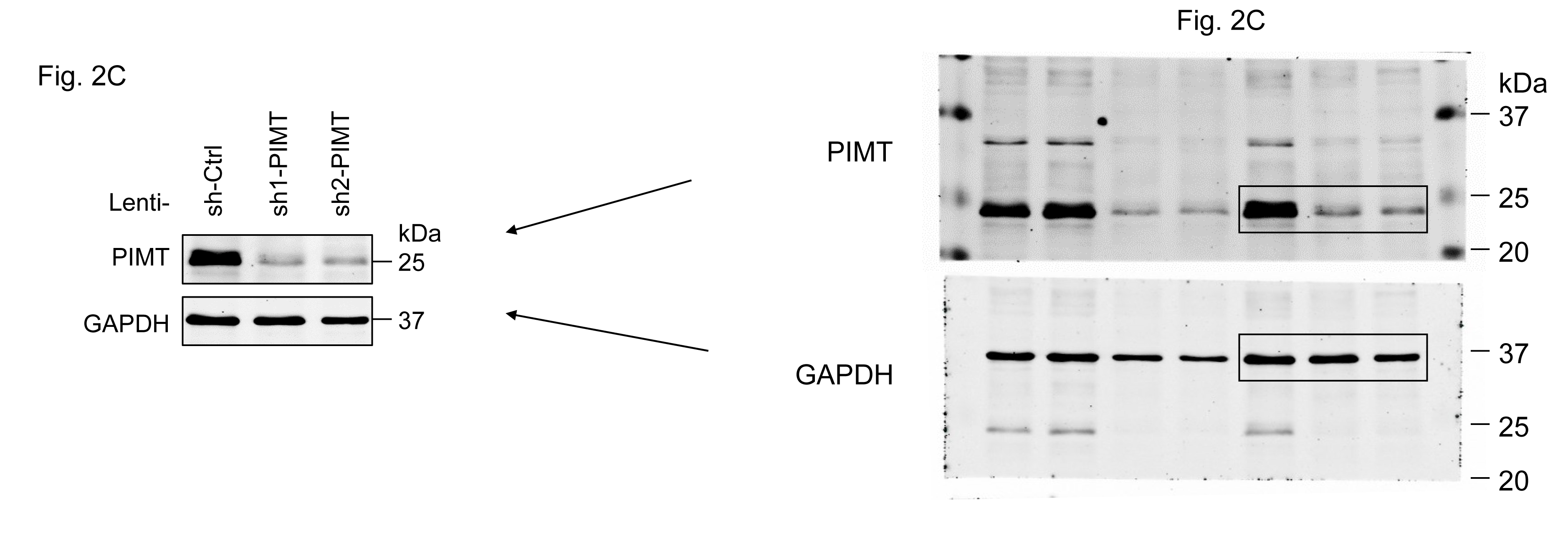

Supplement: Figure 2—source data 3. [file elife-85754-fig2-data3.zip › Figure 2- souce data 3/Figure 2C.tif]

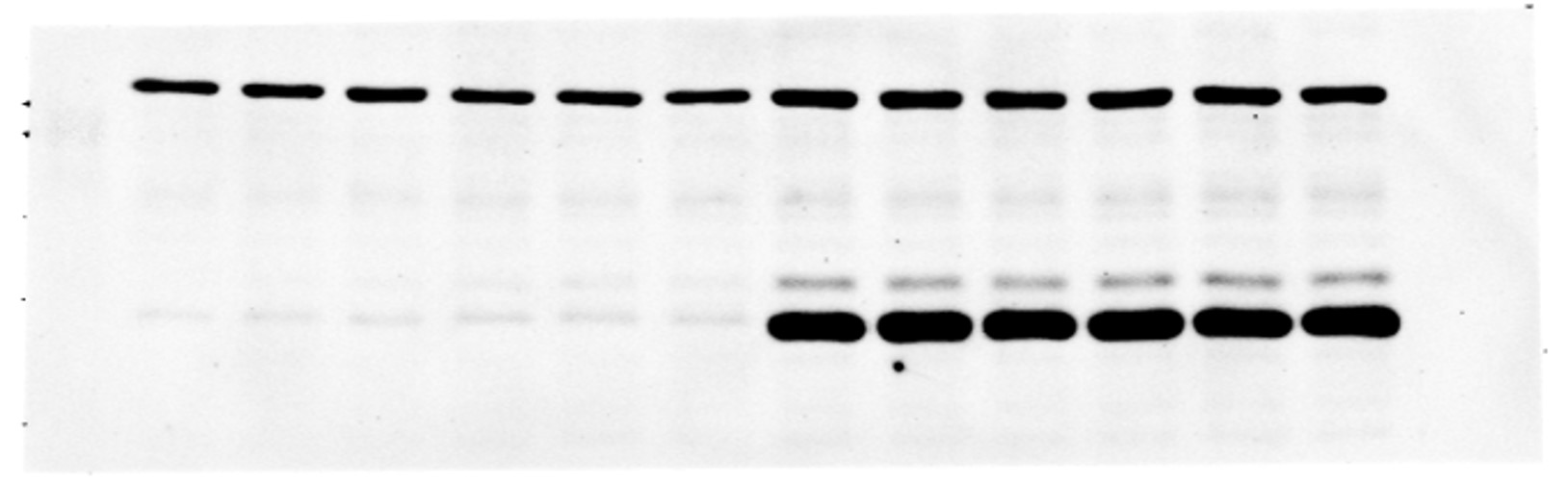

Supplement: Figure 2—source data 4. [file elife-85754-fig2-data4.zip › Figure 2- souce data 4/Fig 2E GAPDH.jpg]

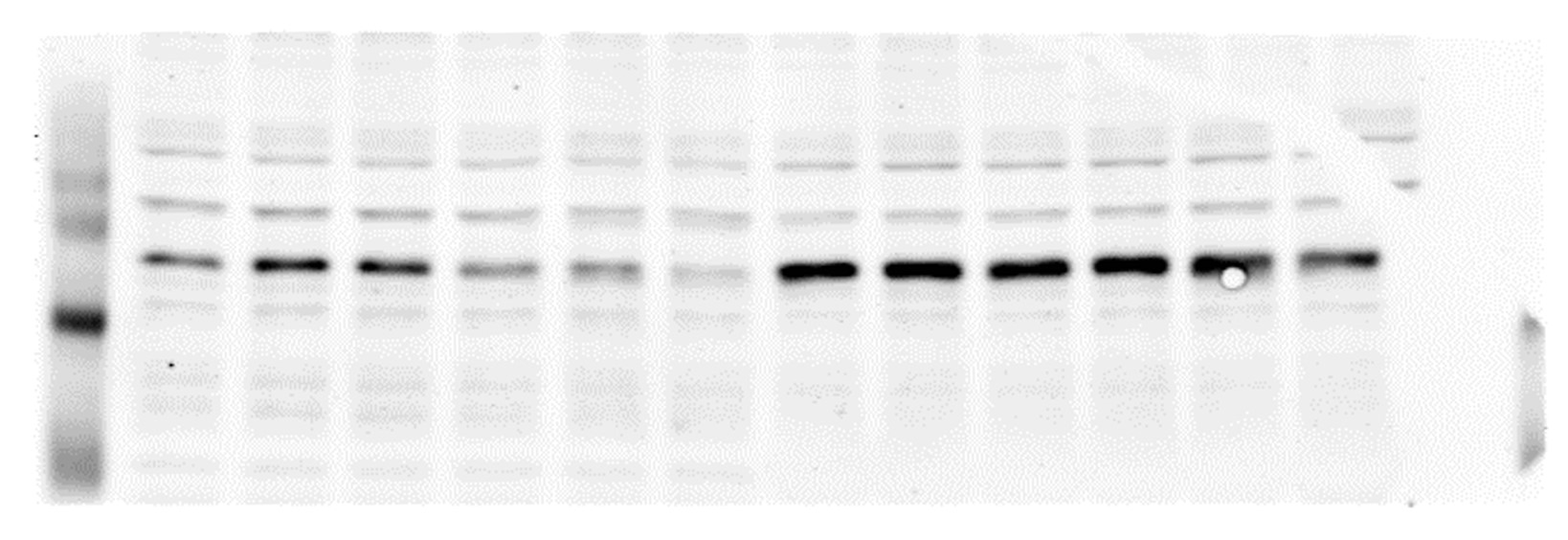

Supplement: Figure 2—source data 4. [file elife-85754-fig2-data4.zip › Figure 2- souce data 4/Fig 2E IkBa.jpg]

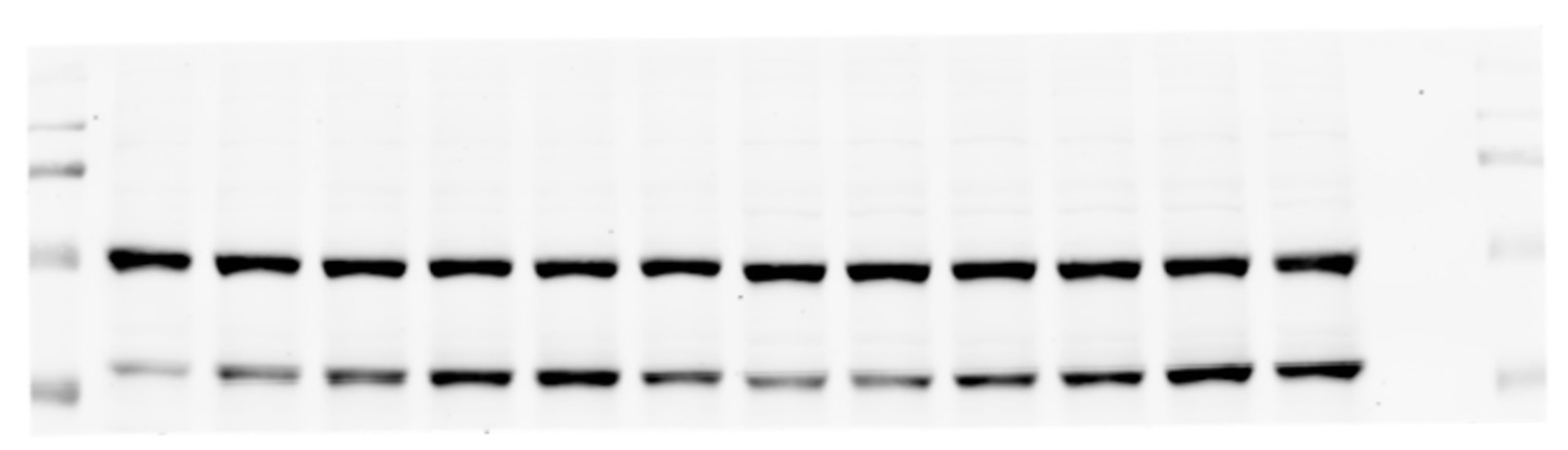

Supplement: Figure 2—source data 4. [file elife-85754-fig2-data4.zip › Figure 2- souce data 4/Fig 2E IKKb.jpg]

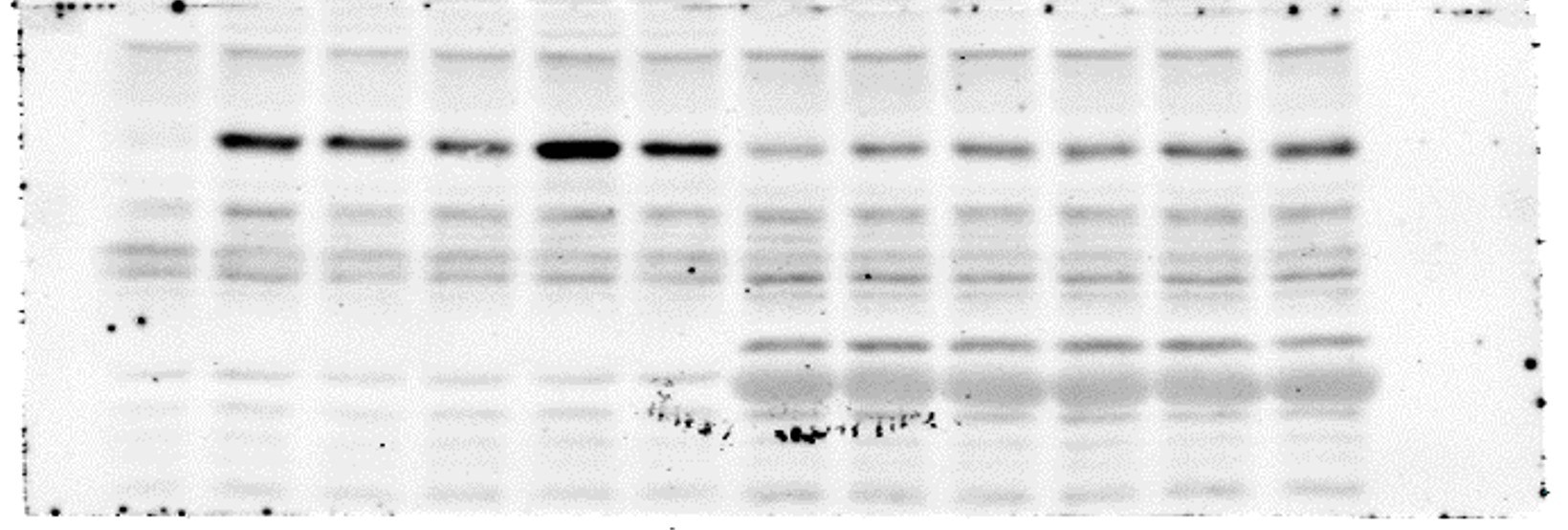

Supplement: Figure 2—source data 4. [file elife-85754-fig2-data4.zip › Figure 2- souce data 4/Fig 2E P-IkBa.jpg]

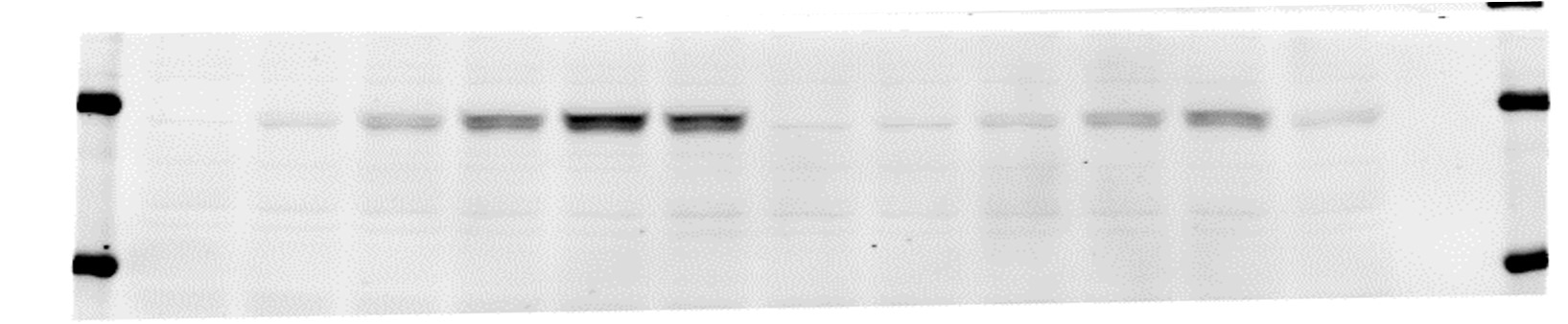

Supplement: Figure 2—source data 4. [file elife-85754-fig2-data4.zip › Figure 2- souce data 4/Fig 2E P-IKKb.jpg]

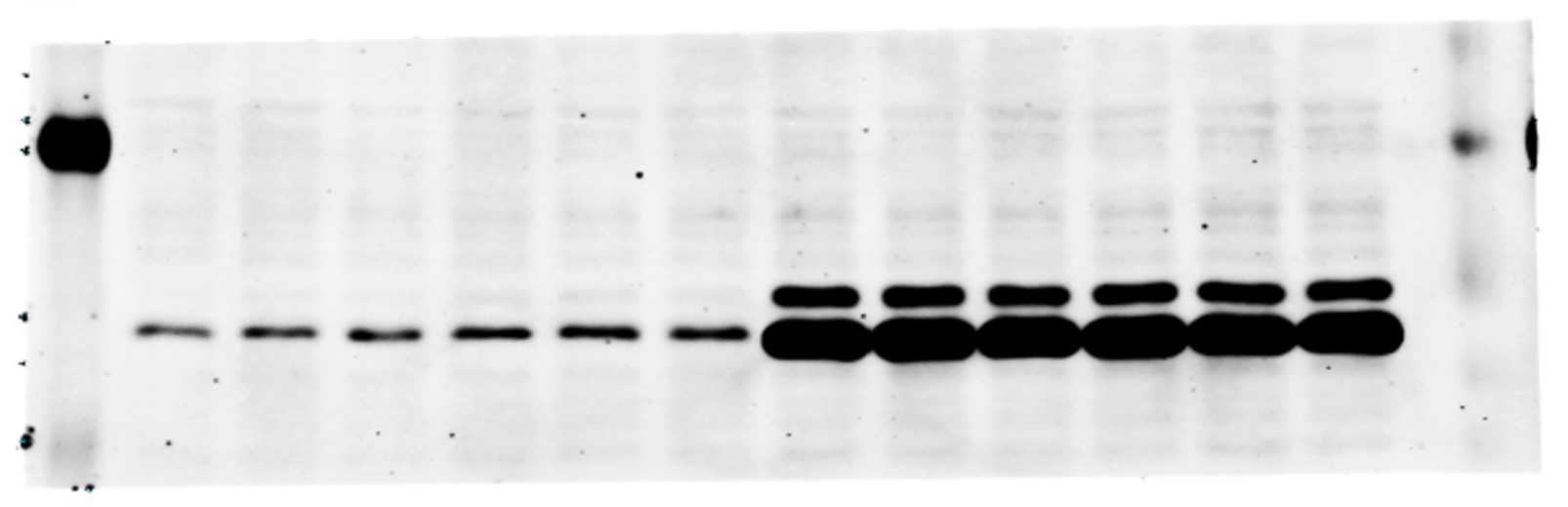

Supplement: Figure 2—source data 4. [file elife-85754-fig2-data4.zip › Figure 2- souce data 4/Fig 2E PIMT.jpg]

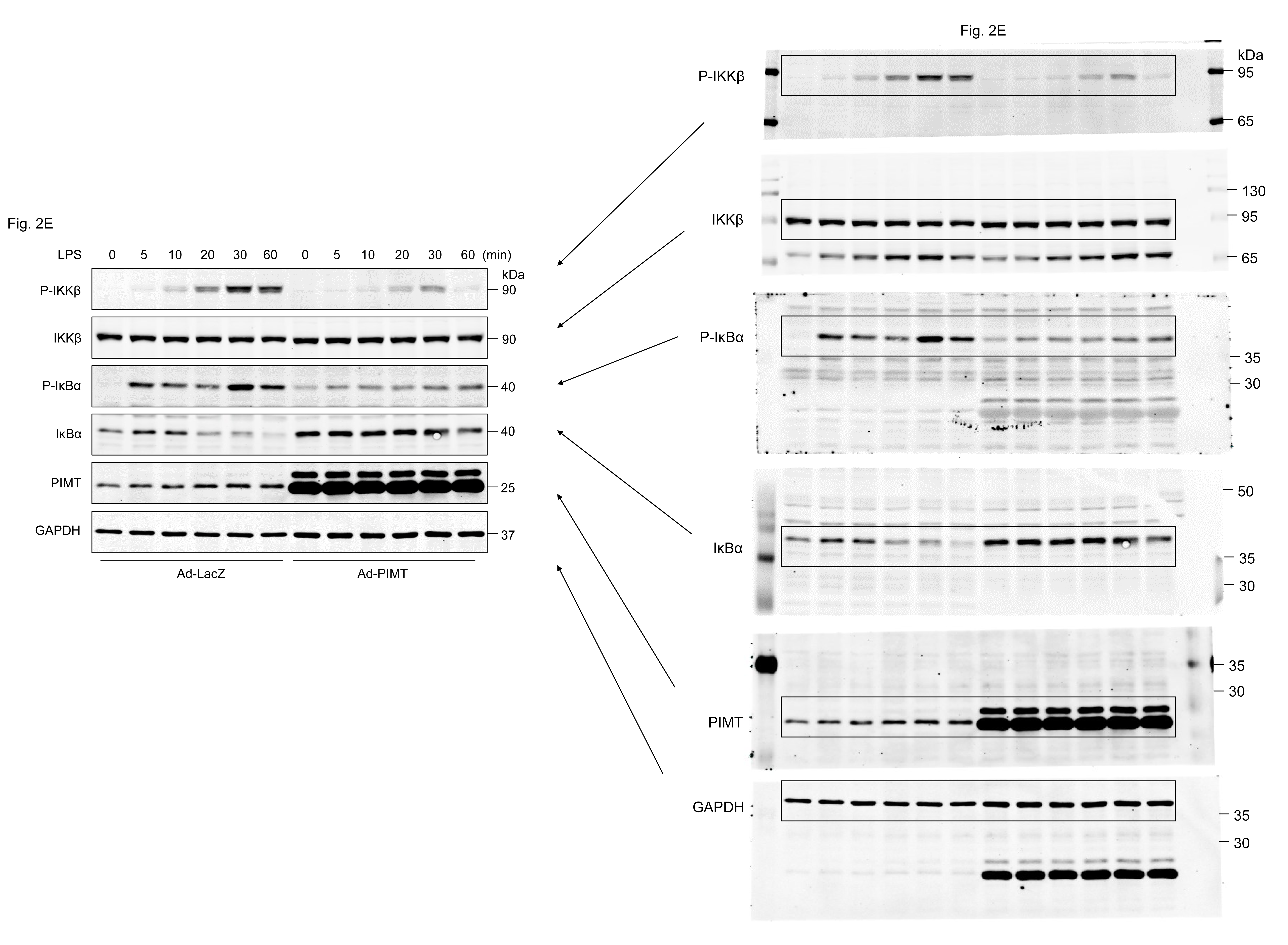

Supplement: Figure 2—source data 4. [file elife-85754-fig2-data4.zip › Figure 2- souce data 4/Figure 2E.tif]

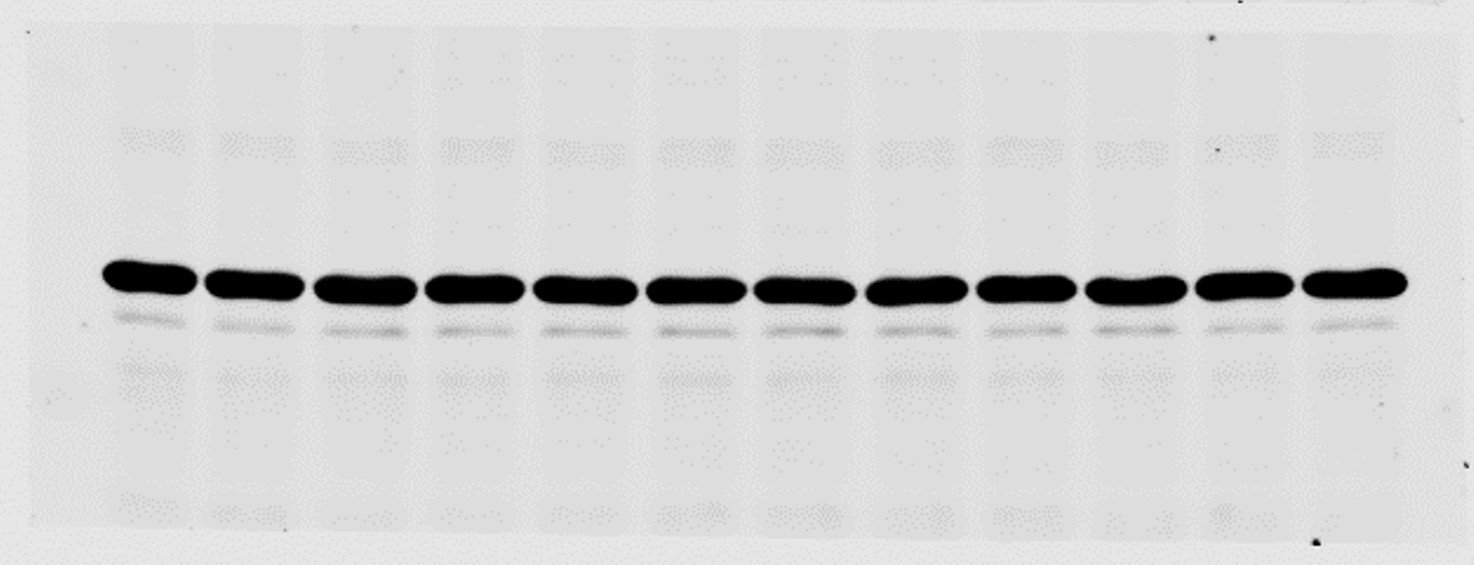

Supplement: Figure 2—source data 5. [file elife-85754-fig2-data5.zip › Figure 2- souce data 5/Fig 2F GAPDH.jpg]

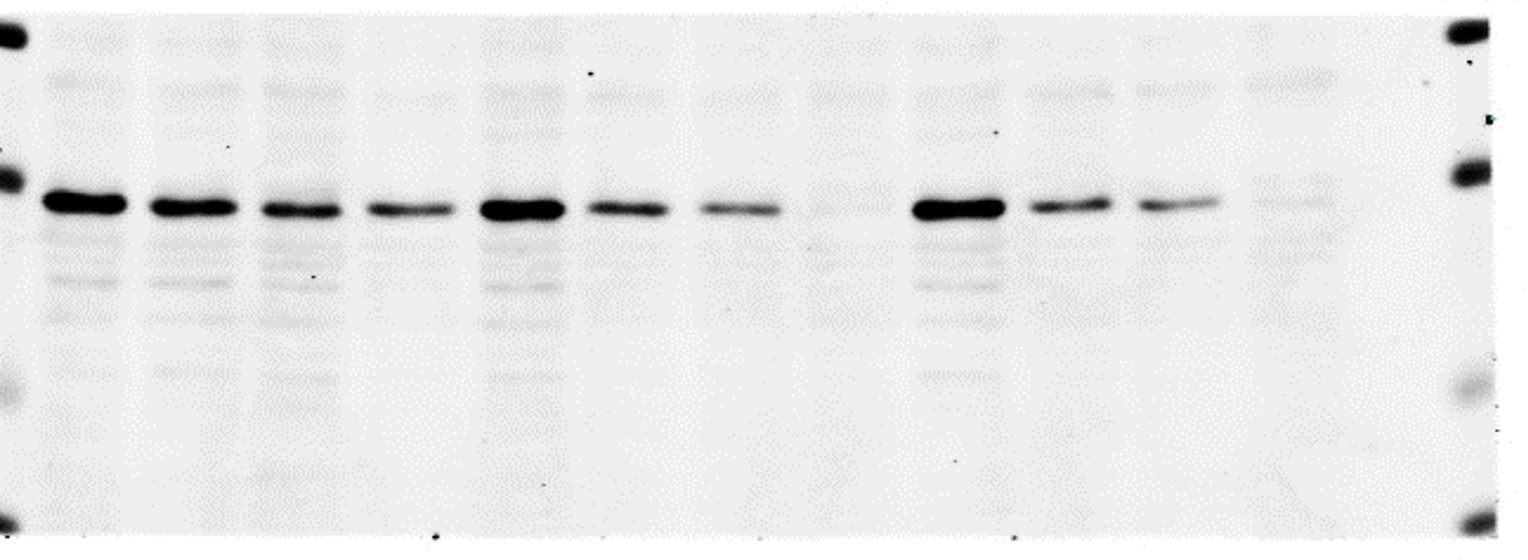

Supplement: Figure 2—source data 5. [file elife-85754-fig2-data5.zip › Figure 2- souce data 5/Fig 2F IkBa.jpg]

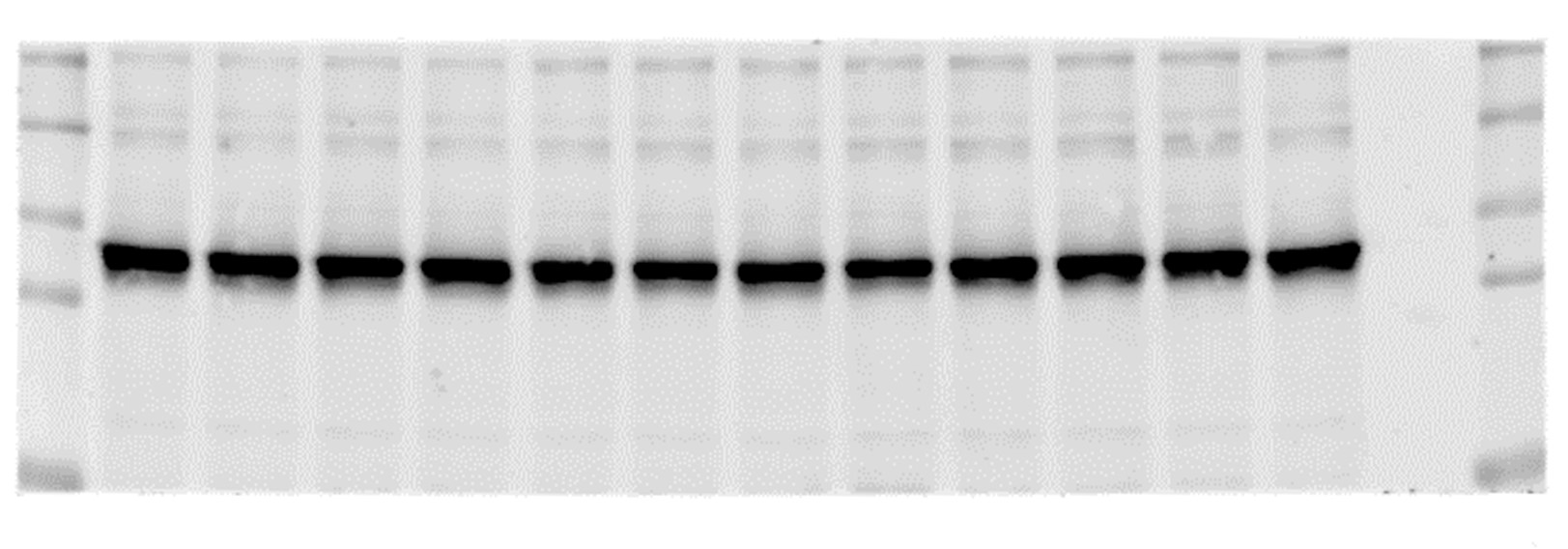

Supplement: Figure 2—source data 5. [file elife-85754-fig2-data5.zip › Figure 2- souce data 5/Fig 2F IKKb.jpg]

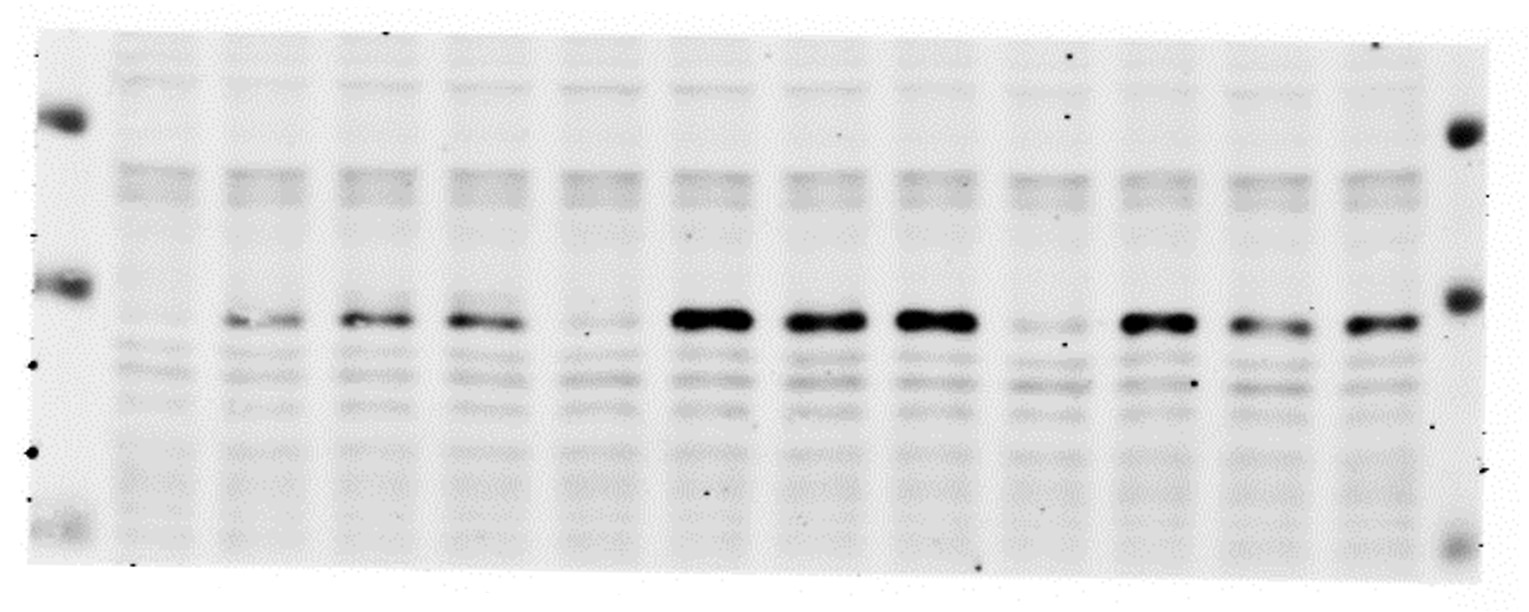

Supplement: Figure 2—source data 5. [file elife-85754-fig2-data5.zip › Figure 2- souce data 5/Fig 2F P-IkBa.jpg]

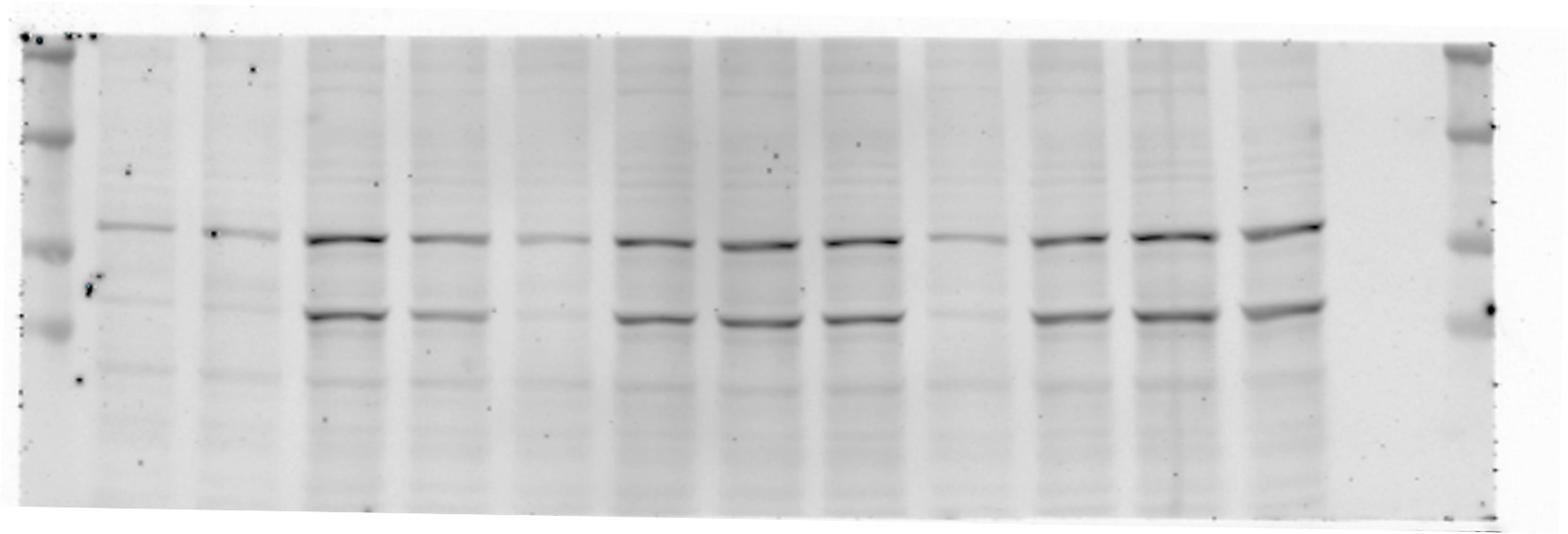

Supplement: Figure 2—source data 5. [file elife-85754-fig2-data5.zip › Figure 2- souce data 5/Fig 2F P-IKKb.jpg]

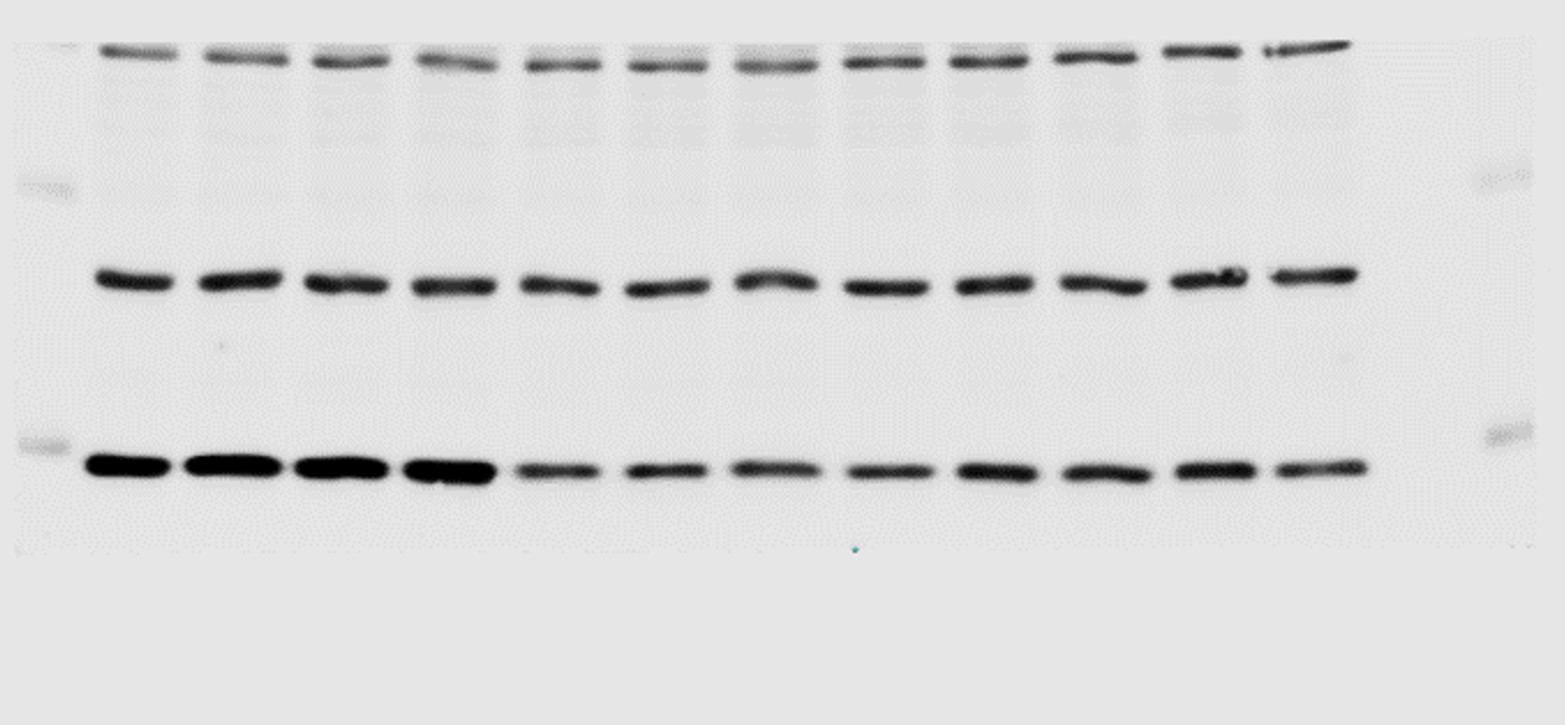

Supplement: Figure 2—source data 5. [file elife-85754-fig2-data5.zip › Figure 2- souce data 5/Fig 2F PIMT.jpg]

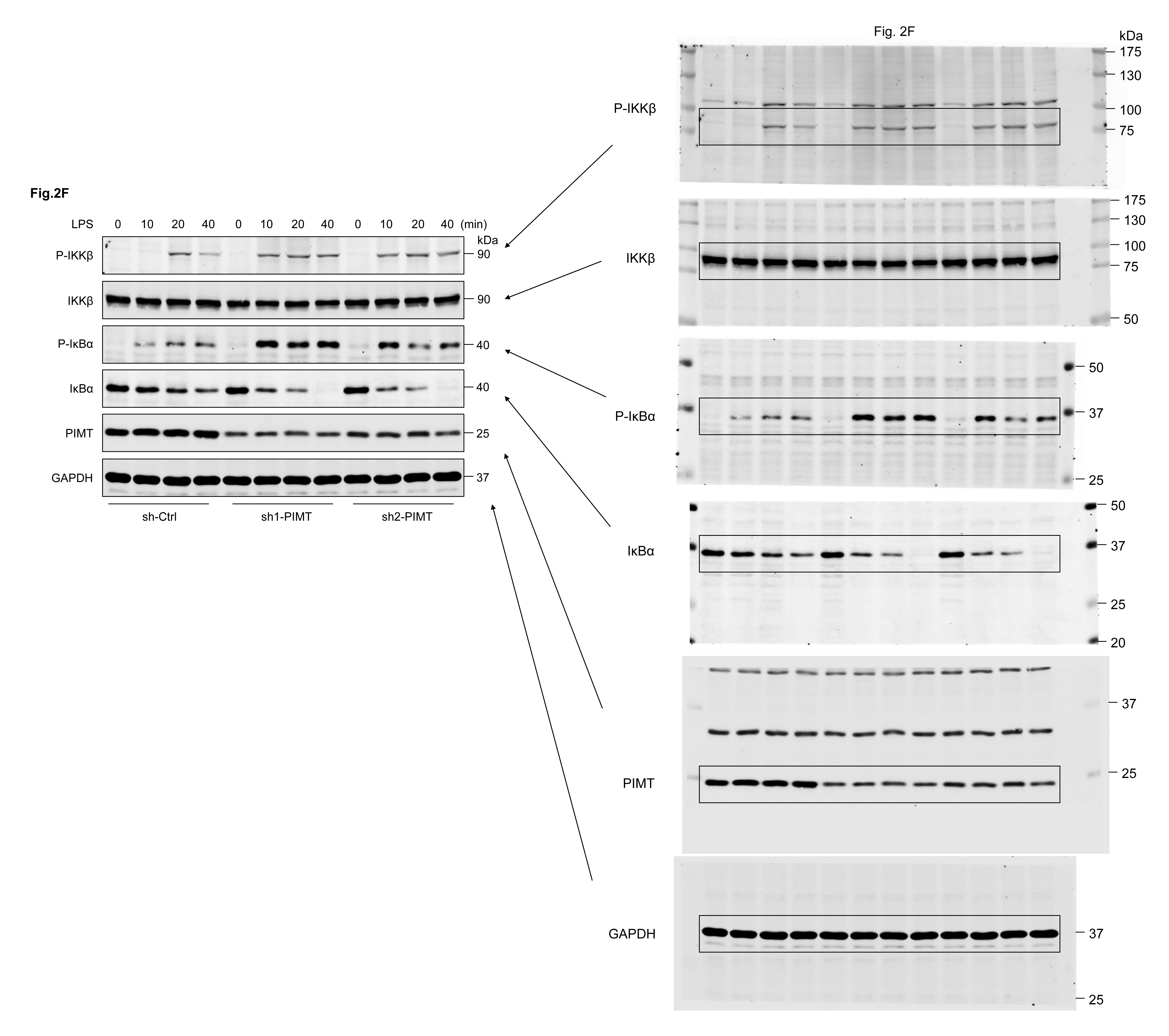

Supplement: Figure 2—source data 5. [file elife-85754-fig2-data5.zip › Figure 2- souce data 5/Figure 2F.tif]

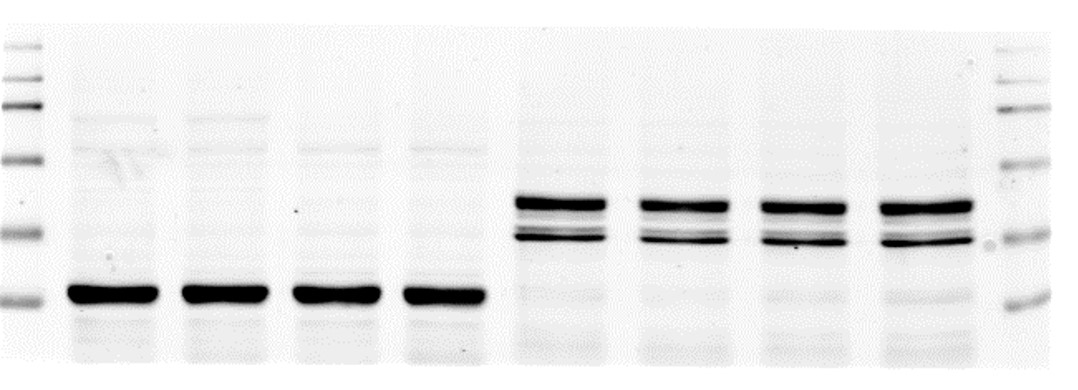

Supplement: Figure 2—source data 6. [file elife-85754-fig2-data6.zip › Figure 2- souce data 6/Fig 2G GAPDH.jpg]

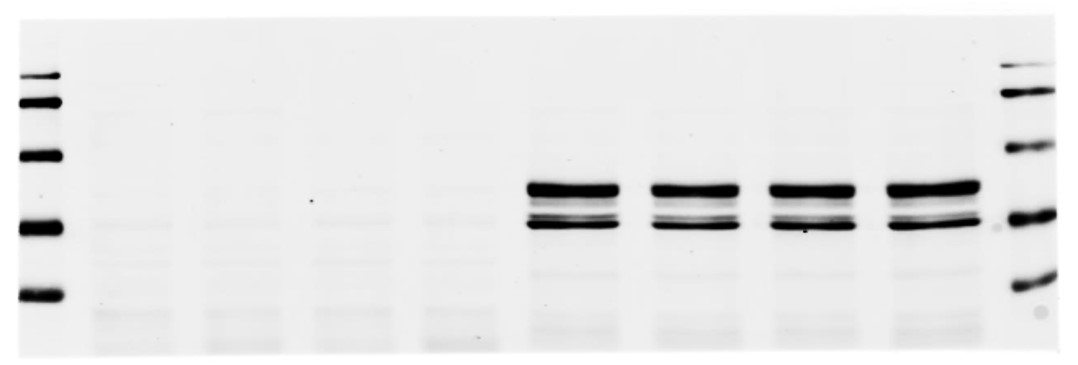

Supplement: Figure 2—source data 6. [file elife-85754-fig2-data6.zip › Figure 2- souce data 6/Fig 2G LaminAC.jpg]

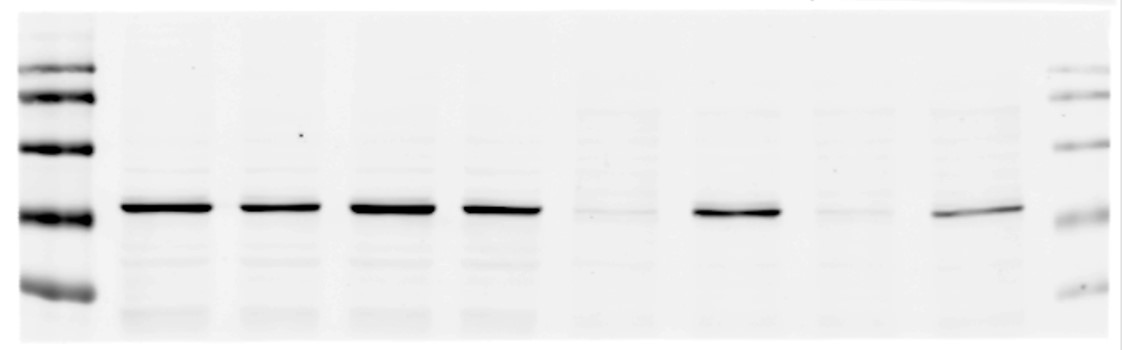

Supplement: Figure 2—source data 6. [file elife-85754-fig2-data6.zip › Figure 2- souce data 6/Fig 2G p65.jpg]

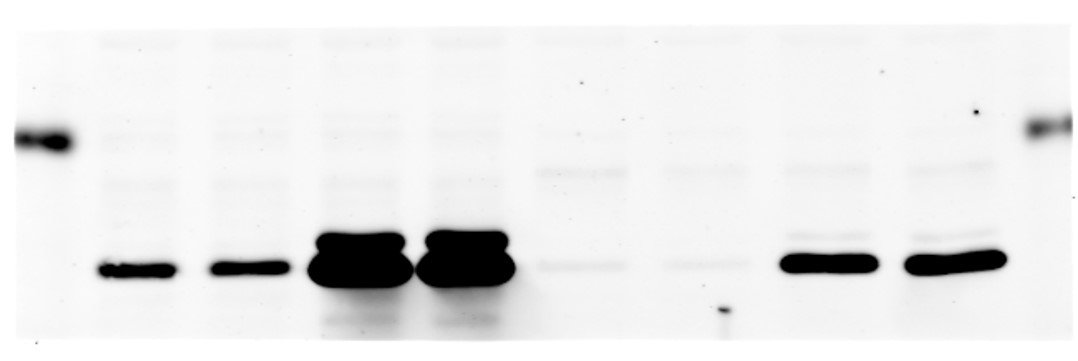

Supplement: Figure 2—source data 6. [file elife-85754-fig2-data6.zip › Figure 2- souce data 6/Fig 2G PIMT.jpg]

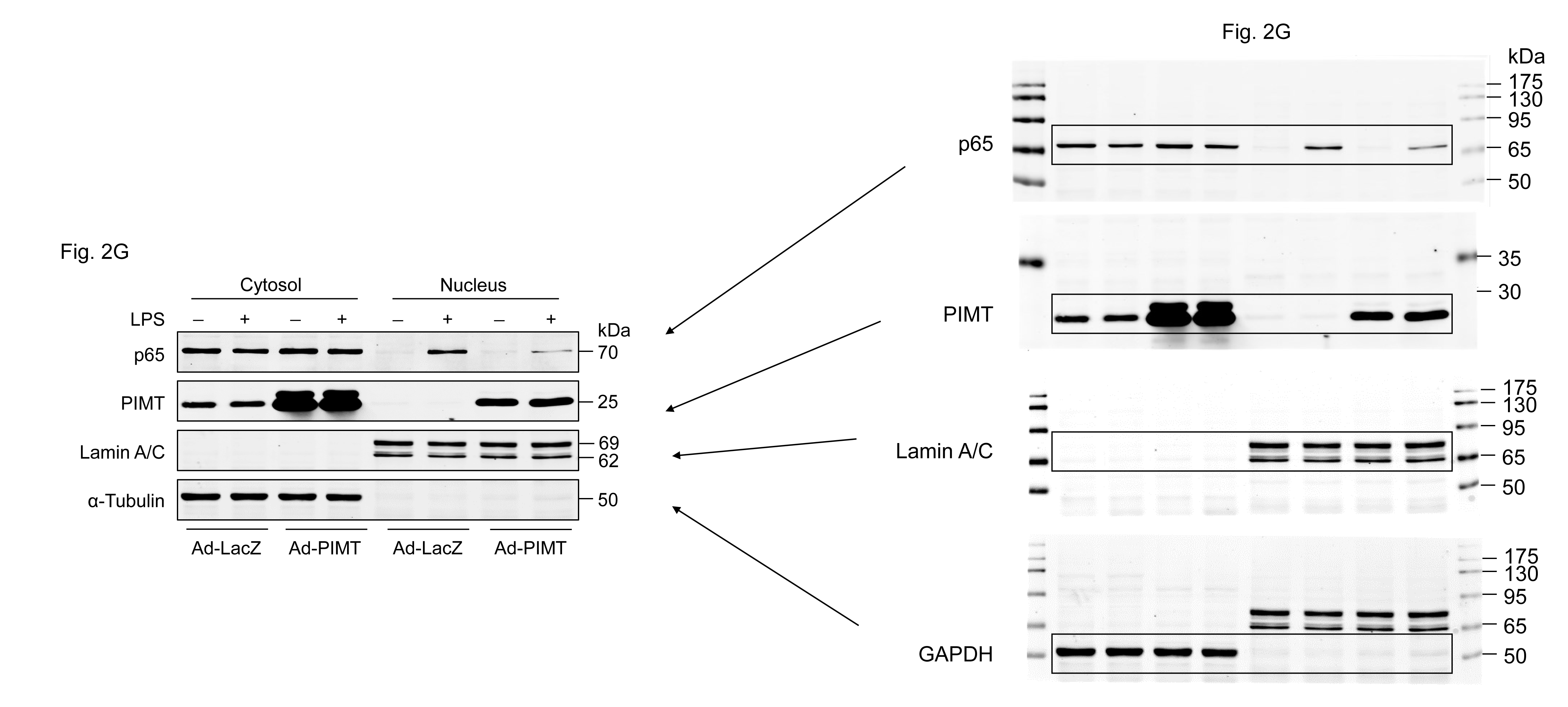

Supplement: Figure 2—source data 6. [file elife-85754-fig2-data6.zip › Figure 2- souce data 6/Figure 2G.tif]

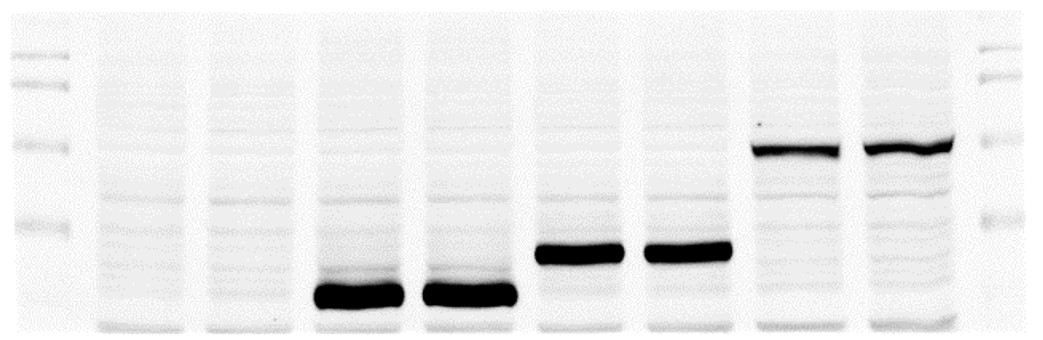

Supplement: Figure 3—source data 1. [file elife-85754-fig3-data1.zip › Figure 3- souce data 1/Fig 3A Flag.jpg]

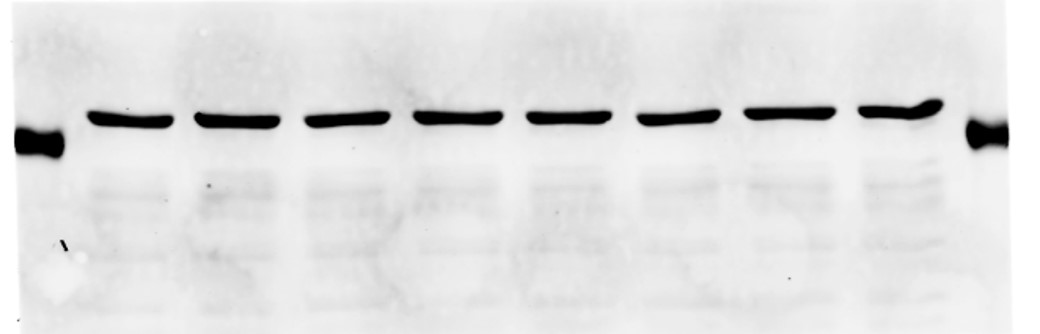

Supplement: Figure 3—source data 1. [file elife-85754-fig3-data1.zip › Figure 3- souce data 1/Fig 3A GAPDH.jpg]

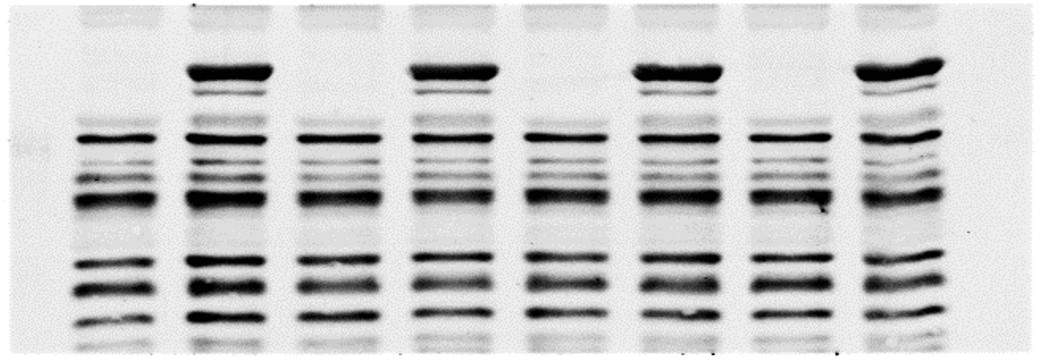

Supplement: Figure 3—source data 1. [file elife-85754-fig3-data1.zip › Figure 3- souce data 1/Fig 3A Myc.jpg]

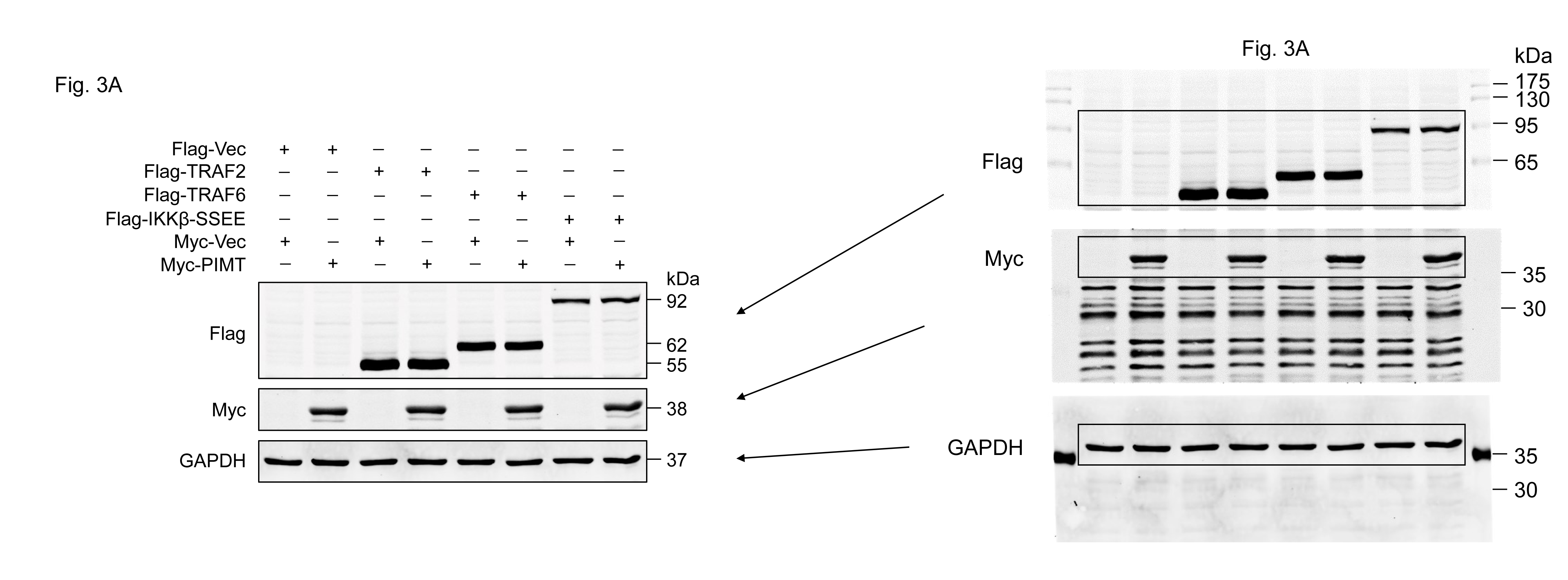

Supplement: Figure 3—source data 1. [file elife-85754-fig3-data1.zip › Figure 3- souce data 1/Figure 3.tif]

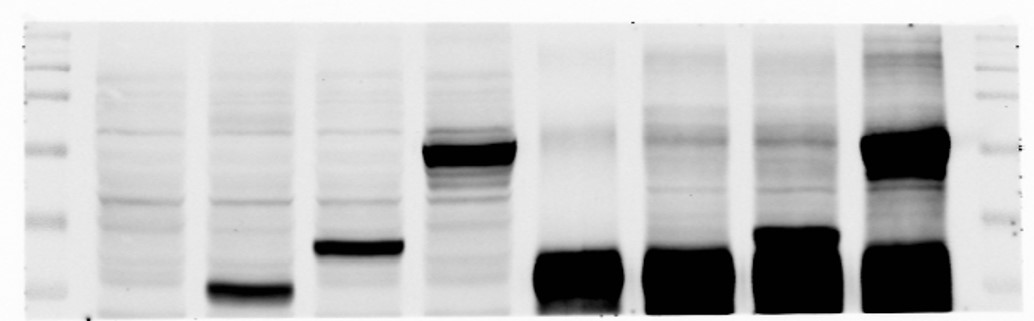

Supplement: Figure 3—source data 2. [file elife-85754-fig3-data2.zip › Figure 3- souce data 2/Fig 3B Flag.jpg]

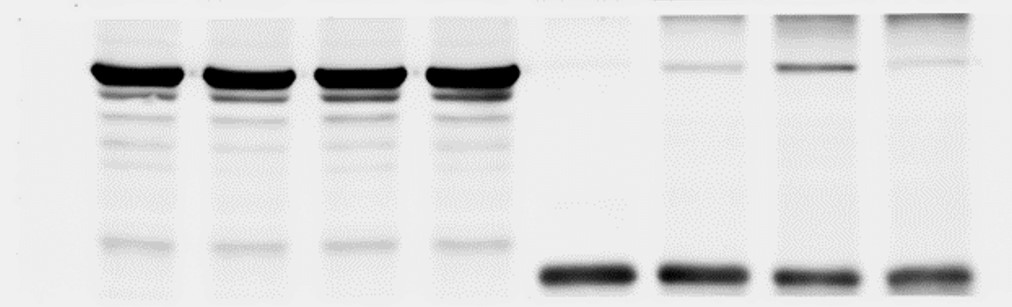

Supplement: Figure 3—source data 2. [file elife-85754-fig3-data2.zip › Figure 3- souce data 2/Fig 3B Myc.jpg]

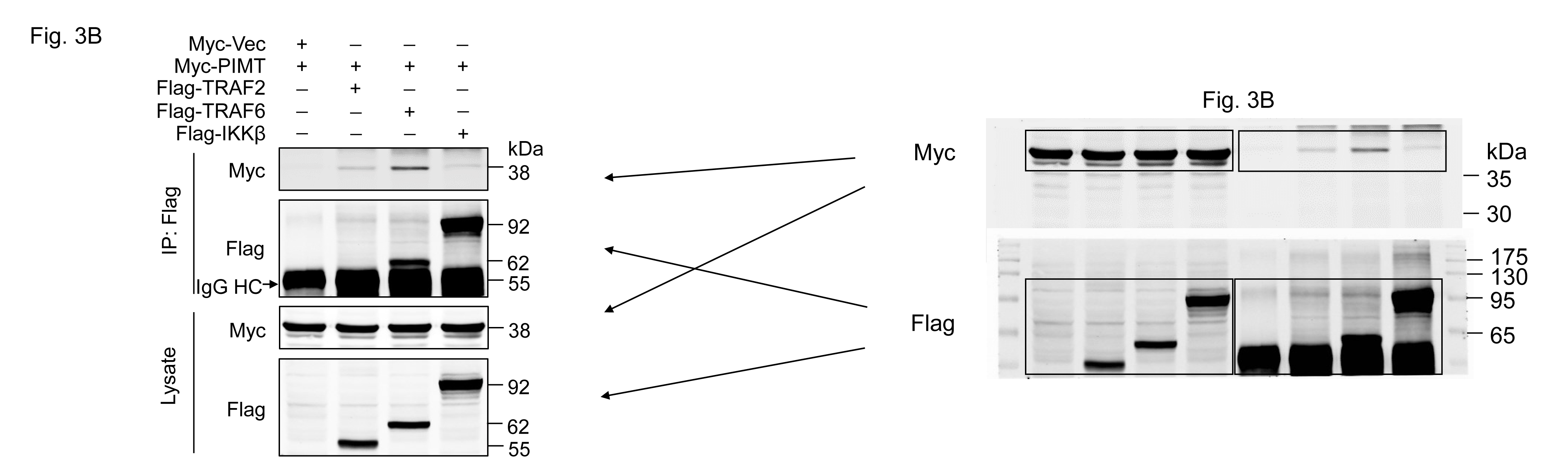

Supplement: Figure 3—source data 2. [file elife-85754-fig3-data2.zip › Figure 3- souce data 2/Figure 3B.tif]

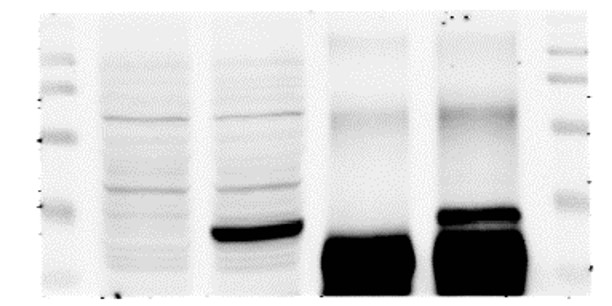

Supplement: Figure 3—source data 3. [file elife-85754-fig3-data3.zip › Figure 3- souce data 3/Fig 3C IP Flag.jpg]

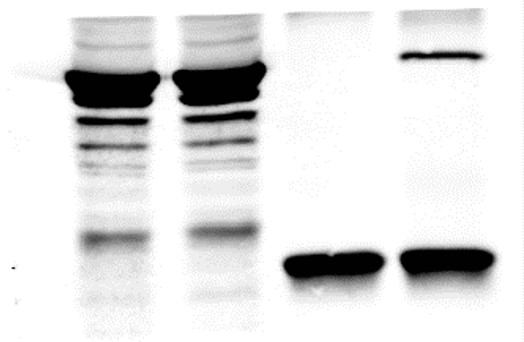

Supplement: Figure 3—source data 3. [file elife-85754-fig3-data3.zip › Figure 3- souce data 3/Fig 3C IP Myc.jpg]

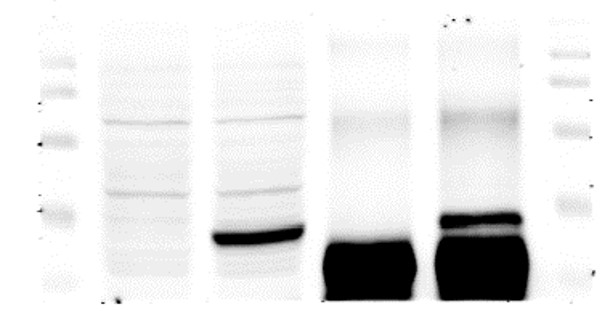

Supplement: Figure 3—source data 3. [file elife-85754-fig3-data3.zip › Figure 3- souce data 3/Fig 3C lysate Flag.jpg]

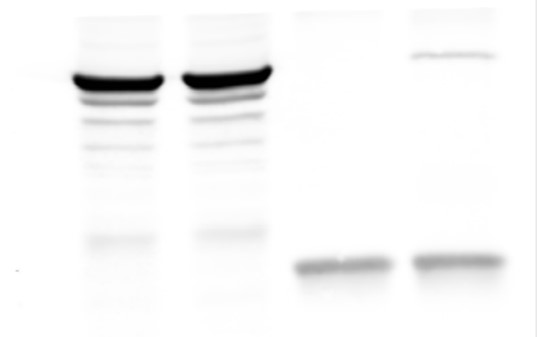

Supplement: Figure 3—source data 3. [file elife-85754-fig3-data3.zip › Figure 3- souce data 3/Fig 3C lysate Myc.jpg]

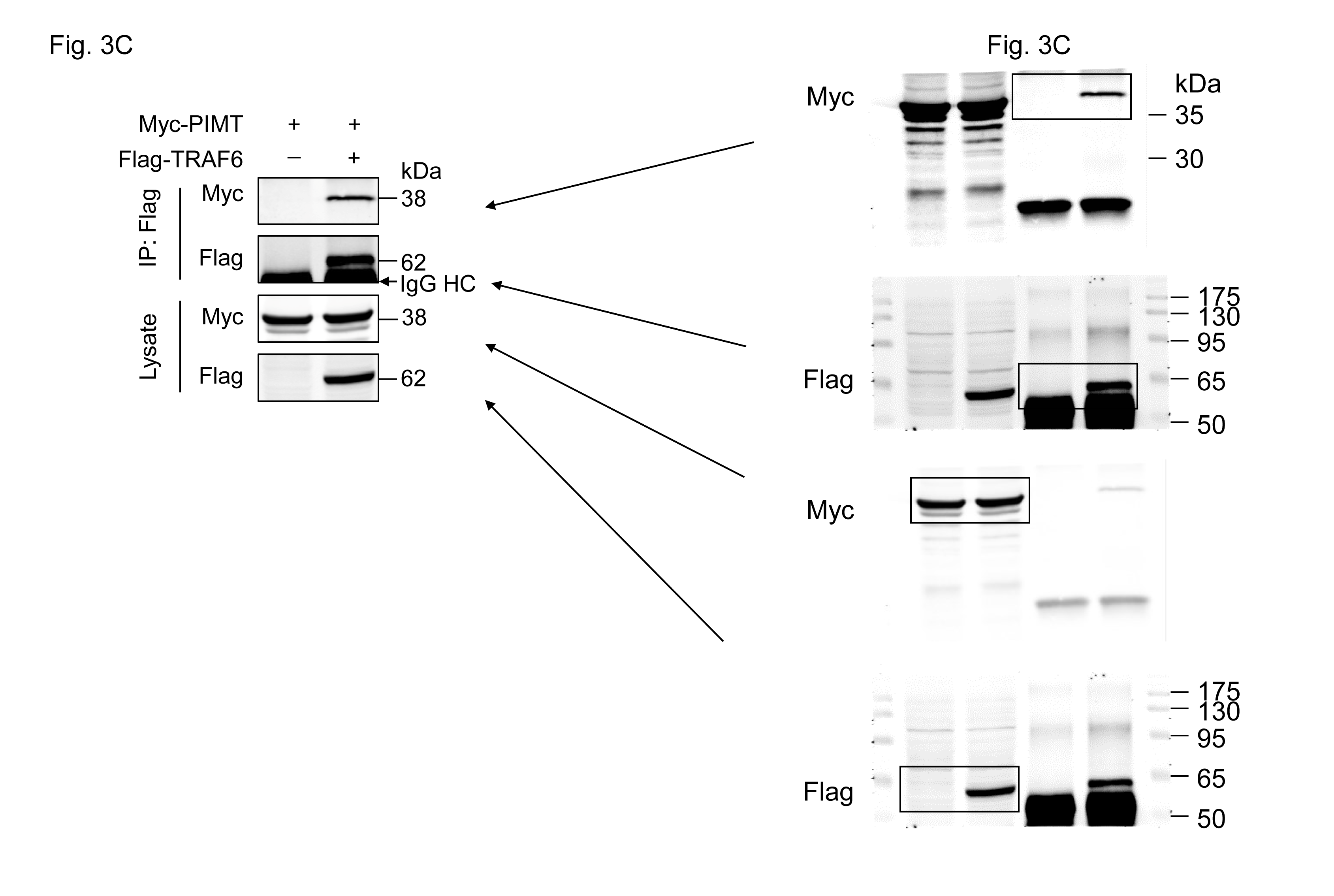

Supplement: Figure 3—source data 3. [file elife-85754-fig3-data3.zip › Figure 3- souce data 3/Figure 3C.tif]

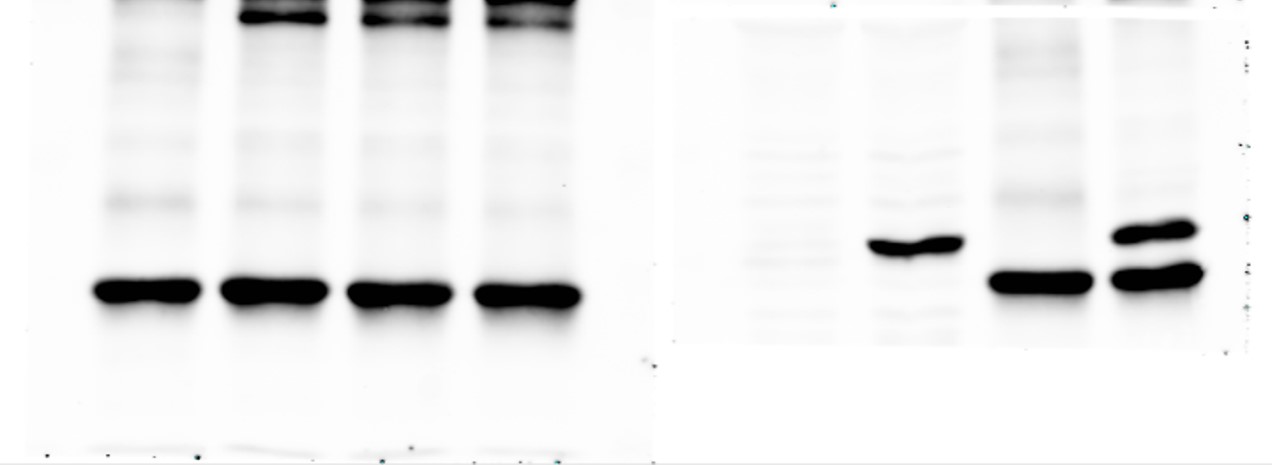

Supplement: Figure 3—source data 4. [file elife-85754-fig3-data4.zip › Figure 3- souce data 4/Fig 3D IP Flag.jpg]

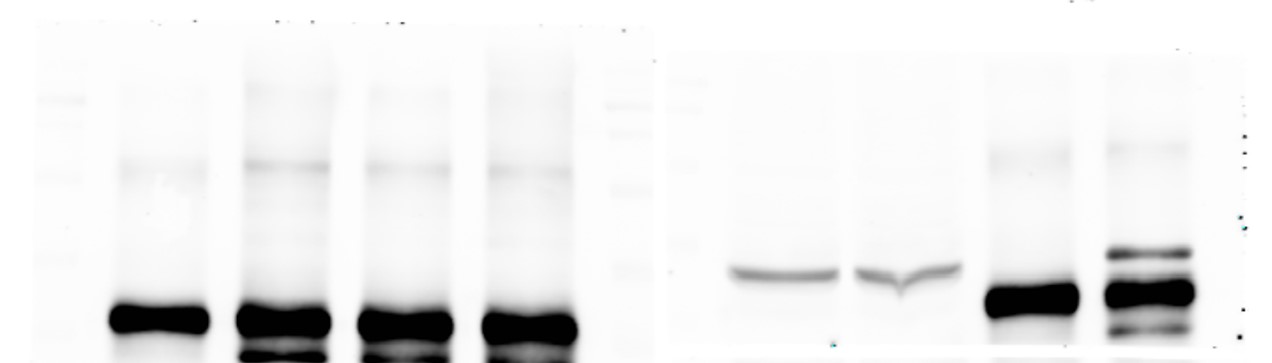

Supplement: Figure 3—source data 4. [file elife-85754-fig3-data4.zip › Figure 3- souce data 4/Fig 3D IP HA.jpg]

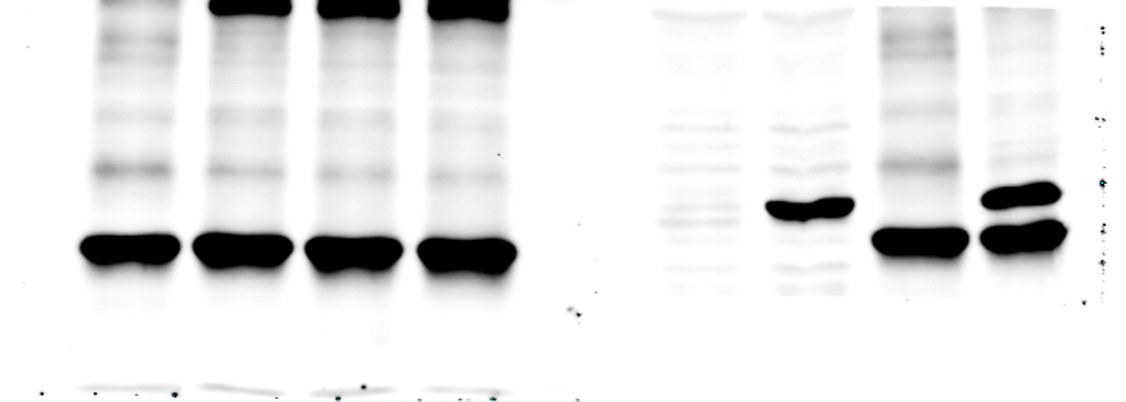

Supplement: Figure 3—source data 4. [file elife-85754-fig3-data4.zip › Figure 3- souce data 4/Fig 3D lysate Flag.jpg]

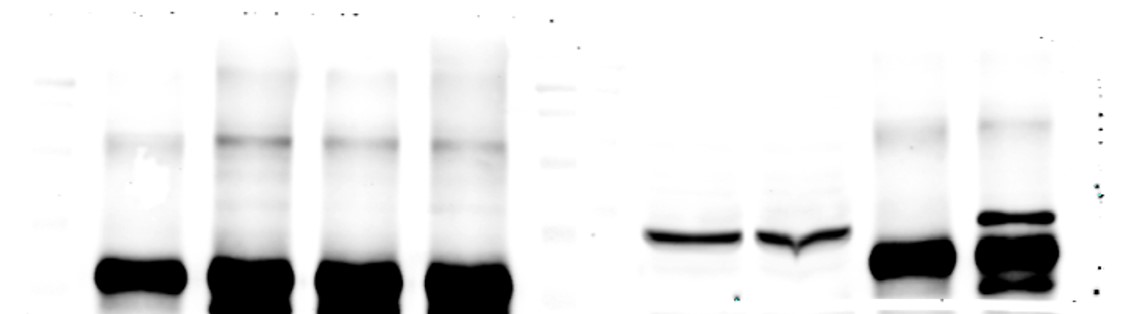

Supplement: Figure 3—source data 4. [file elife-85754-fig3-data4.zip › Figure 3- souce data 4/Fig 3D lysate HA.jpg]

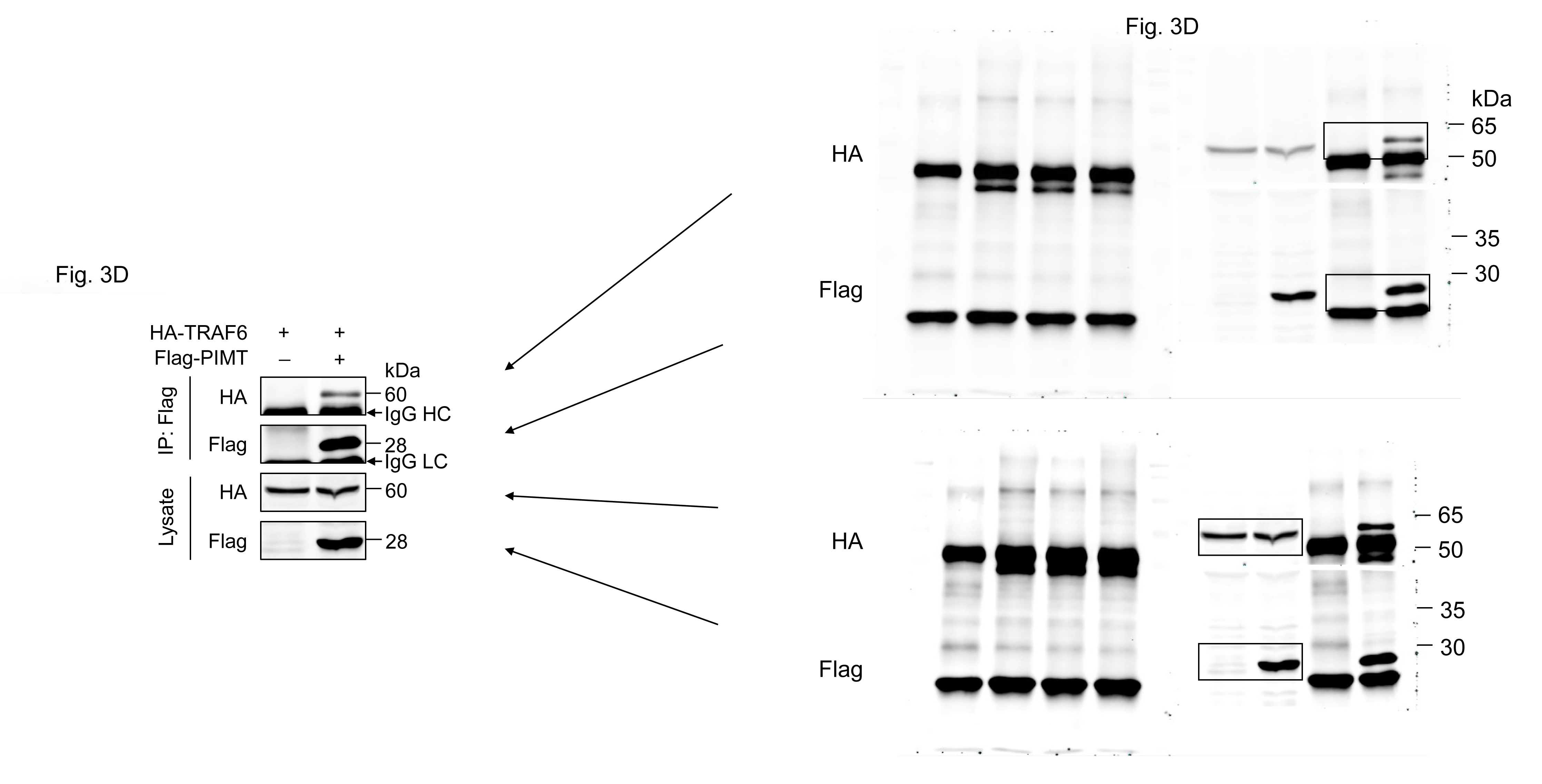

Supplement: Figure 3—source data 4. [file elife-85754-fig3-data4.zip › Figure 3- souce data 4/Figure 3D.tif]

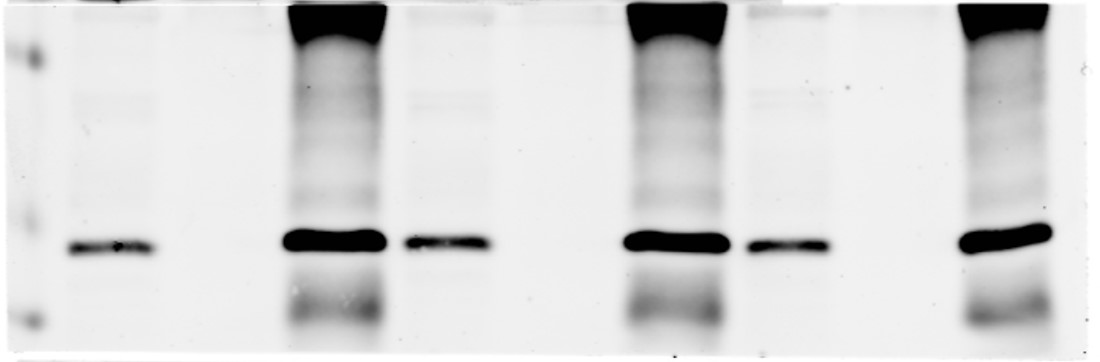

Supplement: Figure 3—source data 5. [file elife-85754-fig3-data5.zip › Figure 3- souce data 5/Fig 3E PIMT.jpg]

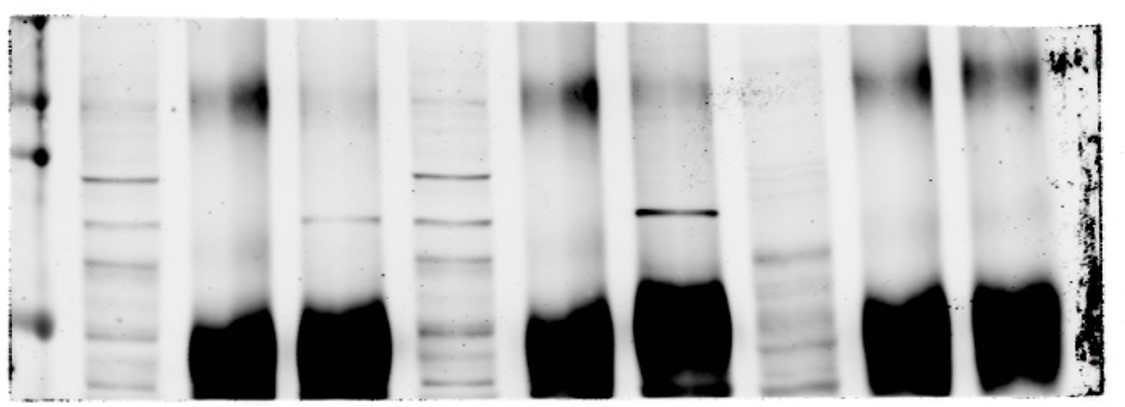

Supplement: Figure 3—source data 5. [file elife-85754-fig3-data5.zip › Figure 3- souce data 5/Fig 3E TRAF6.jpg]

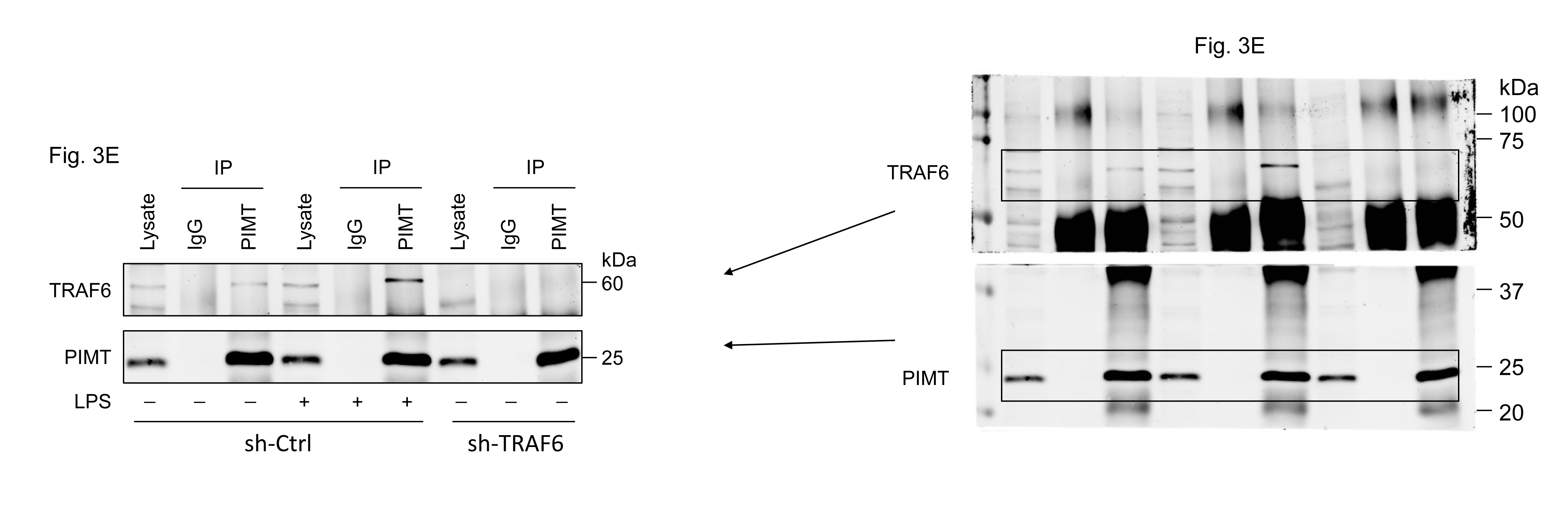

Supplement: Figure 3—source data 5. [file elife-85754-fig3-data5.zip › Figure 3- souce data 5/Figure 3E.tif]

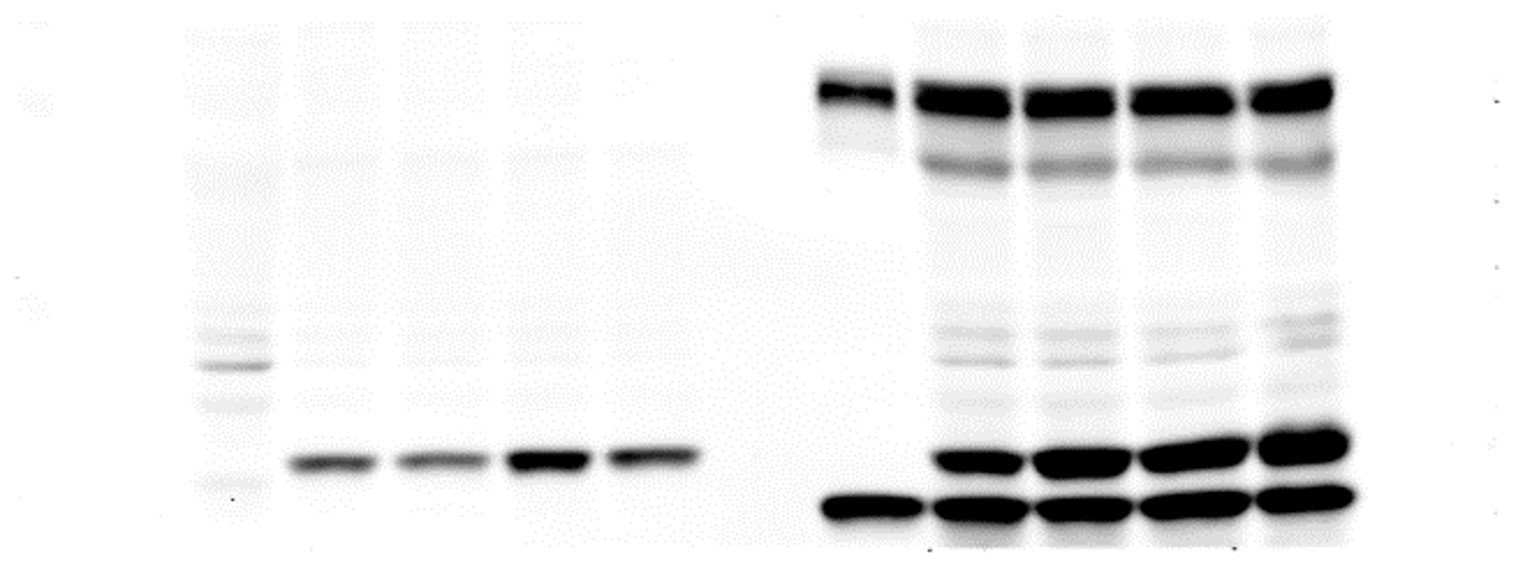

Supplement: Figure 3—source data 6. [file elife-85754-fig3-data6.zip › Figure 3- souce data 6/Fig 3F IP Flag.jpg]

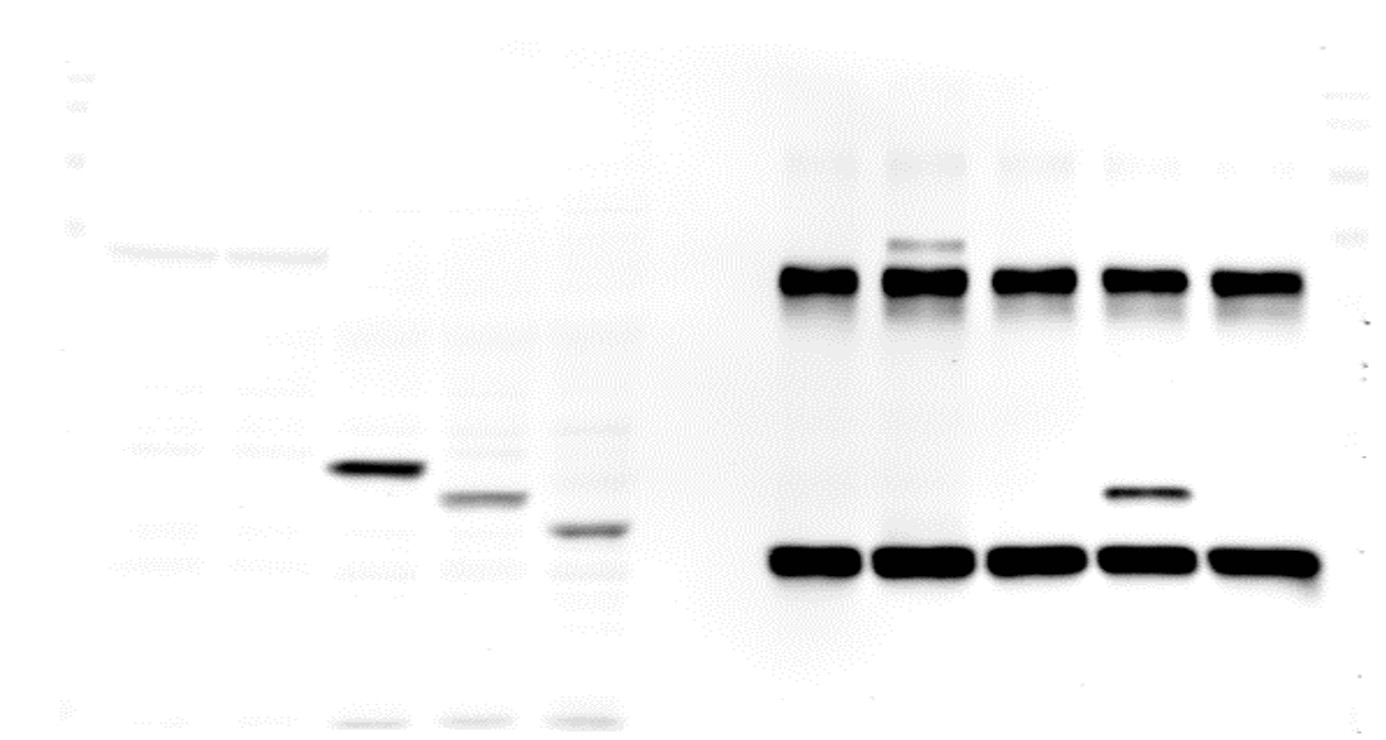

Supplement: Figure 3—source data 6. [file elife-85754-fig3-data6.zip › Figure 3- souce data 6/Fig 3F IP HA.jpg]

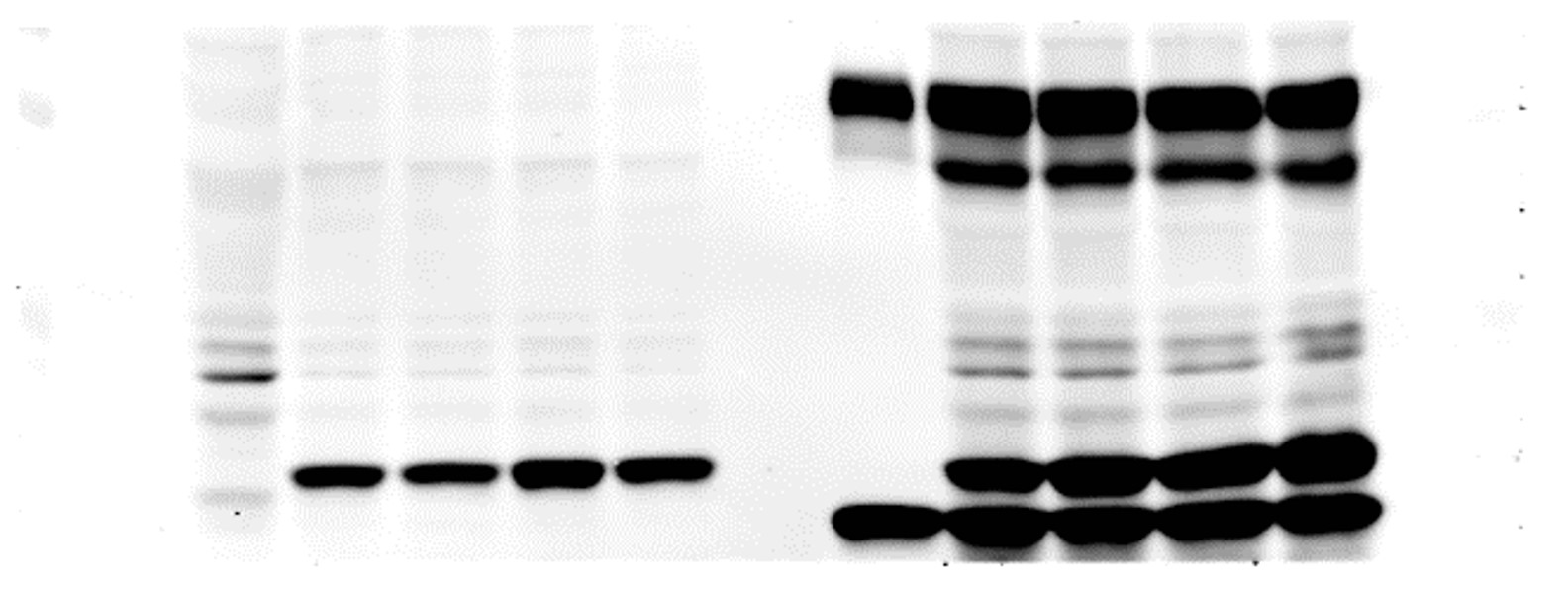

Supplement: Figure 3—source data 6. [file elife-85754-fig3-data6.zip › Figure 3- souce data 6/Fig 3F lysate Flag.jpg]

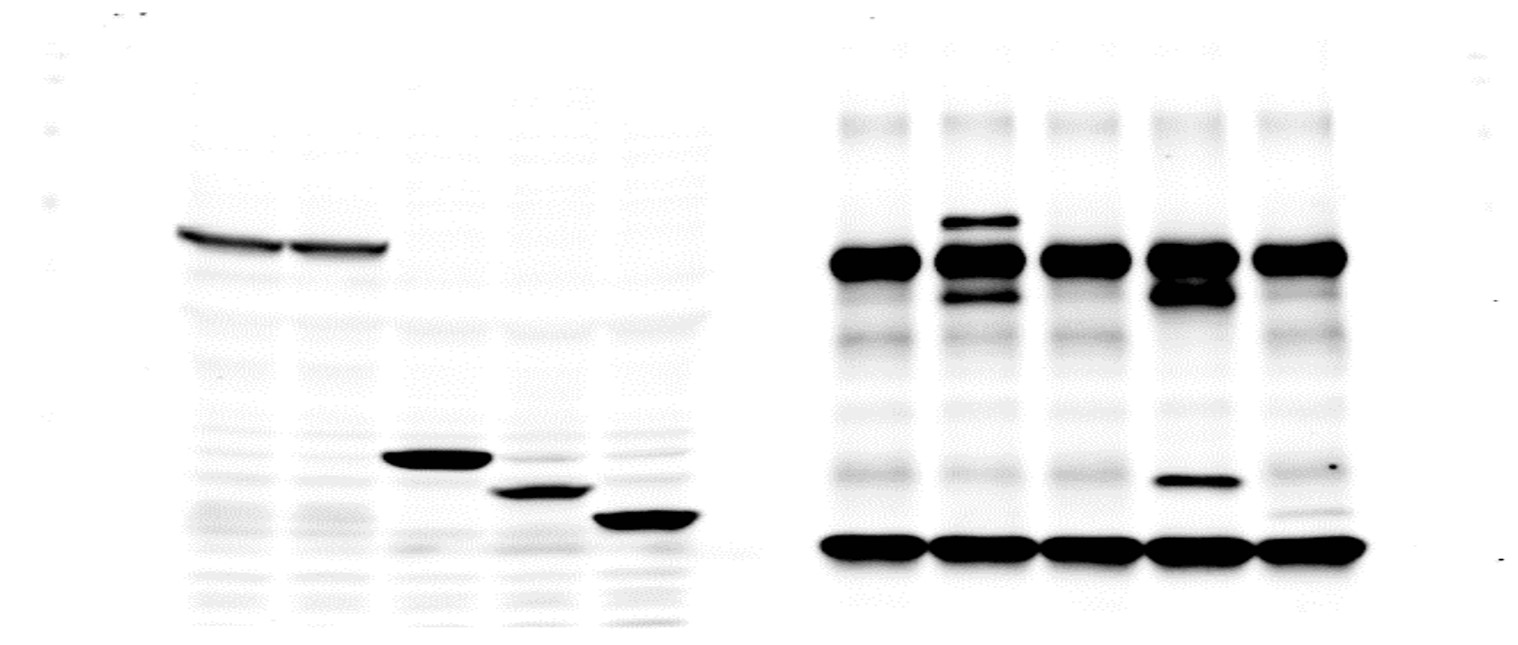

Supplement: Figure 3—source data 6. [file elife-85754-fig3-data6.zip › Figure 3- souce data 6/Fig 3F lysate HA.jpg]

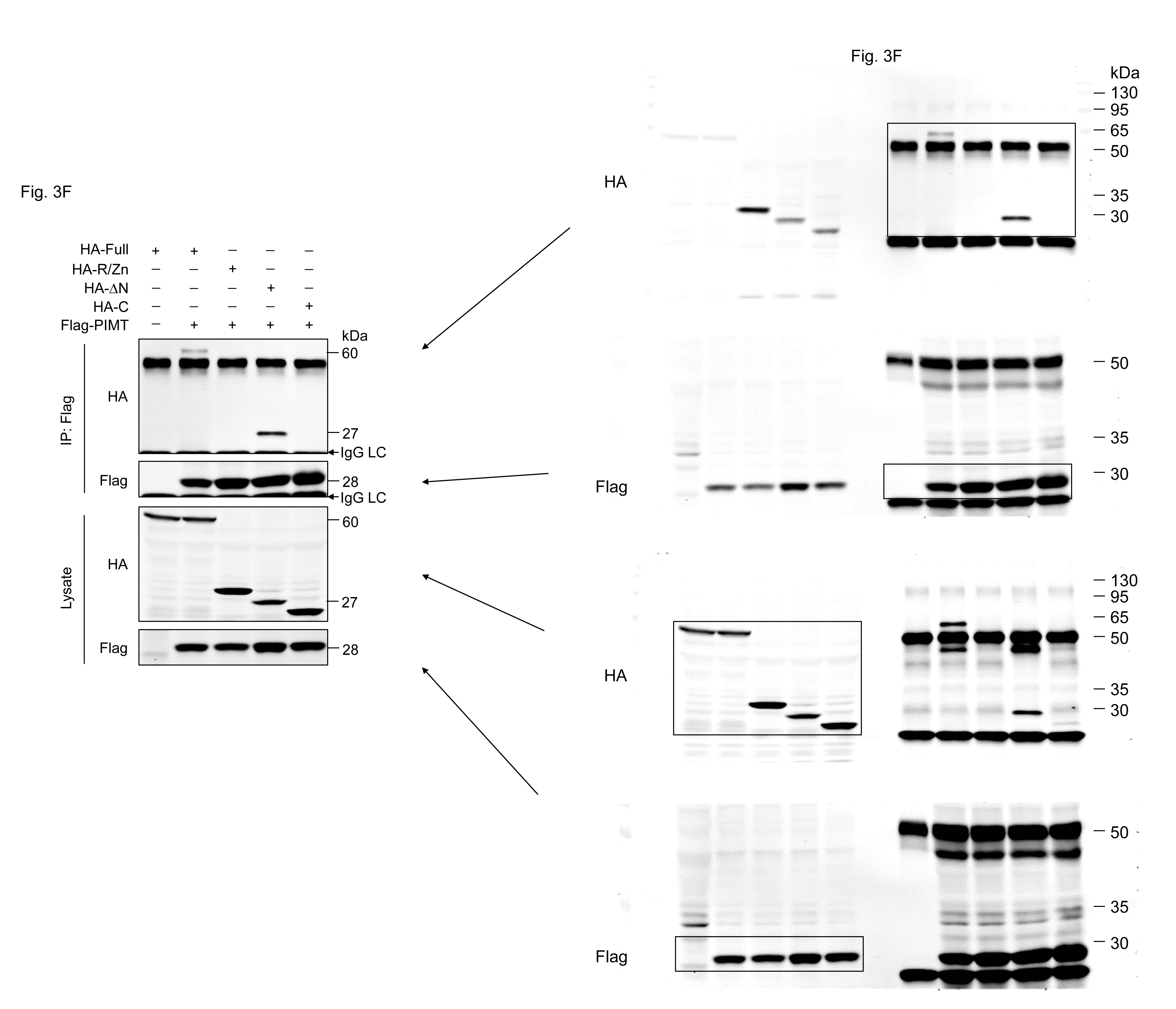

Supplement: Figure 3—source data 6. [file elife-85754-fig3-data6.zip › Figure 3- souce data 6/Figure 3F.tif]

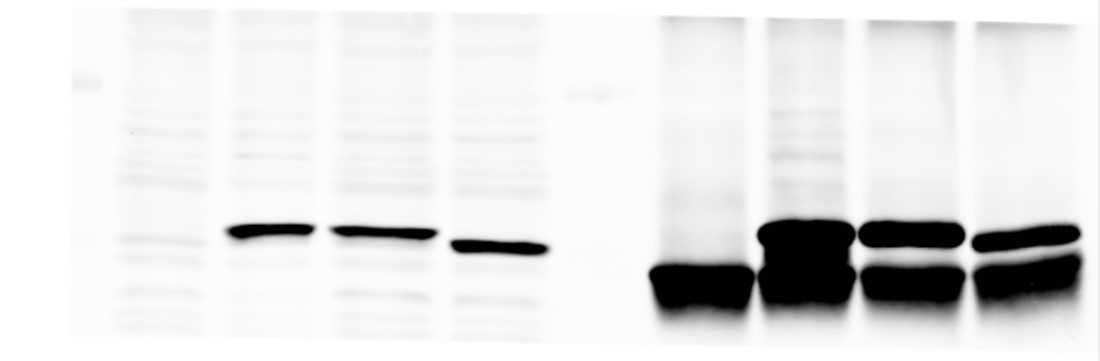

Supplement: Figure 3—source data 7. [file elife-85754-fig3-data7.zip › Figure 3- souce data 7/Fig 3G IP Flag.jpg]

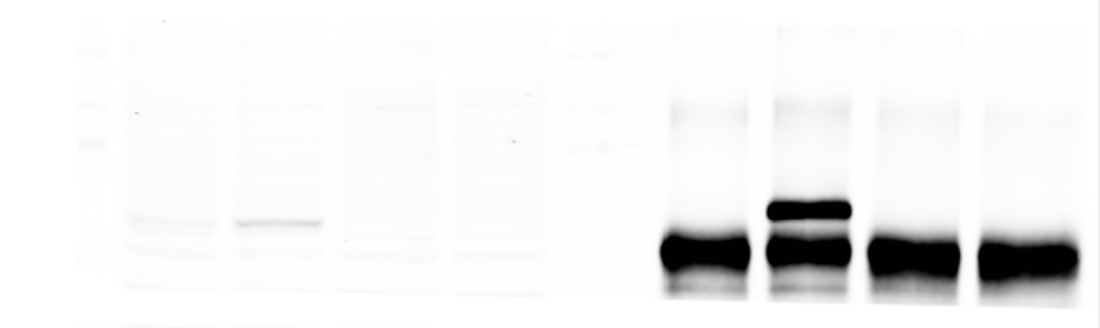

Supplement: Figure 3—source data 7. [file elife-85754-fig3-data7.zip › Figure 3- souce data 7/Fig 3G IP HA.jpg]

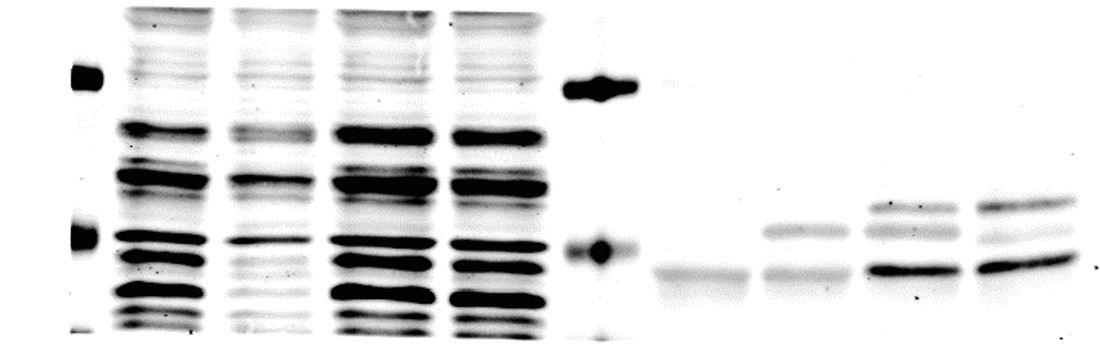

Supplement: Figure 3—source data 7. [file elife-85754-fig3-data7.zip › Figure 3- souce data 7/Fig 3G IP Myc.jpg]

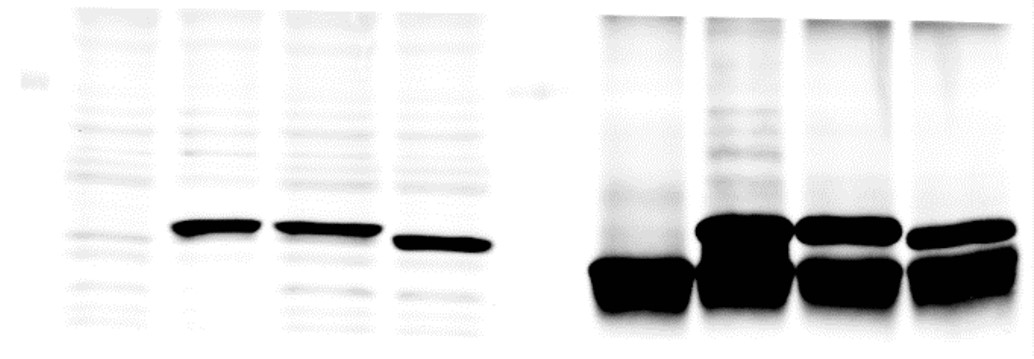

Supplement: Figure 3—source data 7. [file elife-85754-fig3-data7.zip › Figure 3- souce data 7/Fig 3G lysate Flag.jpg]

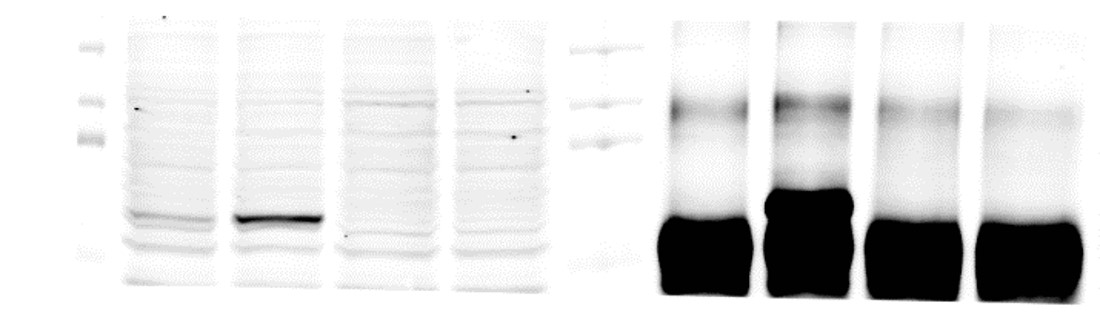

Supplement: Figure 3—source data 7. [file elife-85754-fig3-data7.zip › Figure 3- souce data 7/Fig 3G lysate HA.jpg]

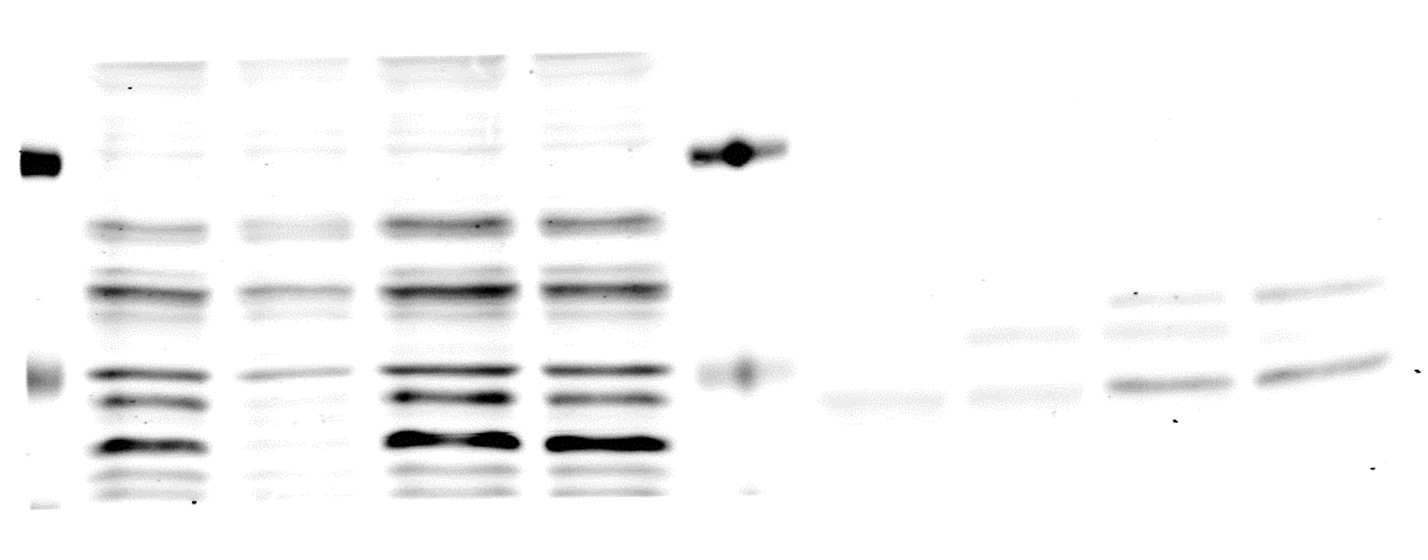

Supplement: Figure 3—source data 7. [file elife-85754-fig3-data7.zip › Figure 3- souce data 7/Fig 3G lysate Myc.jpg]

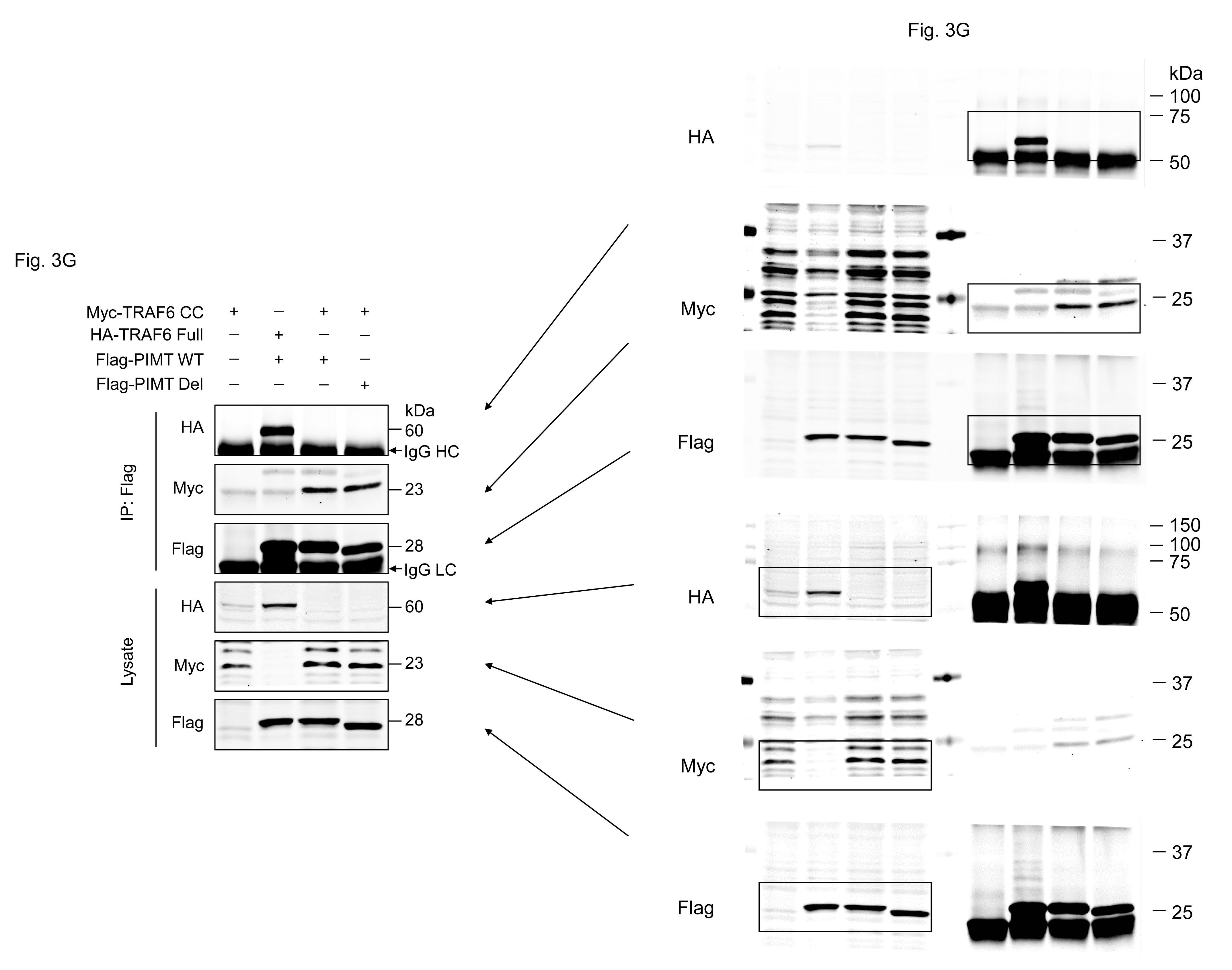

Supplement: Figure 3—source data 7. [file elife-85754-fig3-data7.zip › Figure 3- souce data 7/Figure 3G.tif]

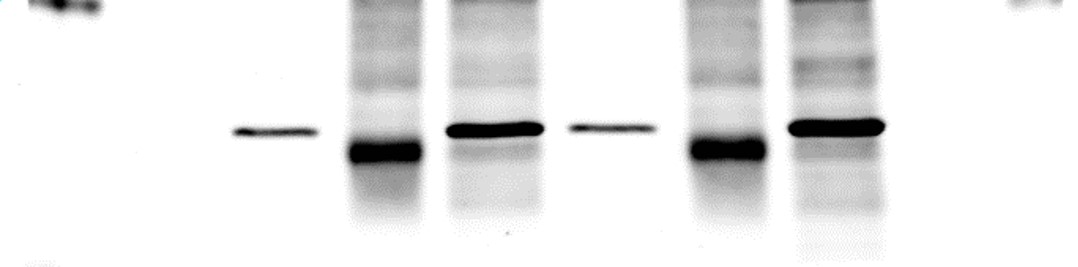

Supplement: Figure 3—figure supplement 1—source data 1. [file elife-85754-fig3-figsupp1-data1.zip › Figure 3-figure supplement 1-source data 1/Fig 3 S1A PIMT.jpg]

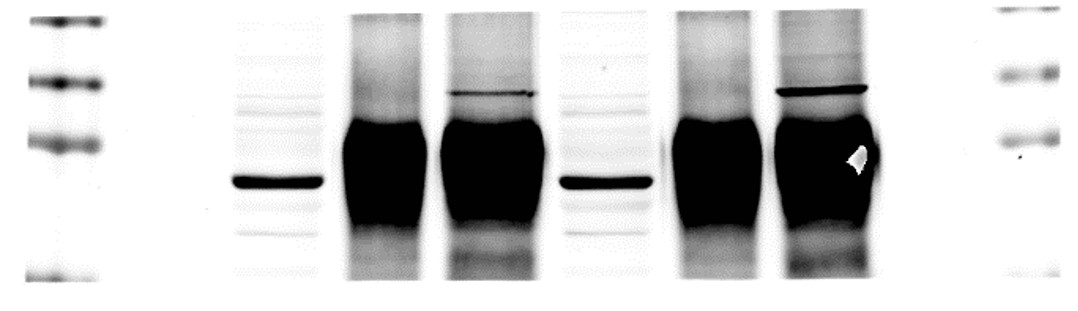

Supplement: Figure 3—figure supplement 1—source data 1. [file elife-85754-fig3-figsupp1-data1.zip › Figure 3-figure supplement 1-source data 1/Fig 3 S1A TRAF6.jpg]

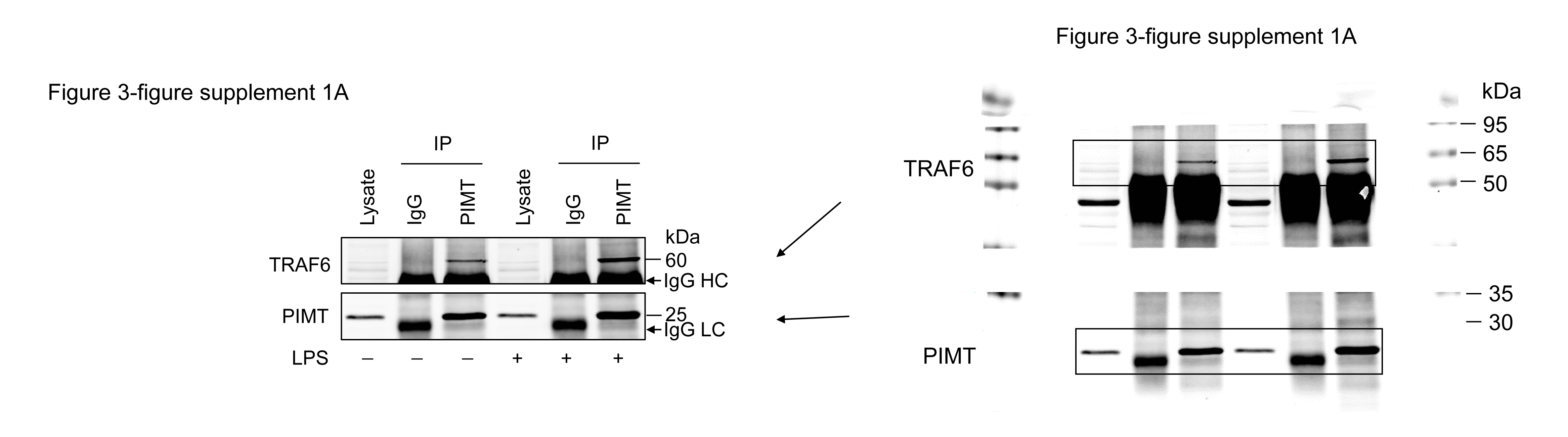

Supplement: Figure 3—figure supplement 1—source data 1. [file elife-85754-fig3-figsupp1-data1.zip › Figure 3-figure supplement 1-source data 1/Figure 3-figure supplement 1A.tif]

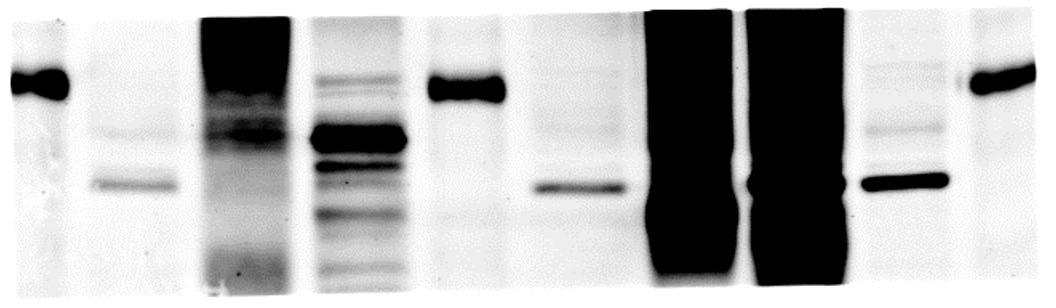

Supplement: Figure 3—figure supplement 1—source data 2. [file elife-85754-fig3-figsupp1-data2.zip › Figure 3-figure supplement 1-source data 2/Fig3 S1B PIMT.jpg]

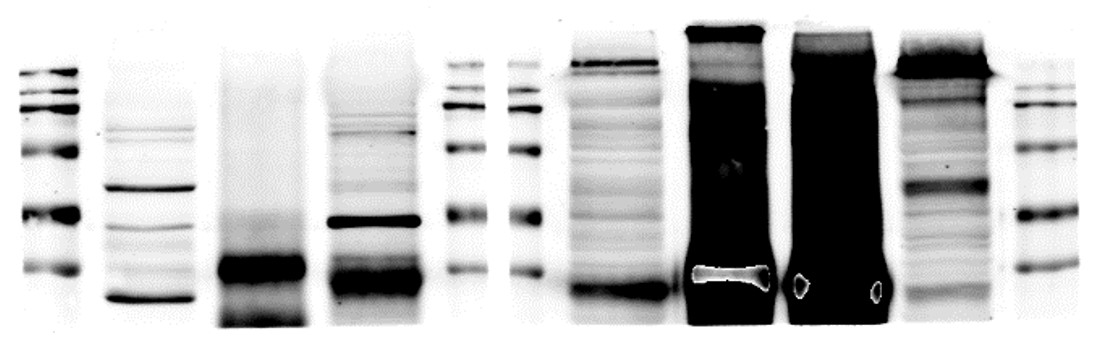

Supplement: Figure 3—figure supplement 1—source data 2. [file elife-85754-fig3-figsupp1-data2.zip › Figure 3-figure supplement 1-source data 2/Fig3 S1B TRAF6.jpg]

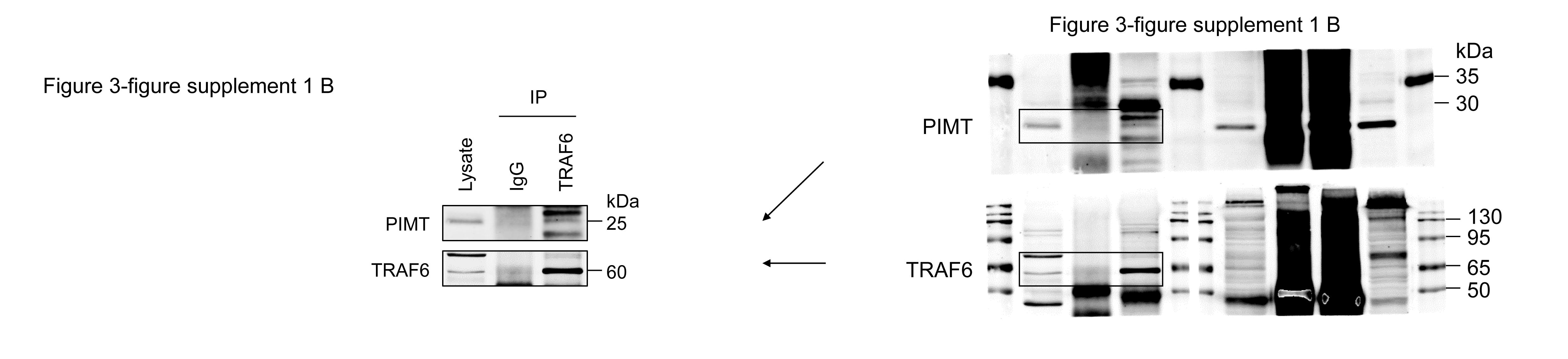

Supplement: Figure 3—figure supplement 1—source data 2. [file elife-85754-fig3-figsupp1-data2.zip › Figure 3-figure supplement 1-source data 2/Figure 3-figure supplement 1B.tif]

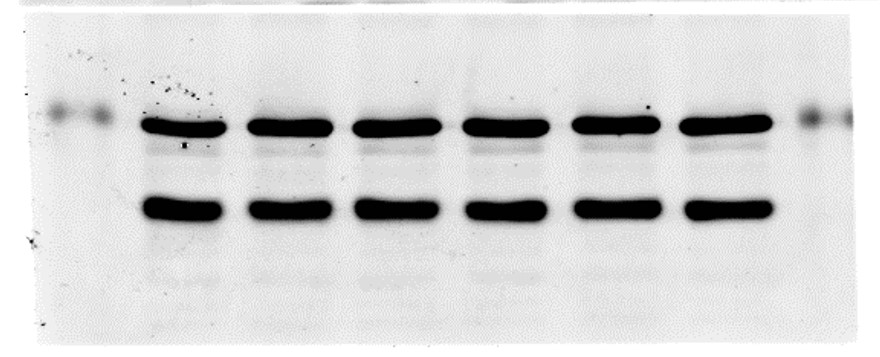

Supplement: Figure 3—figure supplement 1—source data 3. [file elife-85754-fig3-figsupp1-data3.zip › Figure 3-figure supplement 1-source data 3/Fig 3 S1C GAPDH.jpg]

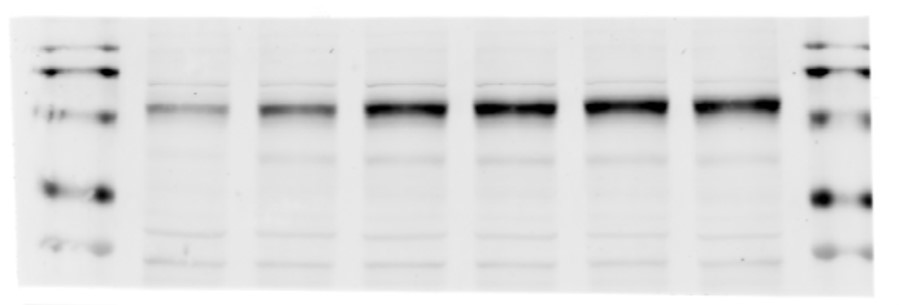

Supplement: Figure 3—figure supplement 1—source data 3. [file elife-85754-fig3-figsupp1-data3.zip › Figure 3-figure supplement 1-source data 3/Fig 3 S1C ICAM-1.jpg]

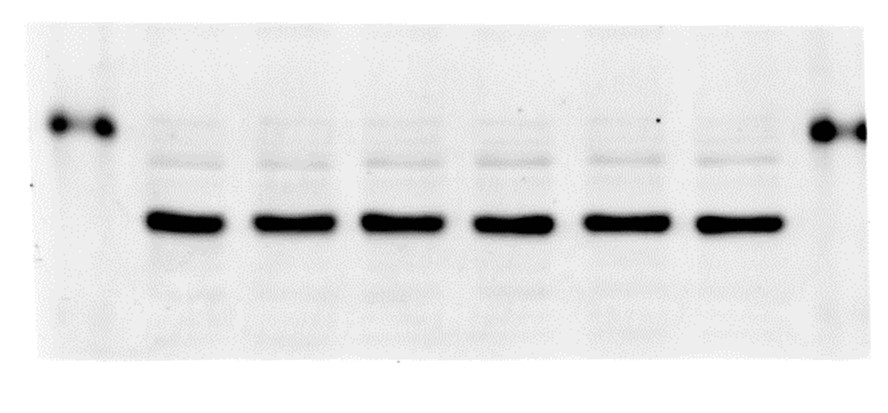

Supplement: Figure 3—figure supplement 1—source data 3. [file elife-85754-fig3-figsupp1-data3.zip › Figure 3-figure supplement 1-source data 3/Fig 3 S1C PIMT.jpg]

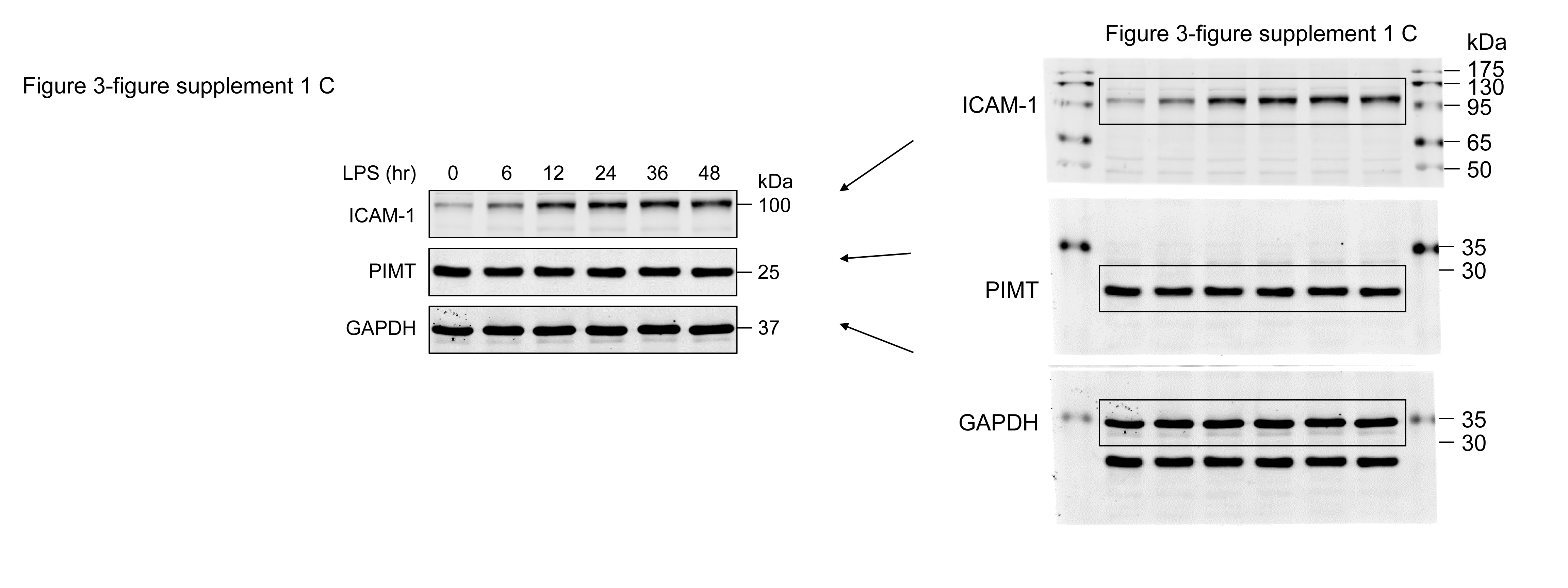

Supplement: Figure 3—figure supplement 1—source data 3. [file elife-85754-fig3-figsupp1-data3.zip › Figure 3-figure supplement 1-source data 3/Figure 3-figure supplement 1C.tif]

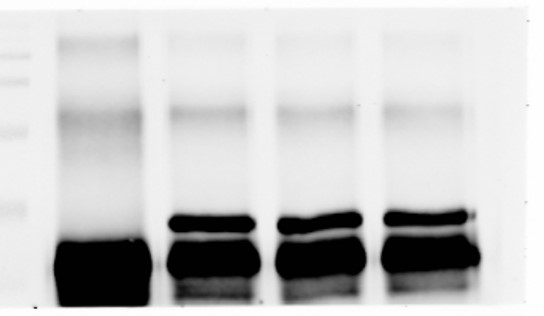

Supplement: Figure 4—source data 1. [file elife-85754-fig4-data1.zip › Figure 4- souce data 1/Fig 4A IP Flag.jpg]

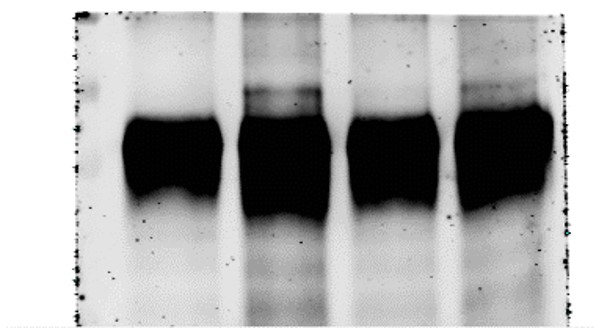

Supplement: Figure 4—source data 1. [file elife-85754-fig4-data1.zip › Figure 4- souce data 1/Fig 4A IP HA.jpg]

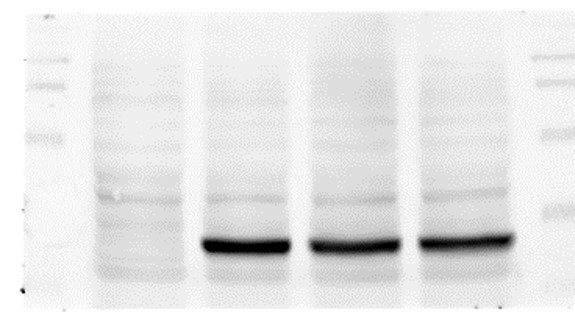

Supplement: Figure 4—source data 1. [file elife-85754-fig4-data1.zip › Figure 4- souce data 1/Fig 4A lysate Flag.jpg]

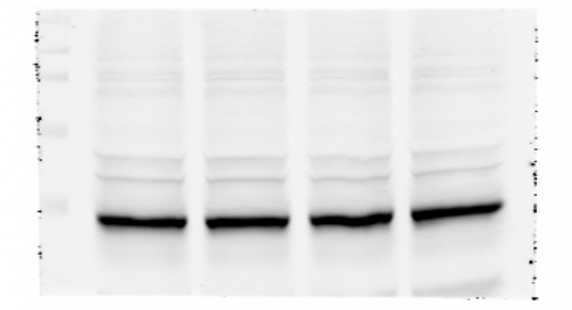

Supplement: Figure 4—source data 1. [file elife-85754-fig4-data1.zip › Figure 4- souce data 1/Fig 4A lysate HA.jpg]

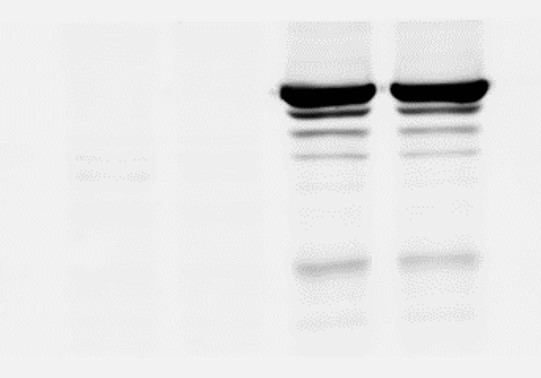

Supplement: Figure 4—source data 1. [file elife-85754-fig4-data1.zip › Figure 4- souce data 1/Fig 4A lysate Myc.jpg]

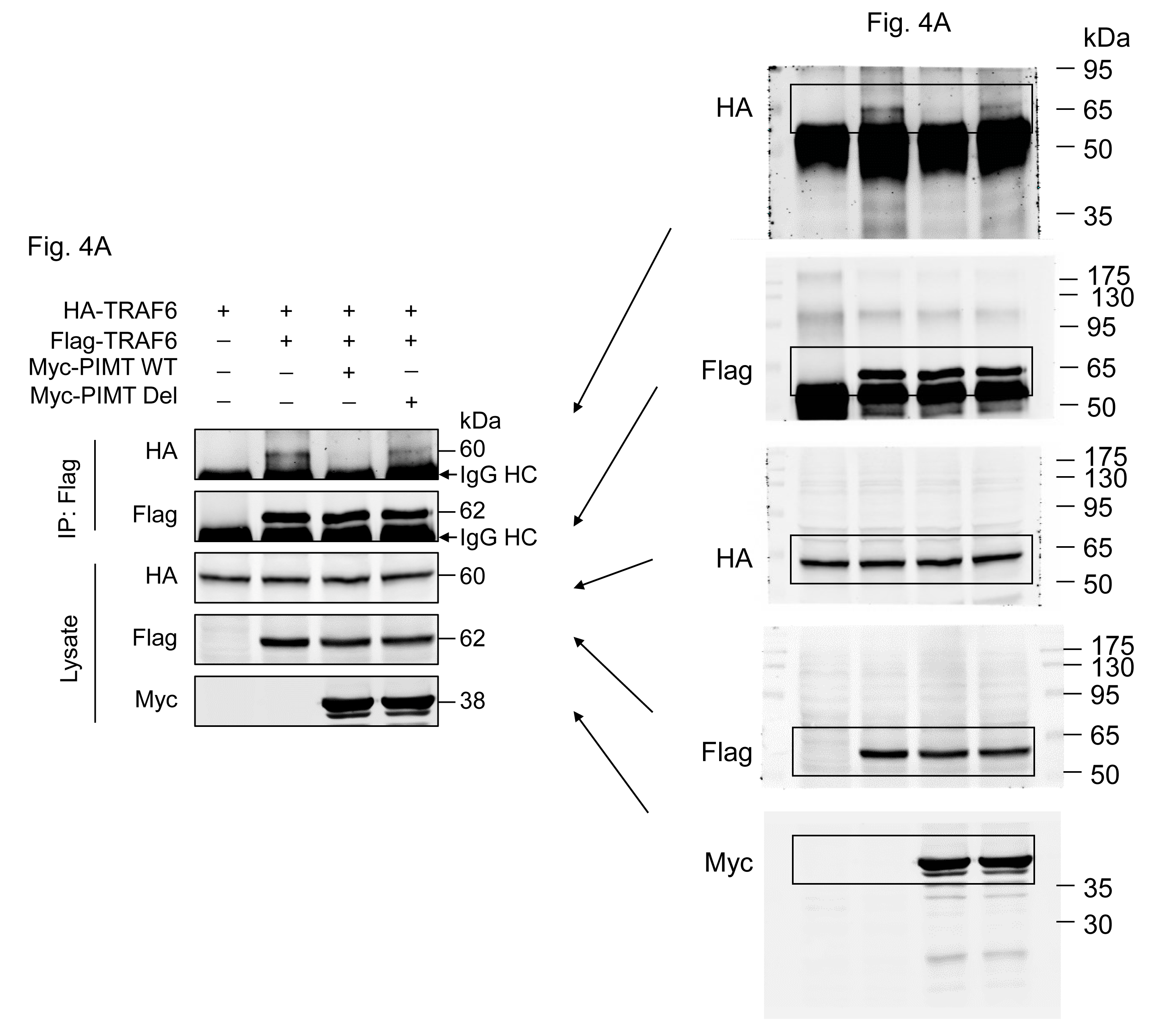

Supplement: Figure 4—source data 1. [file elife-85754-fig4-data1.zip › Figure 4- souce data 1/Figure 4A.tif]

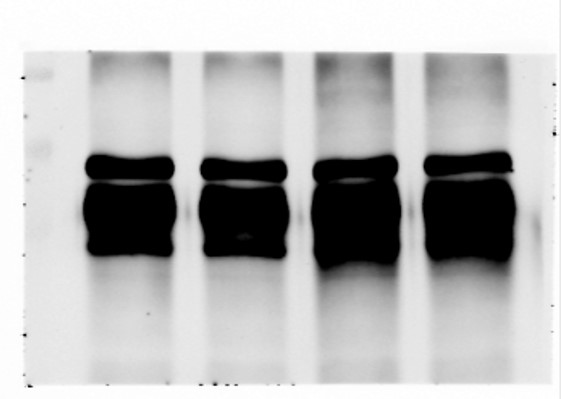

Supplement: Figure 4—source data 2. [file elife-85754-fig4-data2.zip › Figure 4- souce data 2/Fig 4B IP Flag.jpg]

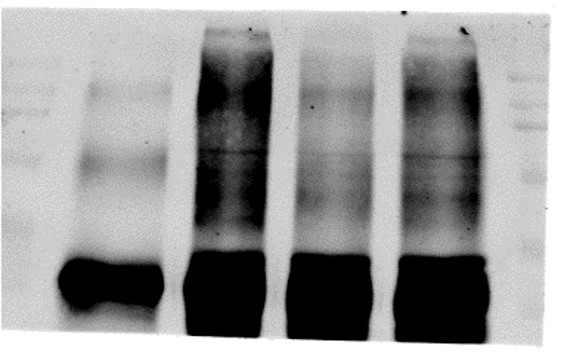

Supplement: Figure 4—source data 2. [file elife-85754-fig4-data2.zip › Figure 4- souce data 2/Fig 4B IP HA.jpg]

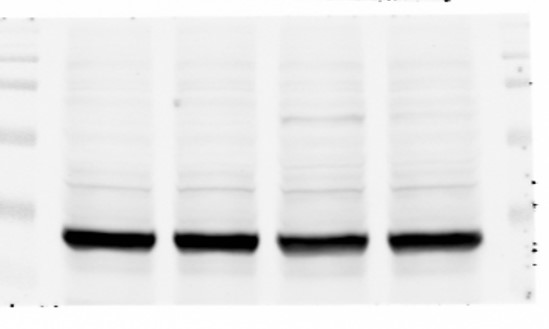

Supplement: Figure 4—source data 2. [file elife-85754-fig4-data2.zip › Figure 4- souce data 2/Fig 4B lysate Flag.jpg]

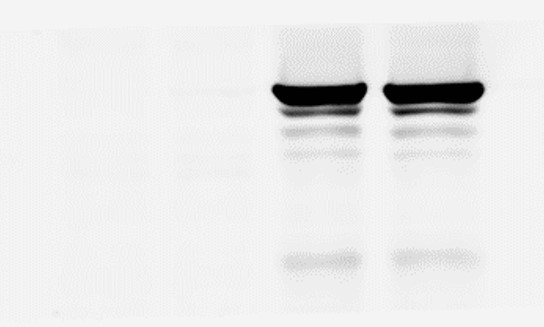

Supplement: Figure 4—source data 2. [file elife-85754-fig4-data2.zip › Figure 4- souce data 2/Fig 4B lysate Myc.jpg]

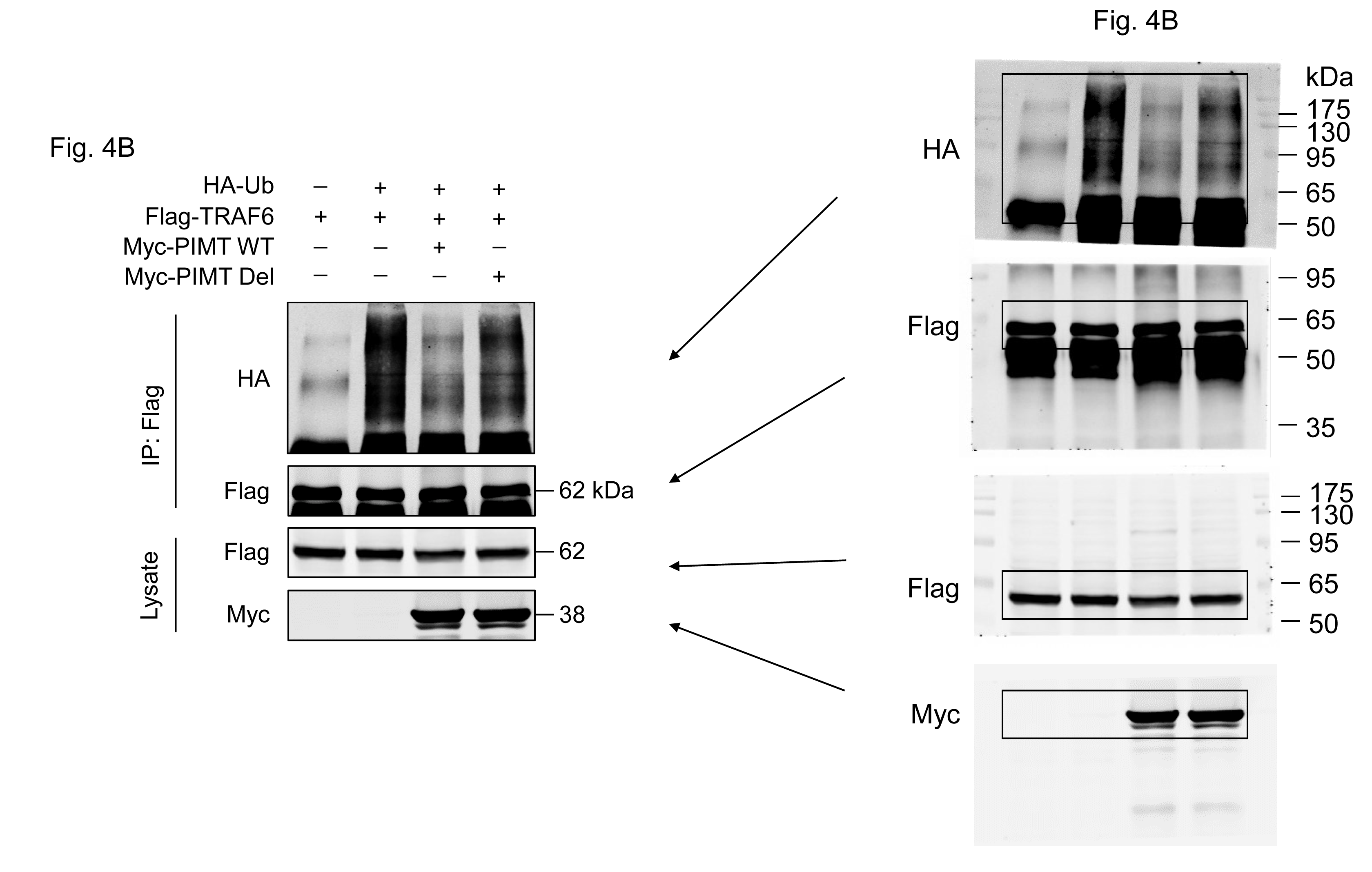

Supplement: Figure 4—source data 2. [file elife-85754-fig4-data2.zip › Figure 4- souce data 2/Figure 4B.tif]

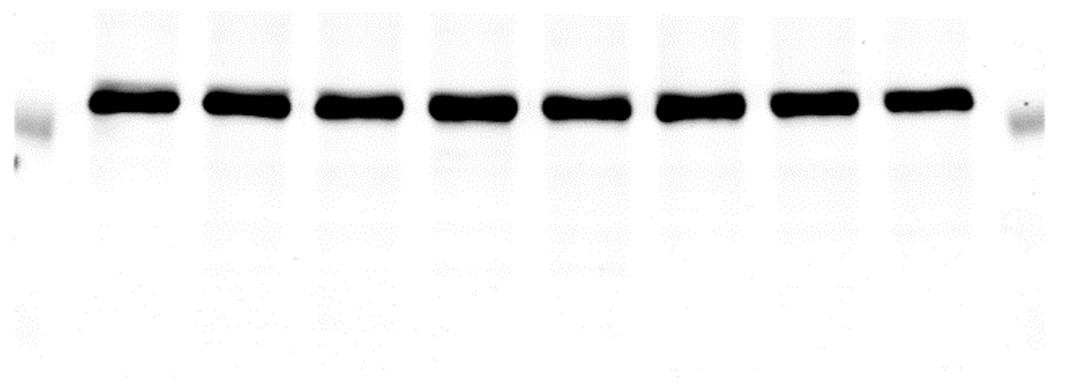

Supplement: Figure 4—source data 3. [file elife-85754-fig4-data3.zip › Figure 4- souce data 3/Fig 4C GAPDH.jpg]

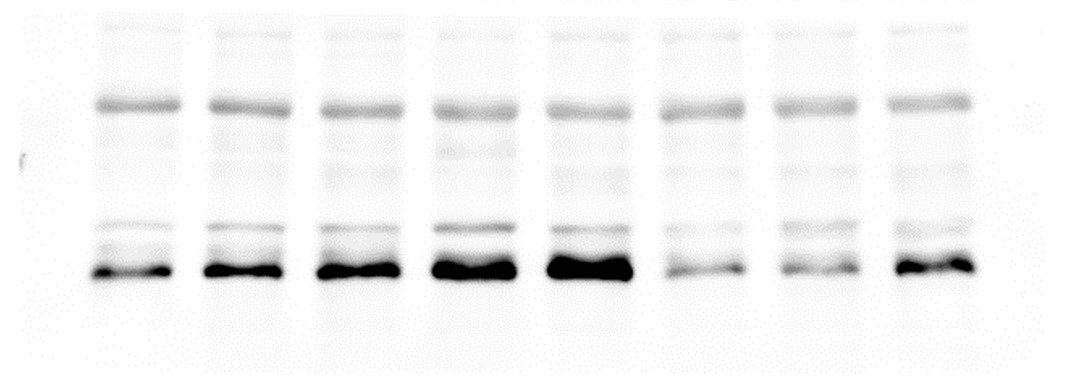

Supplement: Figure 4—source data 3. [file elife-85754-fig4-data3.zip › Figure 4- souce data 3/Fig 4C PIMT.jpg]

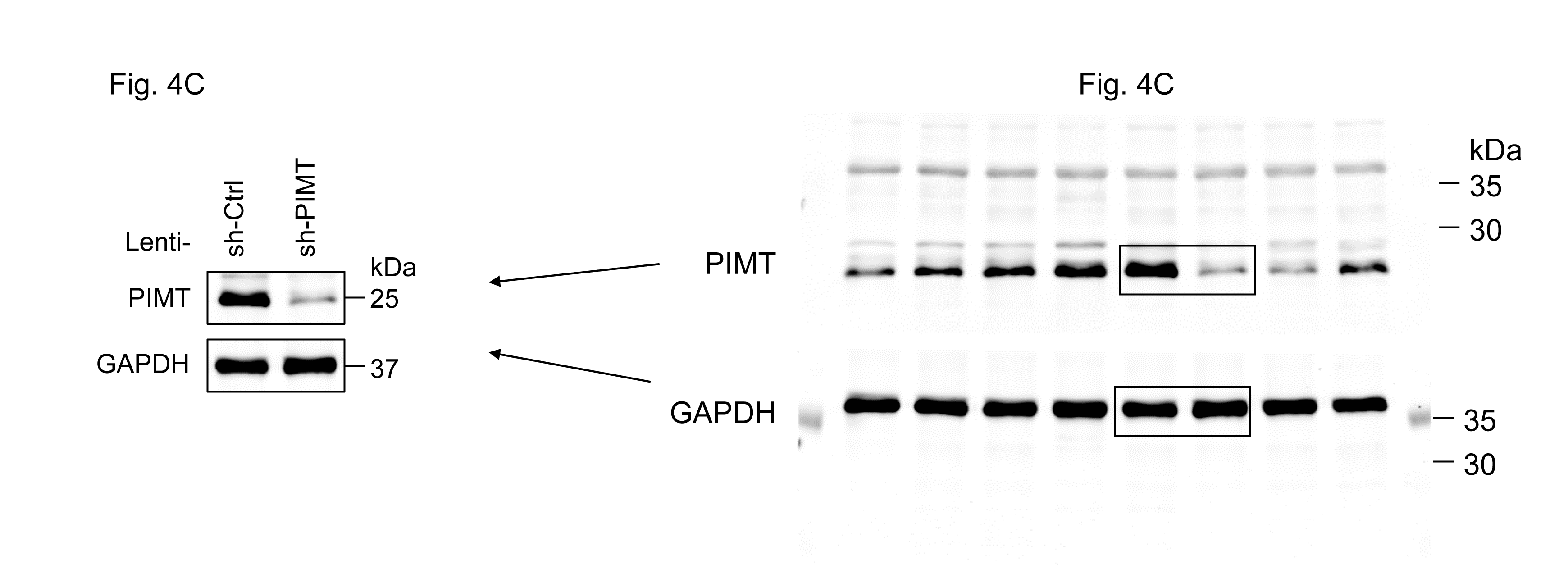

Supplement: Figure 4—source data 3. [file elife-85754-fig4-data3.zip › Figure 4- souce data 3/Figure 4C.tif]

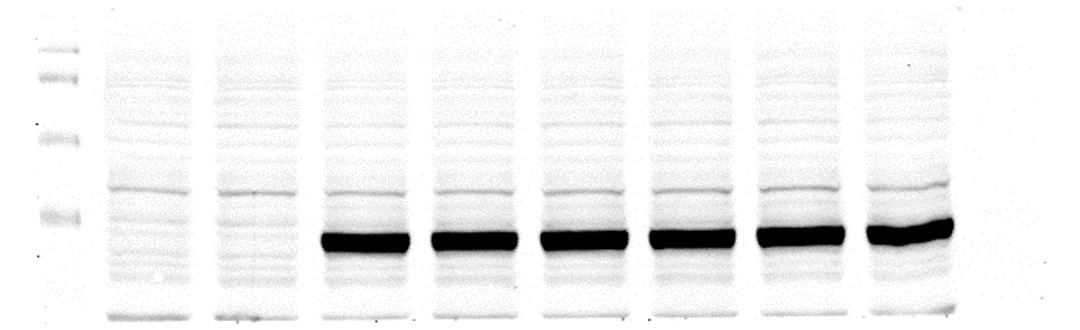

Supplement: Figure 4—source data 4. [file elife-85754-fig4-data4.zip › Figure 4- souce data 4/Fig 4F Flag.jpg]

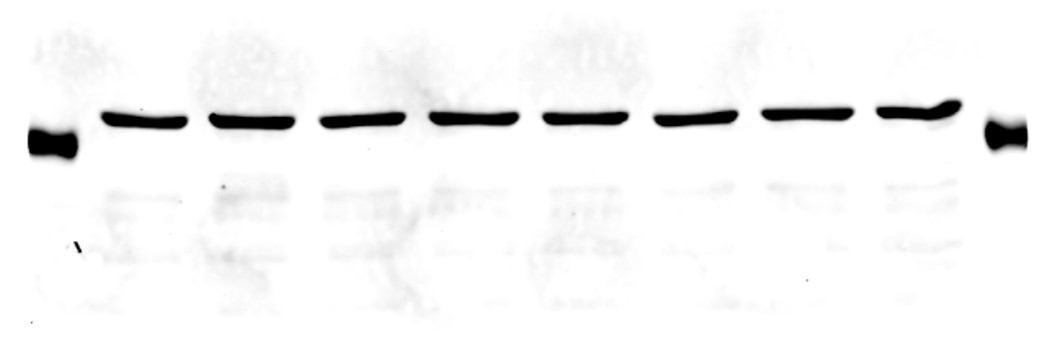

Supplement: Figure 4—source data 4. [file elife-85754-fig4-data4.zip › Figure 4- souce data 4/Fig 4F GAPDH.jpg]

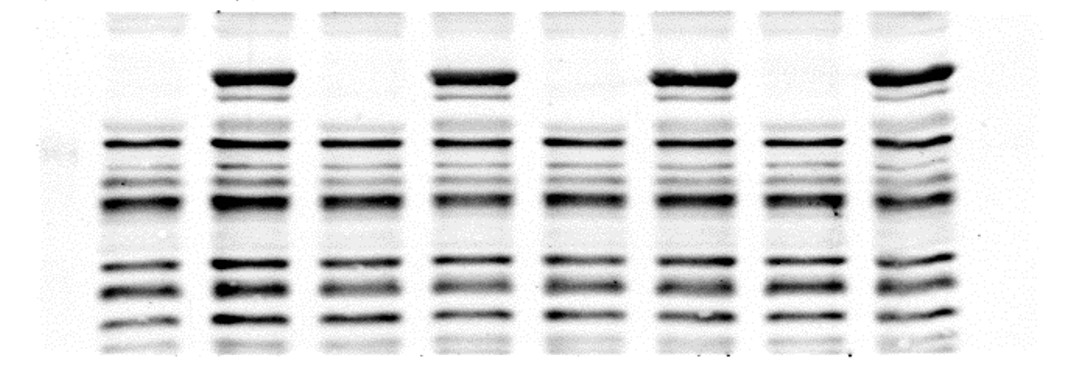

Supplement: Figure 4—source data 4. [file elife-85754-fig4-data4.zip › Figure 4- souce data 4/Fig 4F Myc.jpg]

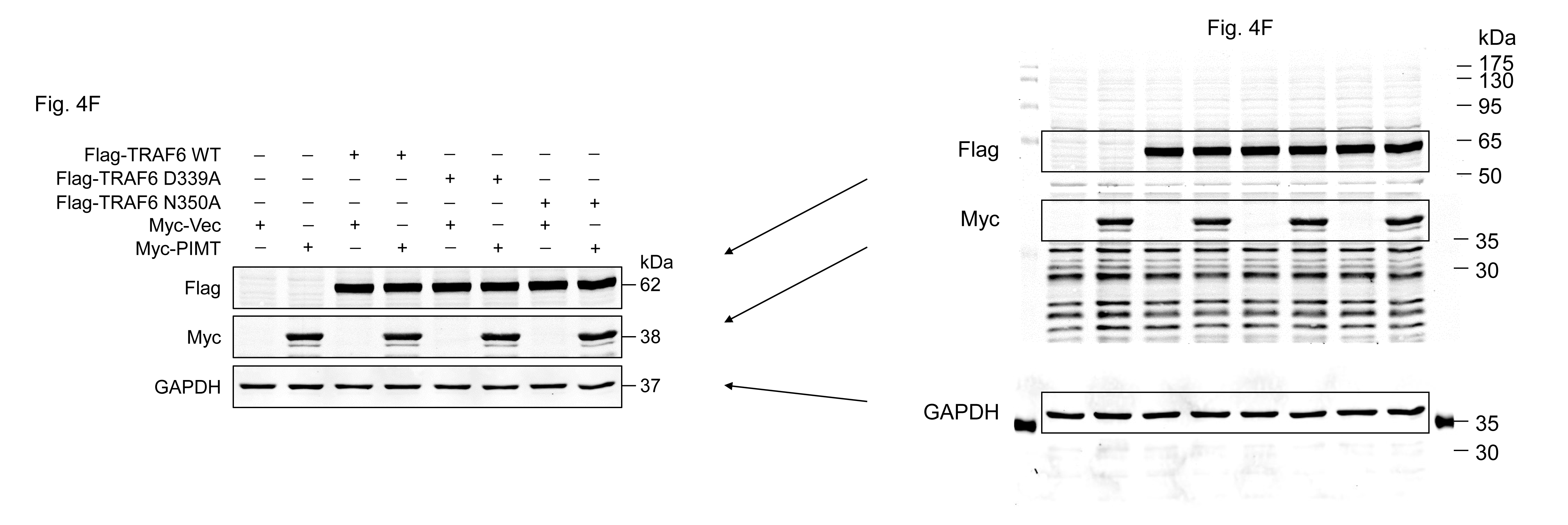

Supplement: Figure 4—source data 4. [file elife-85754-fig4-data4.zip › Figure 4- souce data 4/Figure 4F.tif]

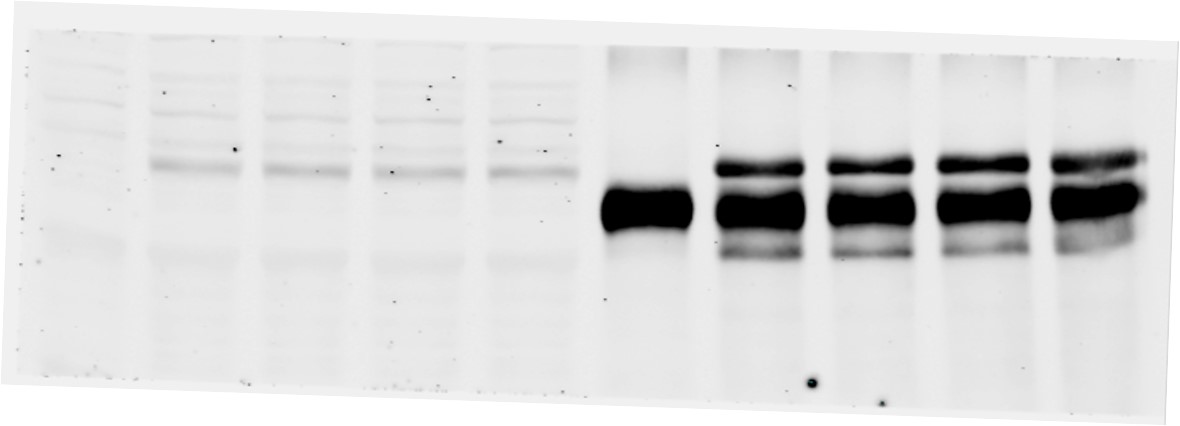

Supplement: Figure 4—source data 5. [file elife-85754-fig4-data5.zip › Figure 4- souce data 5/Fig 4G IP Flag.jpg]

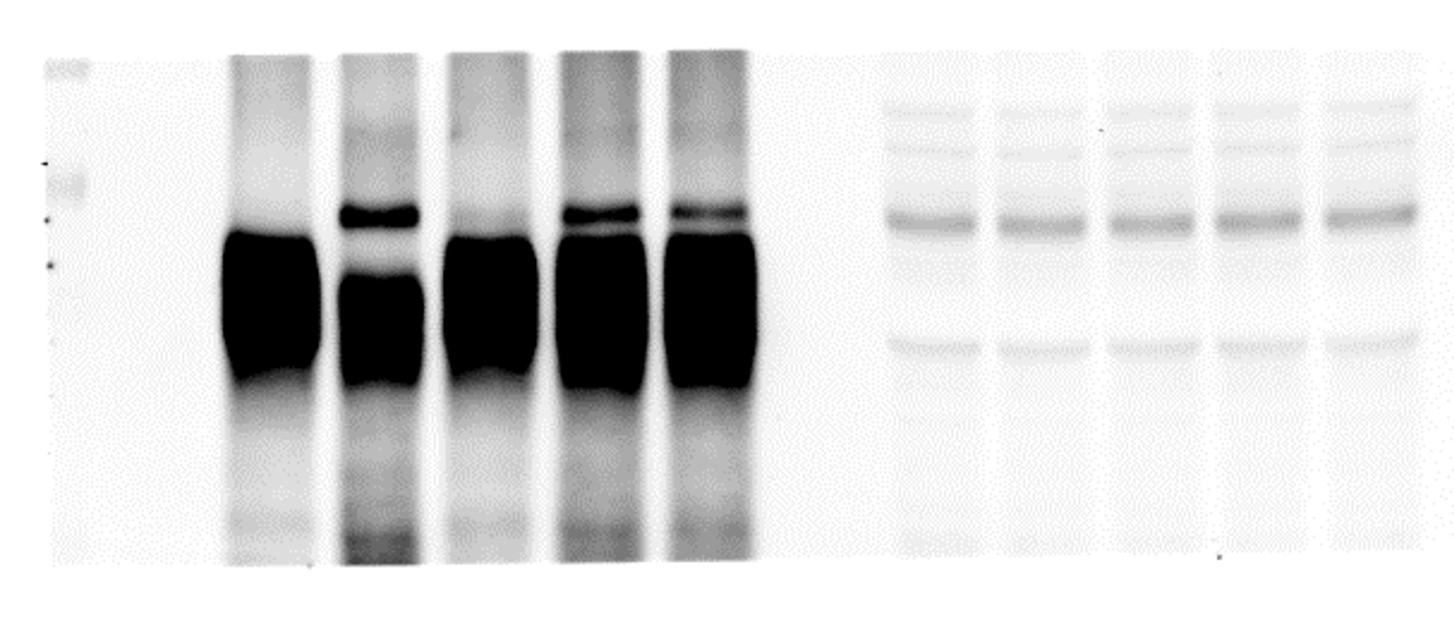

Supplement: Figure 4—source data 5. [file elife-85754-fig4-data5.zip › Figure 4- souce data 5/Fig 4G IP HA.jpg]

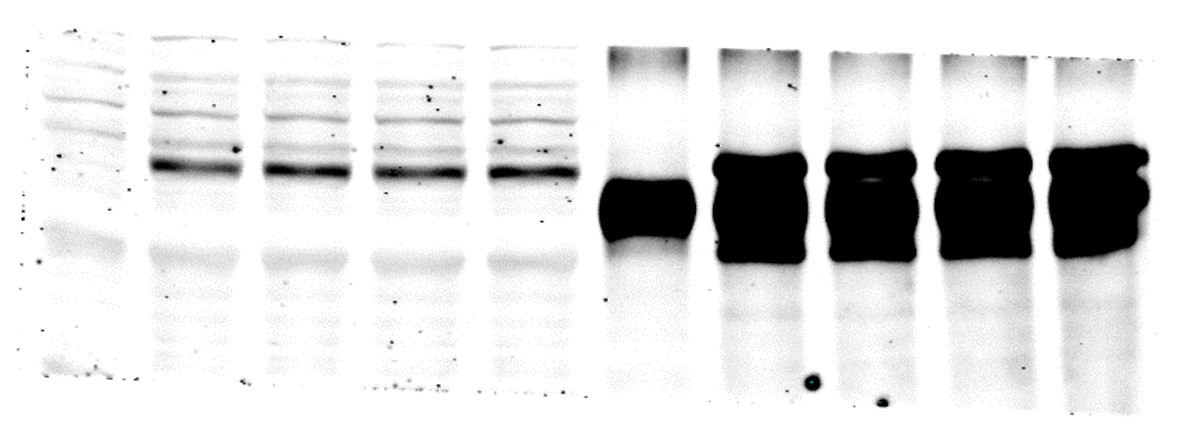

Supplement: Figure 4—source data 5. [file elife-85754-fig4-data5.zip › Figure 4- souce data 5/Fig 4G lysate Flag.jpg]

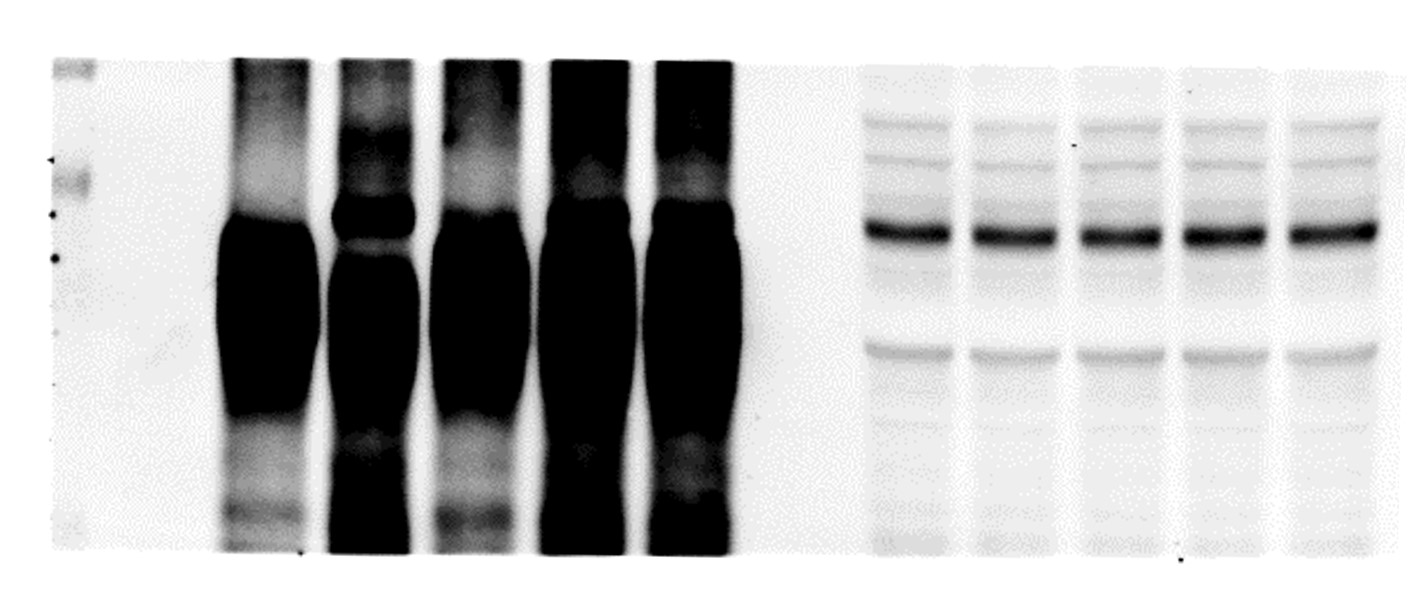

Supplement: Figure 4—source data 5. [file elife-85754-fig4-data5.zip › Figure 4- souce data 5/Fig 4G lysate HA.jpg]

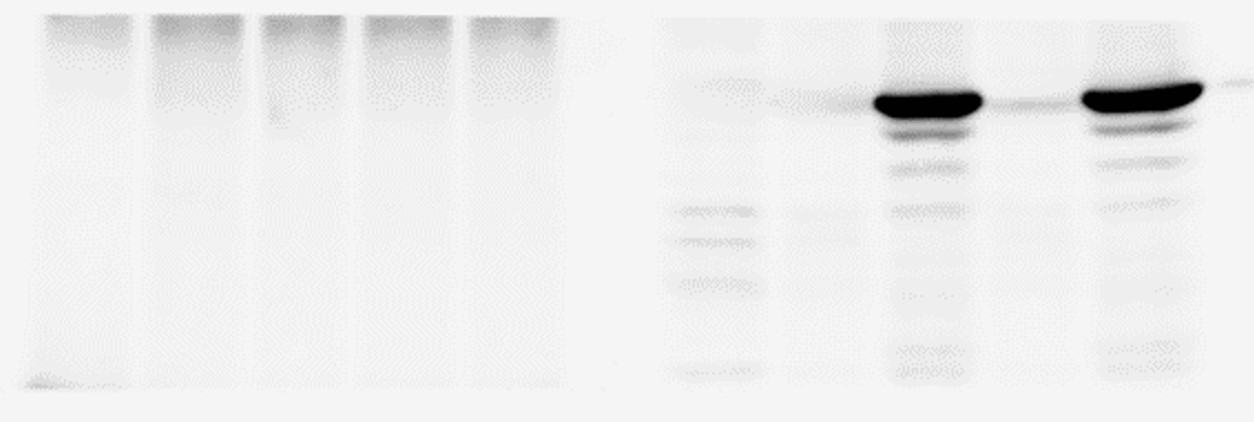

Supplement: Figure 4—source data 5. [file elife-85754-fig4-data5.zip › Figure 4- souce data 5/Fig 4G lysate Myc.jpg]

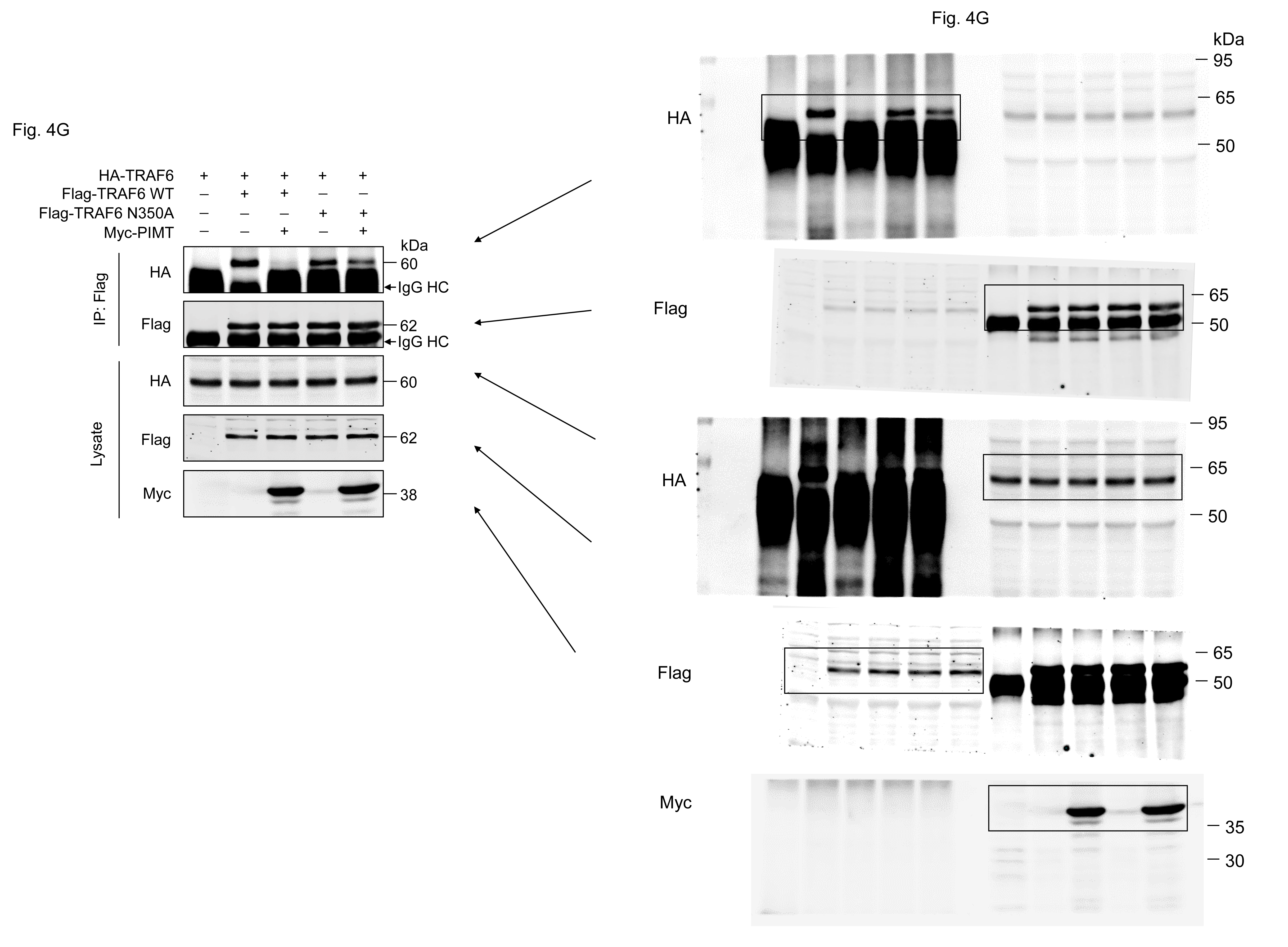

Supplement: Figure 4—source data 5. [file elife-85754-fig4-data5.zip › Figure 4- souce data 5/Figure 4G.tif]

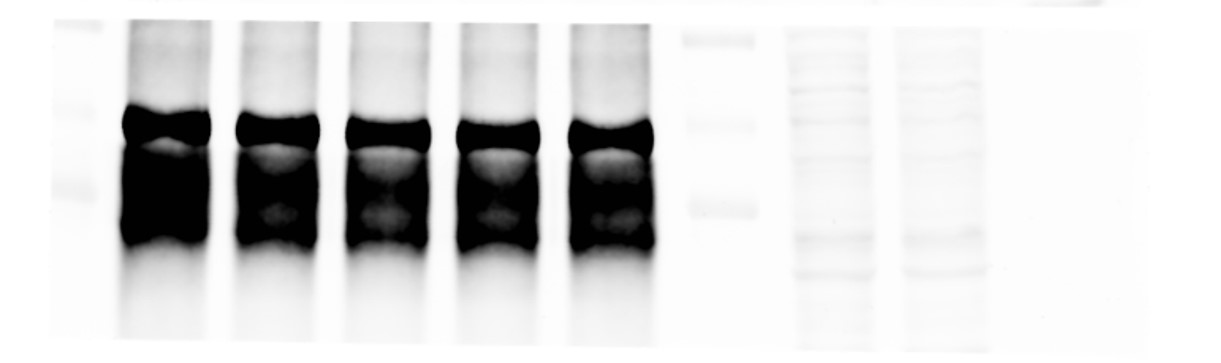

Supplement: Figure 4—source data 6. [file elife-85754-fig4-data6.zip › Figure 4- souce data 6/Fig 4H IP Flag.jpg]

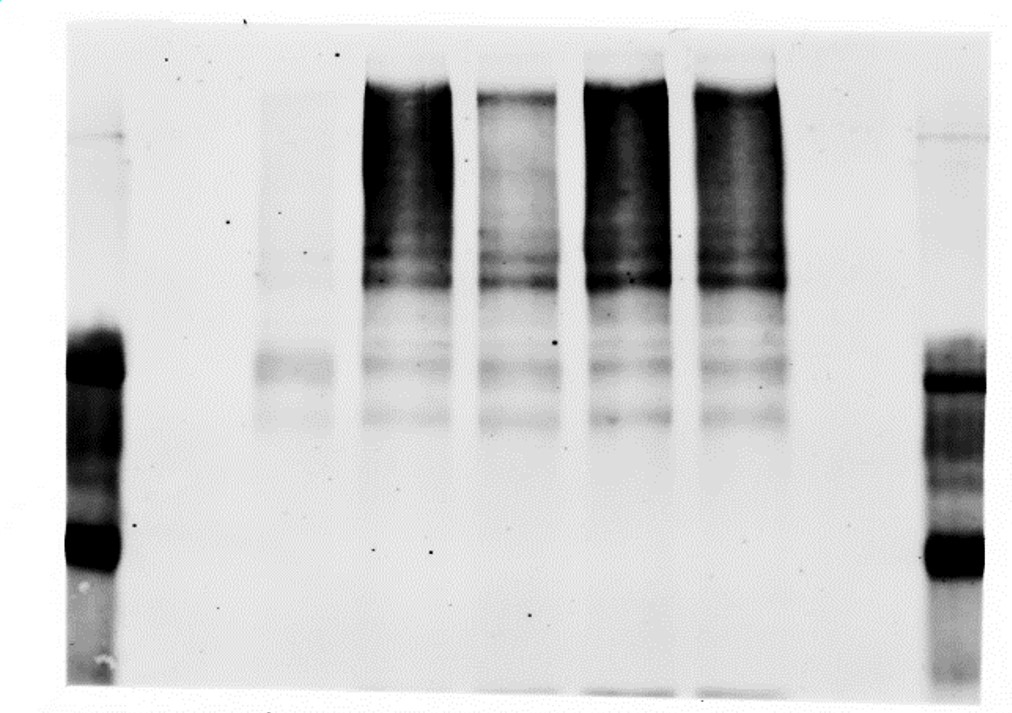

Supplement: Figure 4—source data 6. [file elife-85754-fig4-data6.zip › Figure 4- souce data 6/Fig 4H IP HA.jpg]
