# Supplementary figures and images for: PIMT is a novel and potent suppressor of endothelial activation (part 2 of 2)
Source: eLife. 2023 Apr 18;12:e85754. doi: 10.7554/eLife.85754 (PMC10112892; doi:10.7554/eLife.85754)

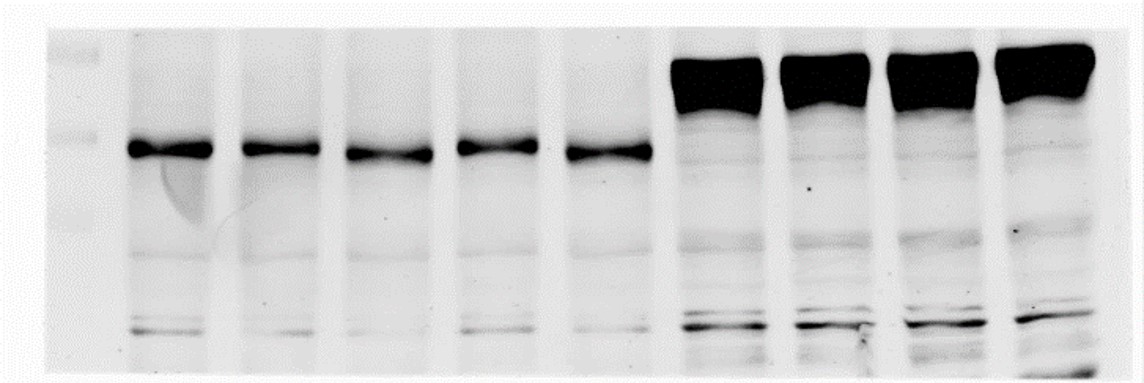

Supplement: Figure 4—source data 6. [file elife-85754-fig4-data6.zip › Figure 4- souce data 6/Fig 4H lysate Flag.jpg]

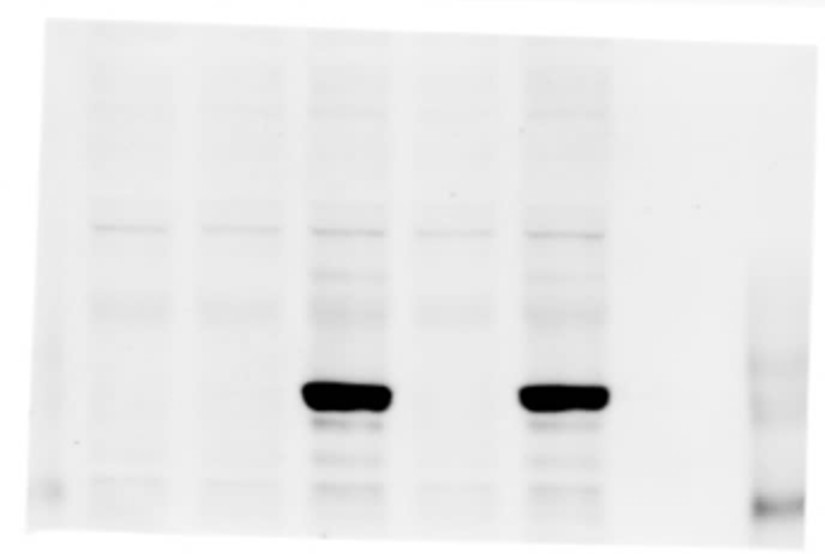

Supplement: Figure 4—source data 6. [file elife-85754-fig4-data6.zip › Figure 4- souce data 6/Fig 4H lysate Myc.jpg]

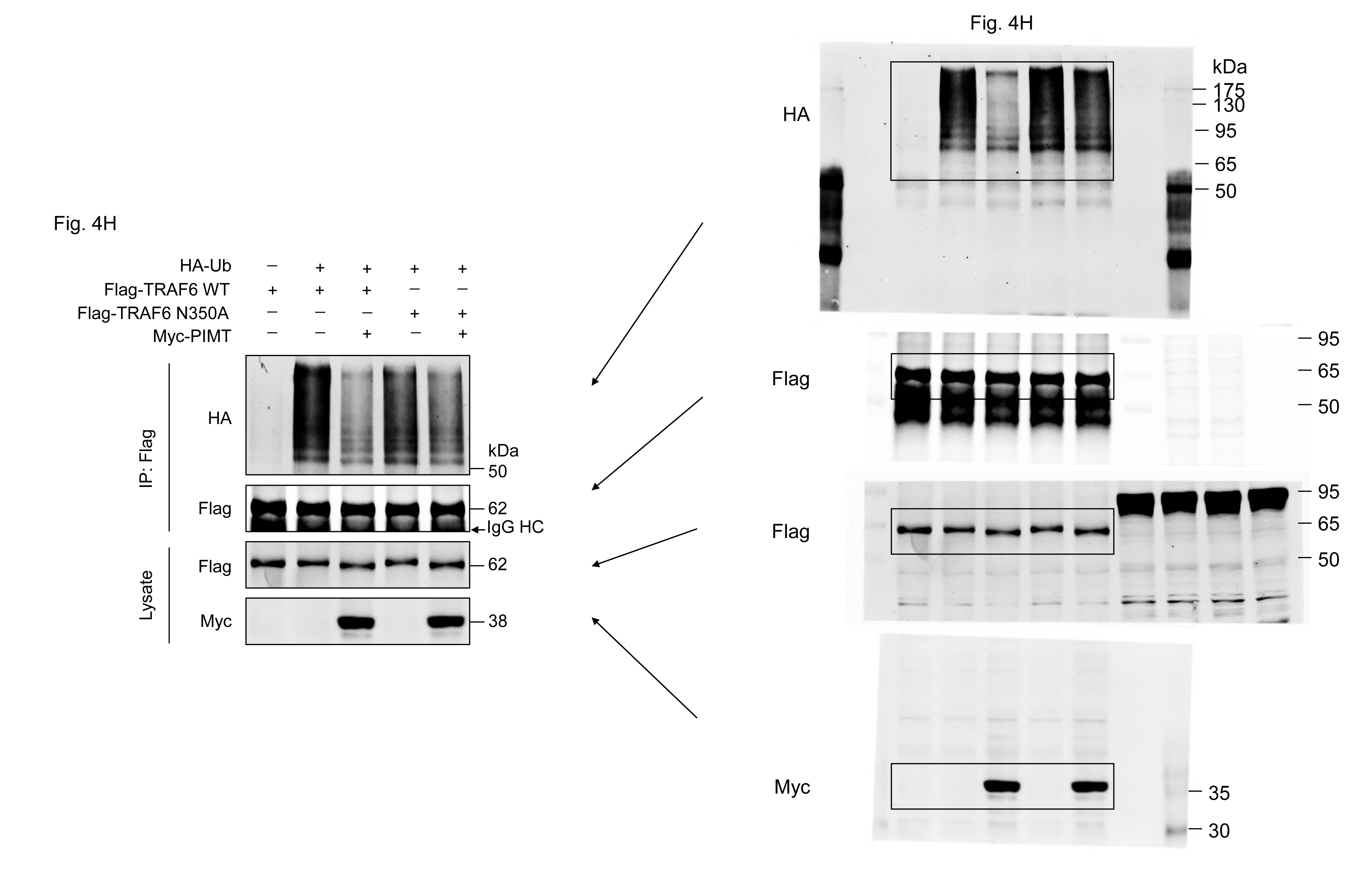

Supplement: Figure 4—source data 6. [file elife-85754-fig4-data6.zip › Figure 4- souce data 6/Figure 4H.tif]

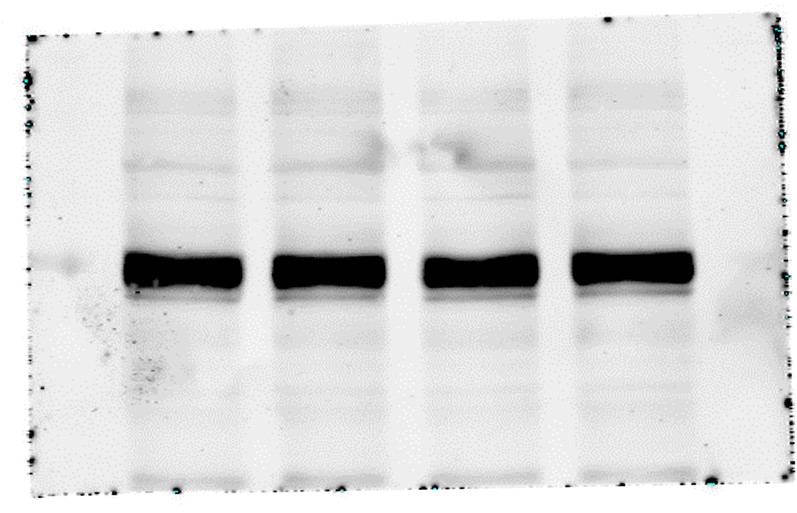

Supplement: Figure 4—figure supplement 1—source data 1. [file elife-85754-fig4-figsupp1-data1.zip › Figure 4-figure supplement 1-source data 1/Fig 4 S1A IP Flag.jpg]

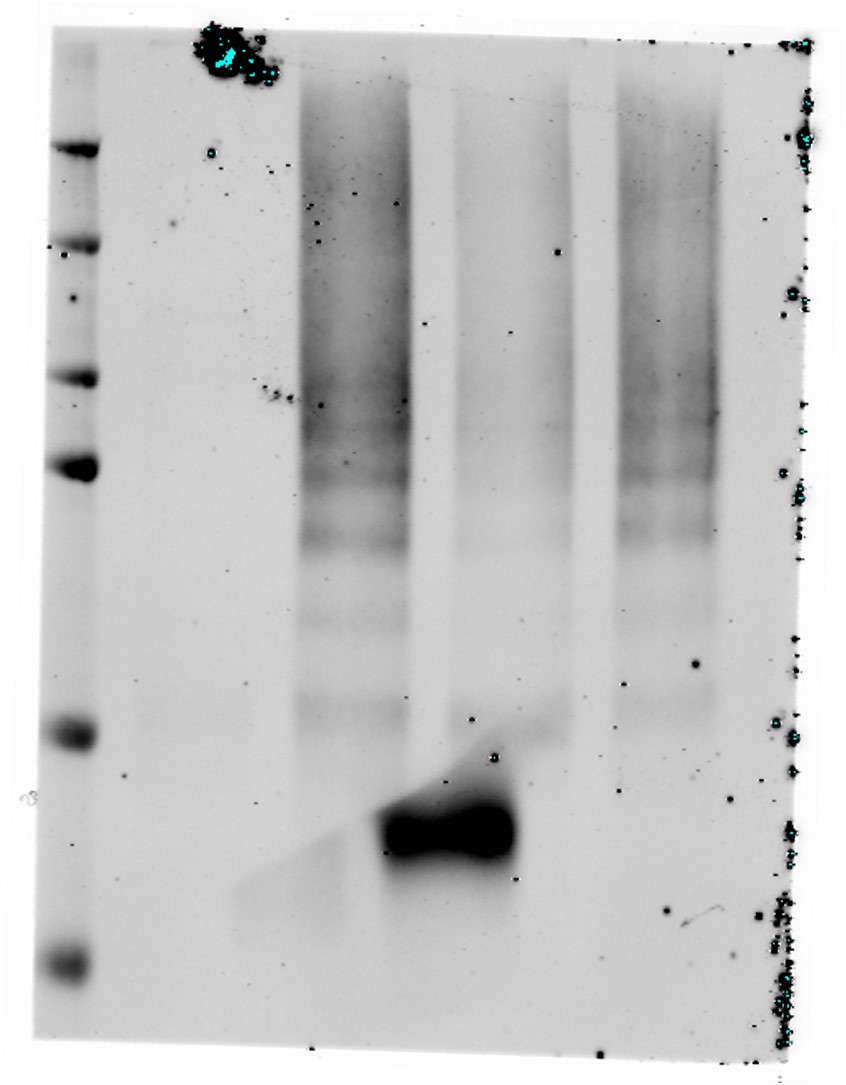

Supplement: Figure 4—figure supplement 1—source data 1. [file elife-85754-fig4-figsupp1-data1.zip › Figure 4-figure supplement 1-source data 1/Fig 4 S1A IP HA.jpg]

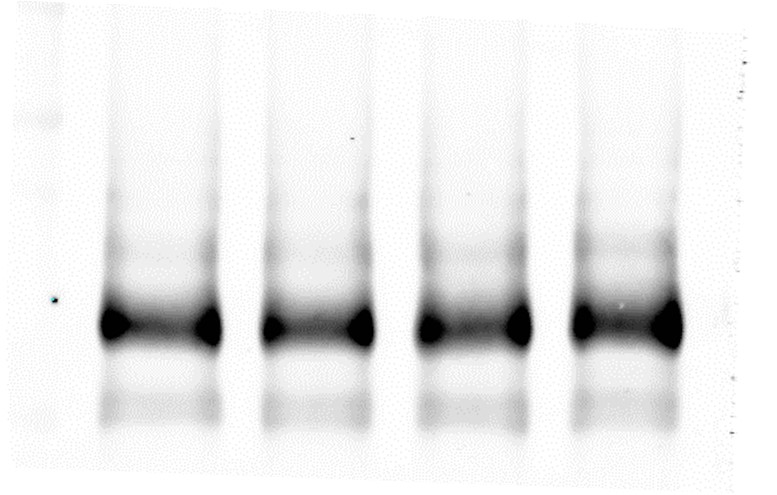

Supplement: Figure 4—figure supplement 1—source data 1. [file elife-85754-fig4-figsupp1-data1.zip › Figure 4-figure supplement 1-source data 1/Fig 4 S1A lysate Flag.jpg]

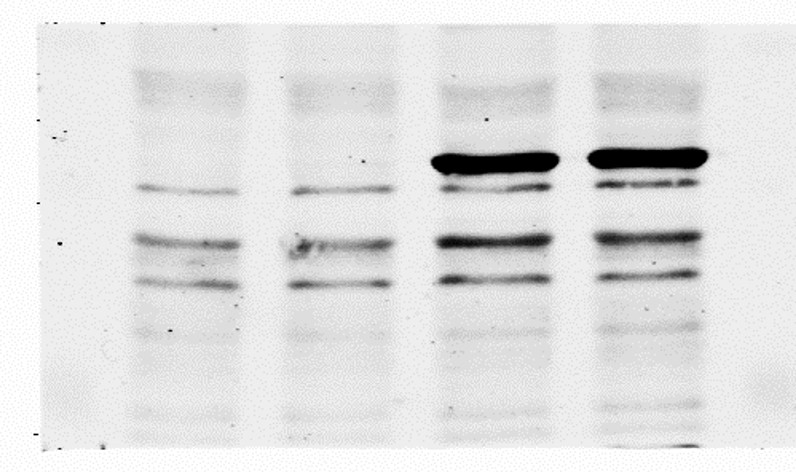

Supplement: Figure 4—figure supplement 1—source data 1. [file elife-85754-fig4-figsupp1-data1.zip › Figure 4-figure supplement 1-source data 1/Fig 4 S1A lysate Myc.jpg]

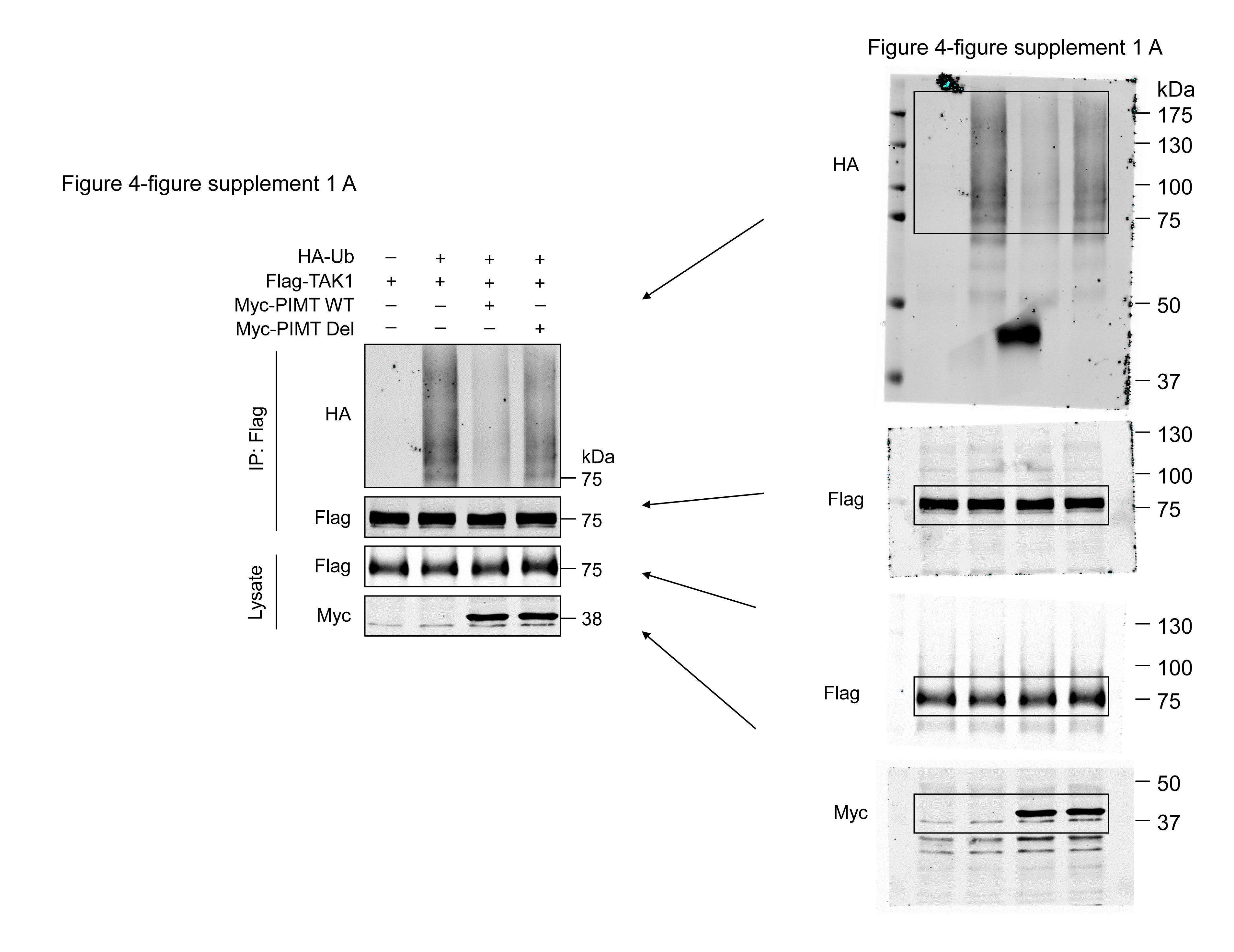

Supplement: Figure 4—figure supplement 1—source data 1. [file elife-85754-fig4-figsupp1-data1.zip › Figure 4-figure supplement 1-source data 1/Figure 4-figure supplement 1A.tif]

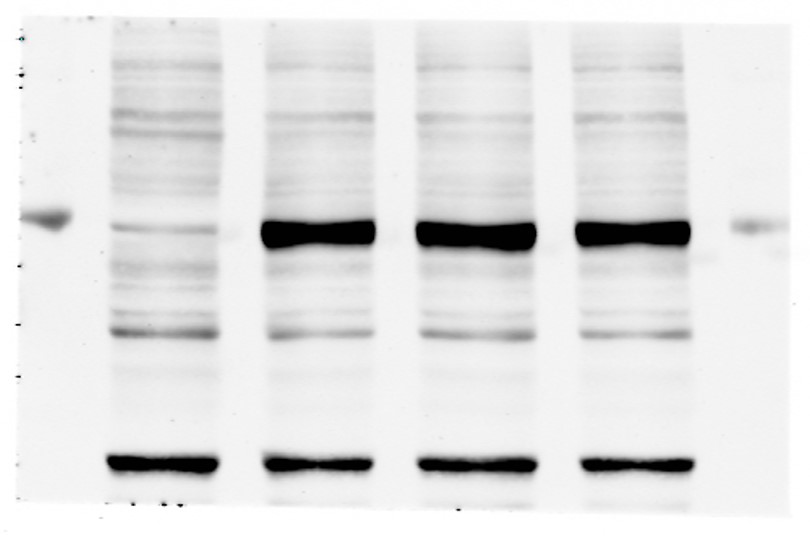

Supplement: Figure 4—figure supplement 1—source data 2. [file elife-85754-fig4-figsupp1-data2.zip › Figure 4-figure supplement 1-source data 2/Fig 4 S1B Flag.jpg]

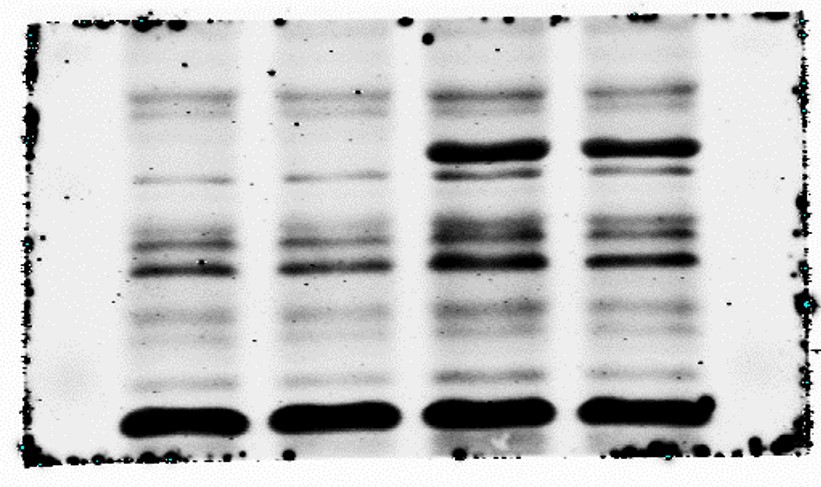

Supplement: Figure 4—figure supplement 1—source data 2. [file elife-85754-fig4-figsupp1-data2.zip › Figure 4-figure supplement 1-source data 2/Fig 4 S1B Myc.jpg]

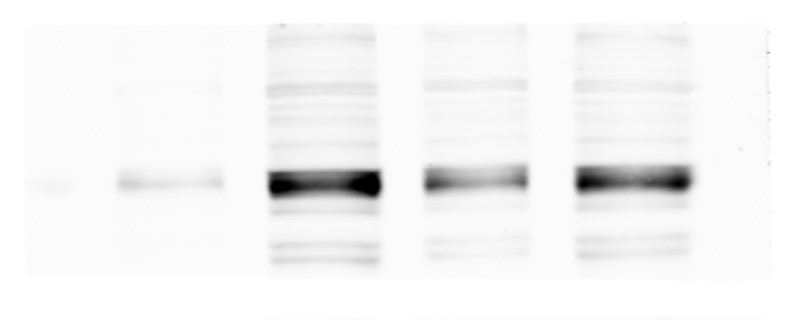

Supplement: Figure 4—figure supplement 1—source data 2. [file elife-85754-fig4-figsupp1-data2.zip › Figure 4-figure supplement 1-source data 2/Fig 4 S1B P-TAK1.jpg]

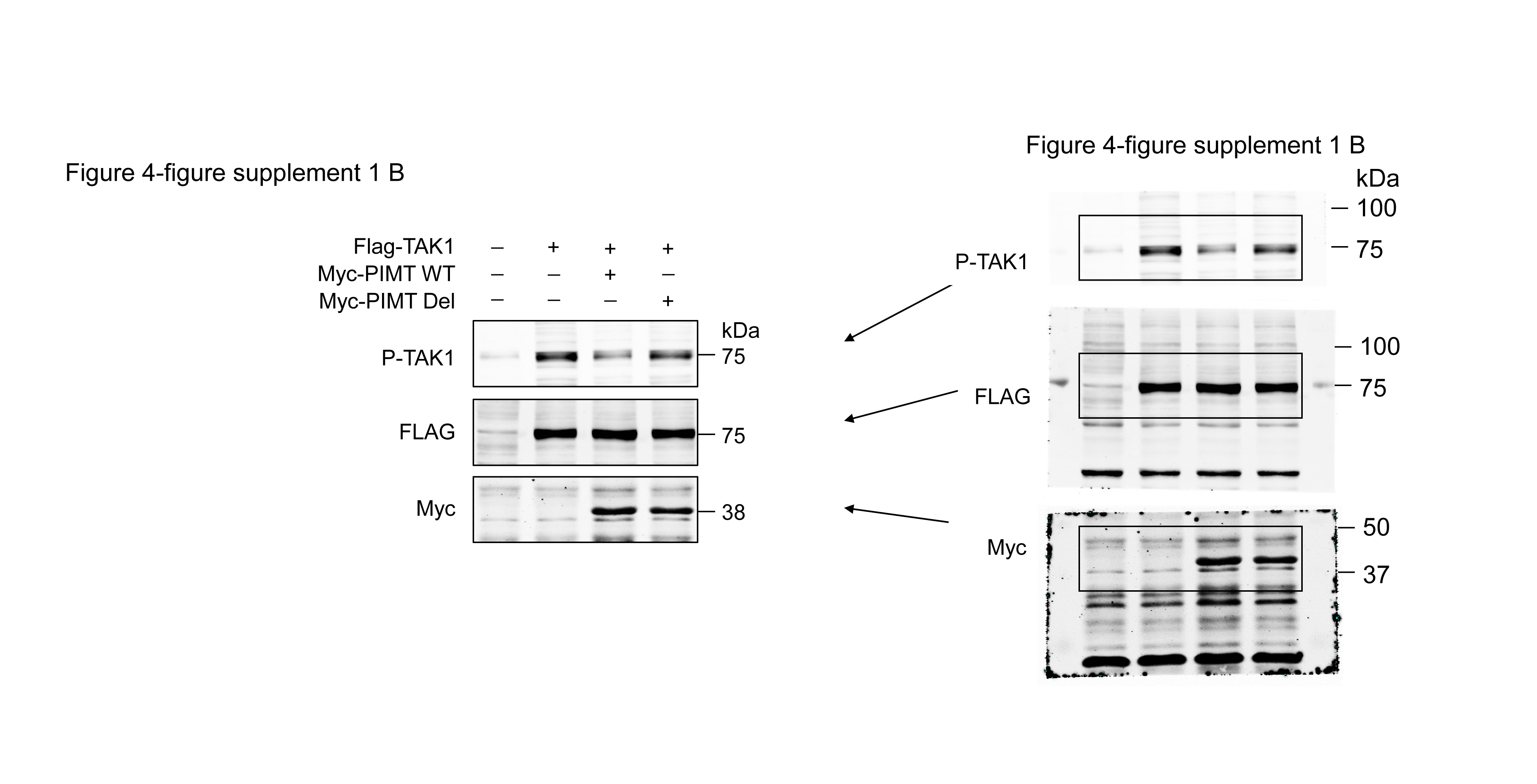

Supplement: Figure 4—figure supplement 1—source data 2. [file elife-85754-fig4-figsupp1-data2.zip › Figure 4-figure supplement 1-source data 2/Figure 4-figure supplement 1B.tif]

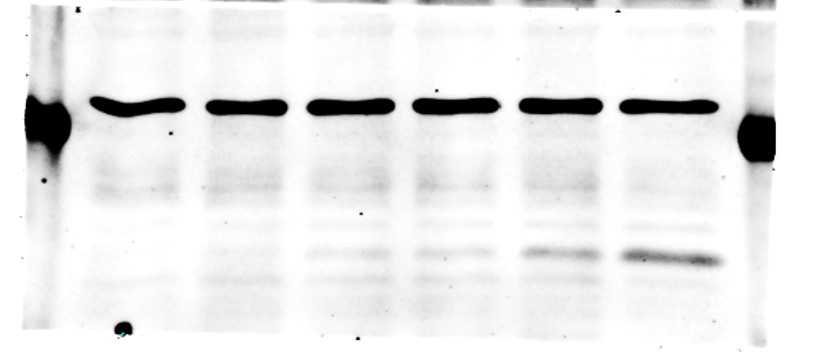

Supplement: Figure 5—source data 1. [file elife-85754-fig5-data1.zip › Figure 5- souce data 1/Fig 5A GAPDH.jpg]

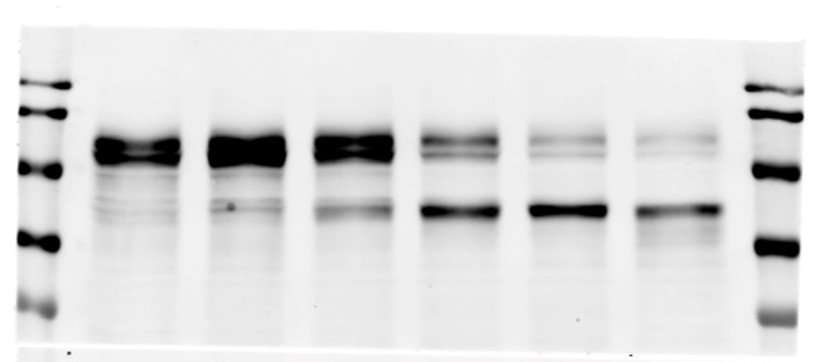

Supplement: Figure 5—source data 1. [file elife-85754-fig5-data1.zip › Figure 5- souce data 1/Fig 5A ICAM-1.jpg]

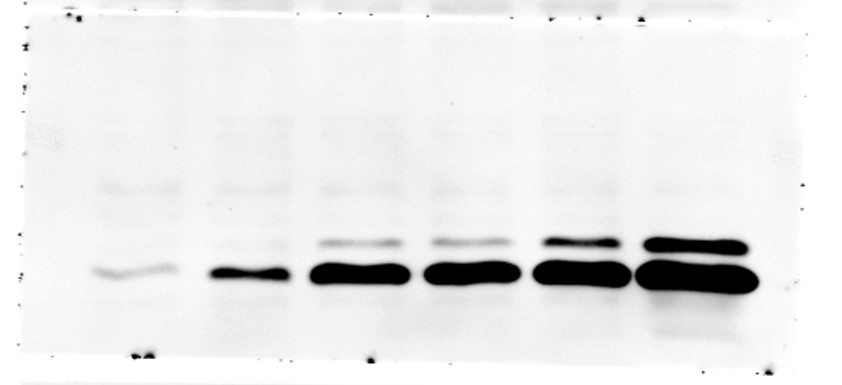

Supplement: Figure 5—source data 1. [file elife-85754-fig5-data1.zip › Figure 5- souce data 1/Fig 5A PIMT.jpg]

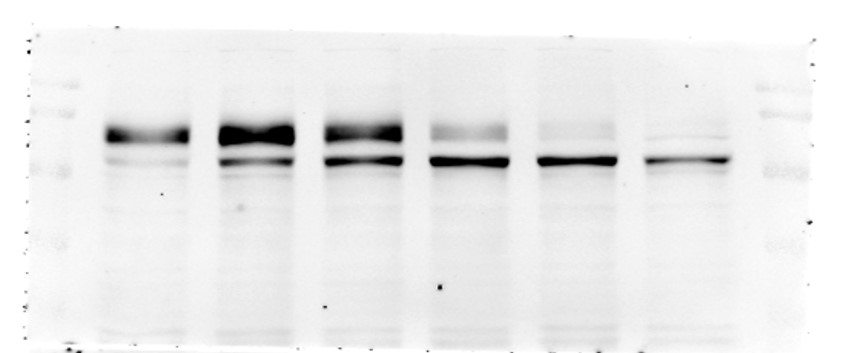

Supplement: Figure 5—source data 1. [file elife-85754-fig5-data1.zip › Figure 5- souce data 1/Fig 5A VCAM-1.jpg]

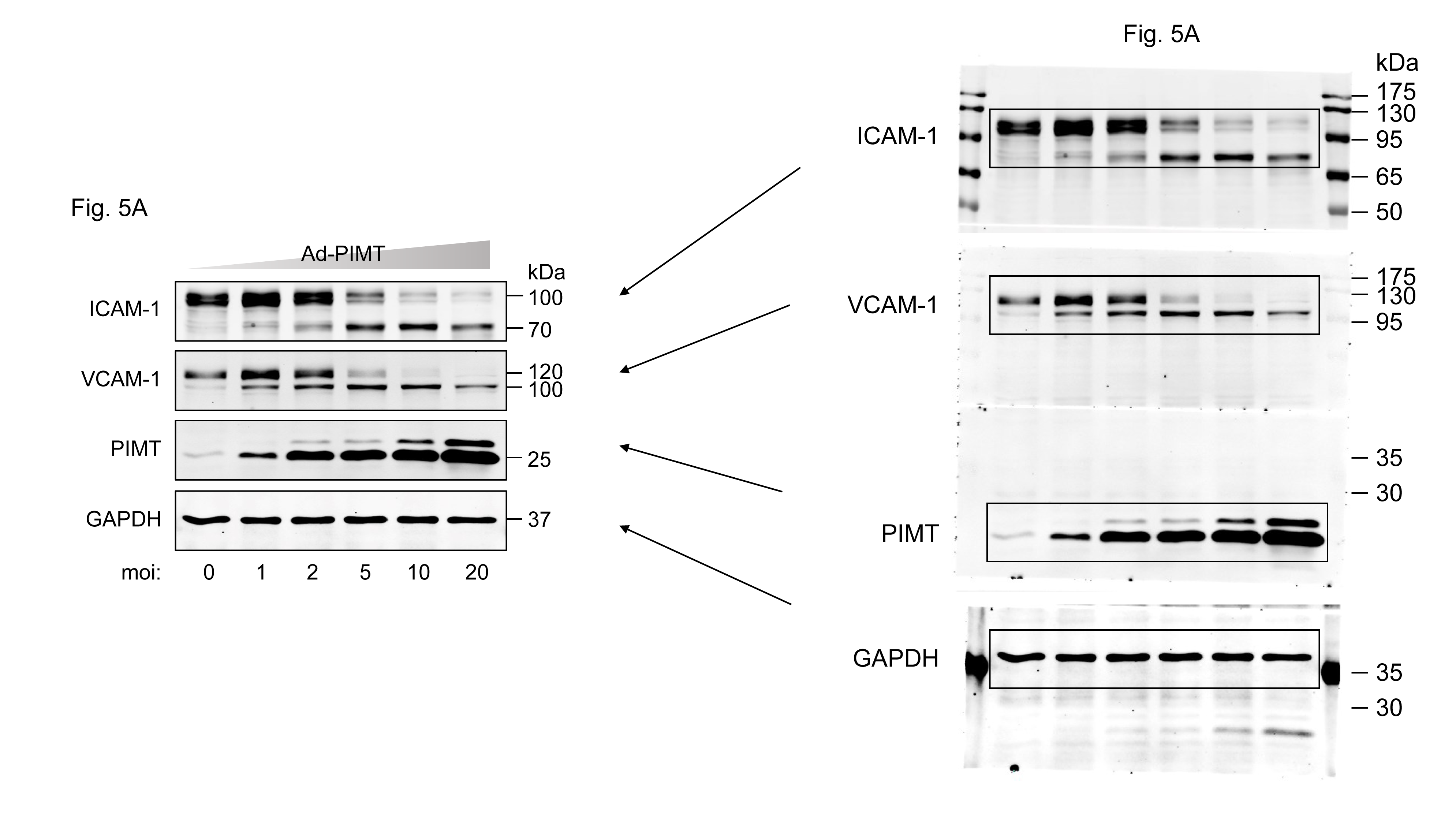

Supplement: Figure 5—source data 1. [file elife-85754-fig5-data1.zip › Figure 5- souce data 1/Figure 5A.tif]

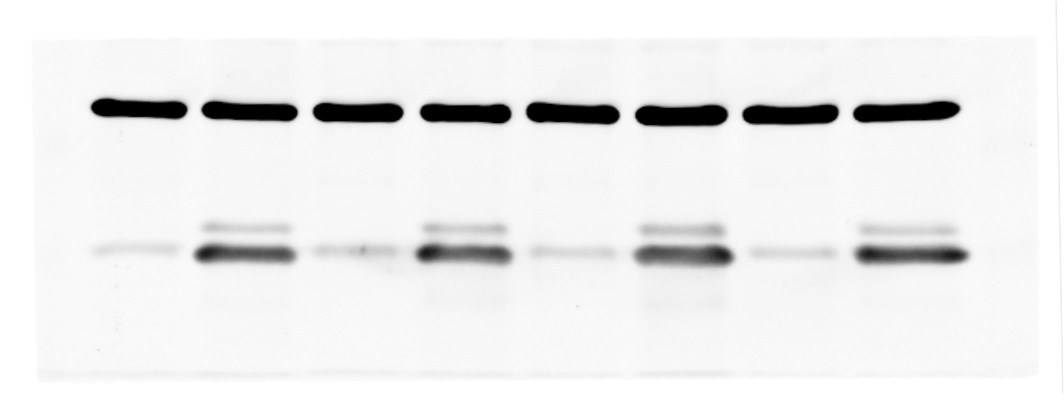

Supplement: Figure 5—source data 2. [file elife-85754-fig5-data2.zip › Figure 5- souce data 2/Fig 5B GAPDH.jpg]

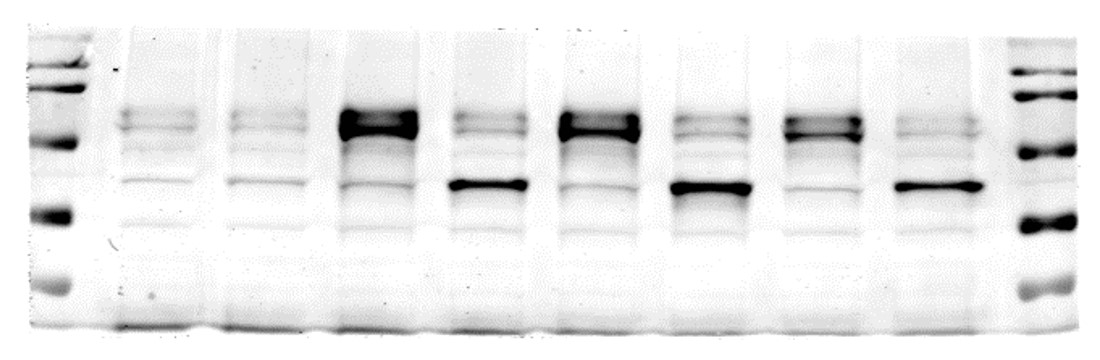

Supplement: Figure 5—source data 2. [file elife-85754-fig5-data2.zip › Figure 5- souce data 2/Fig 5B ICAM-1.jpg]

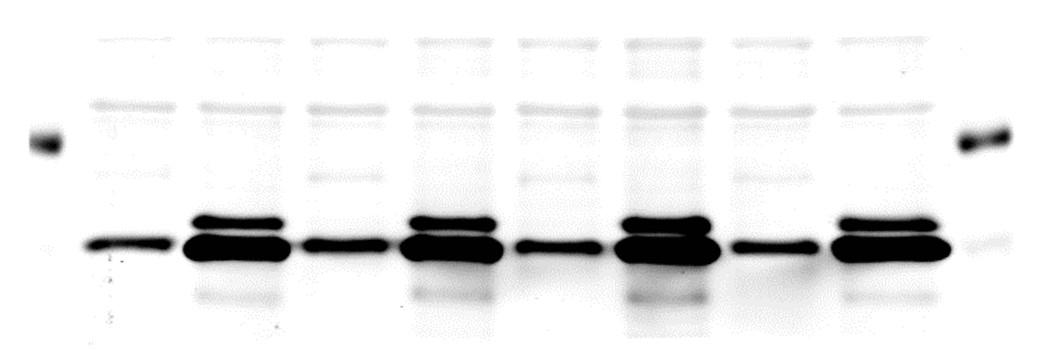

Supplement: Figure 5—source data 2. [file elife-85754-fig5-data2.zip › Figure 5- souce data 2/Fig 5B PIMT.jpg]

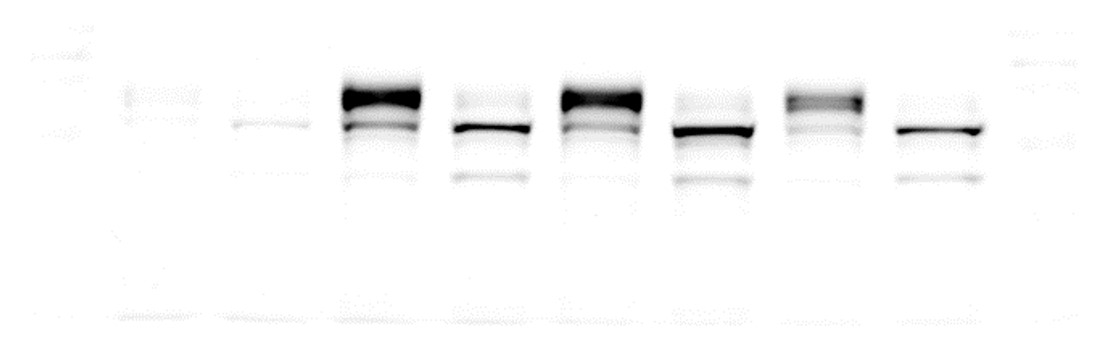

Supplement: Figure 5—source data 2. [file elife-85754-fig5-data2.zip › Figure 5- souce data 2/Fig 5B VCAM-1.jpg]

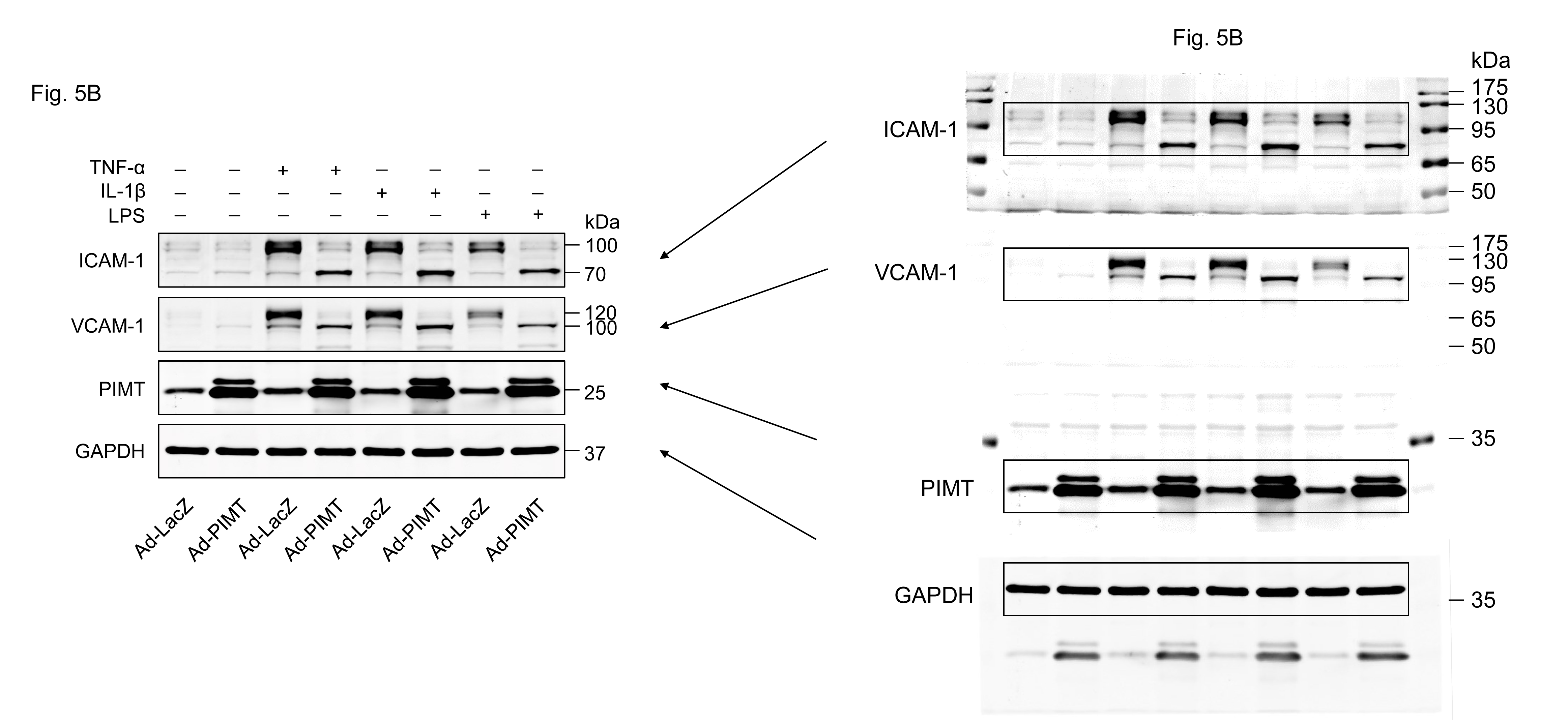

Supplement: Figure 5—source data 2. [file elife-85754-fig5-data2.zip › Figure 5- souce data 2/Figure 5B.tif]

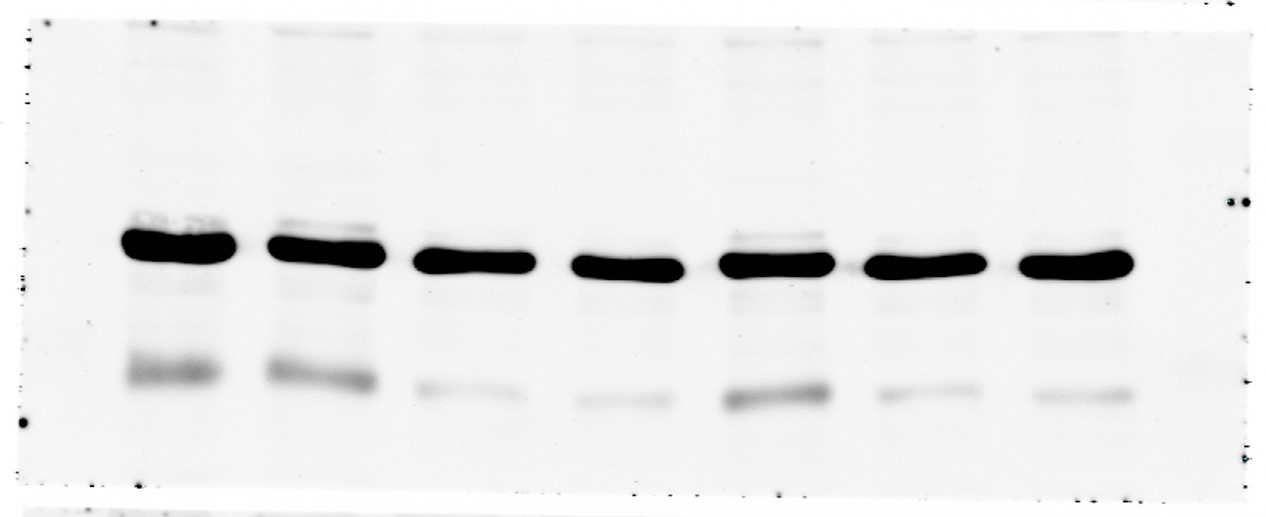

Supplement: Figure 5—source data 3. [file elife-85754-fig5-data3.zip › Figure 5- souce data 3/Fig 5C GAPDH.jpg]

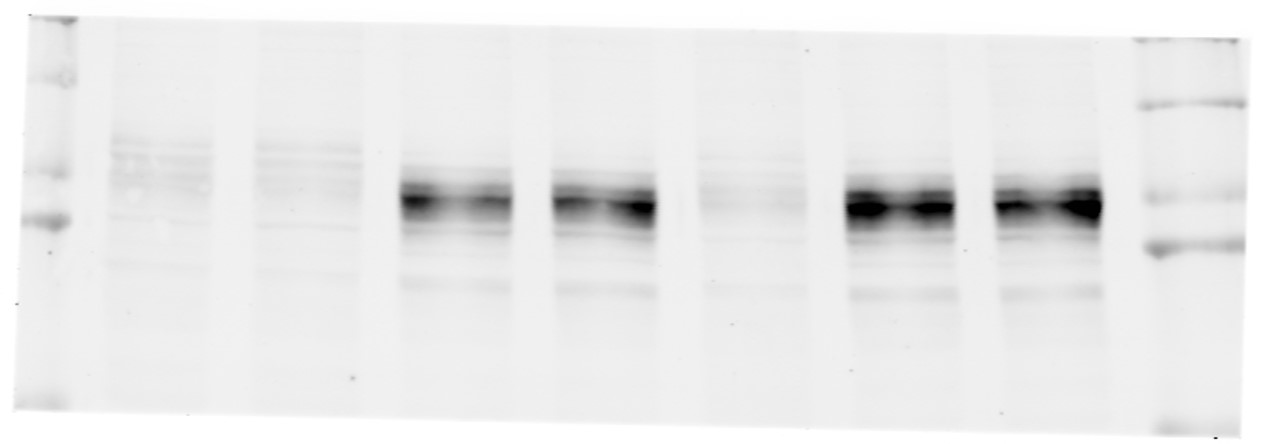

Supplement: Figure 5—source data 3. [file elife-85754-fig5-data3.zip › Figure 5- souce data 3/Fig 5C ICAM-1.jpg]

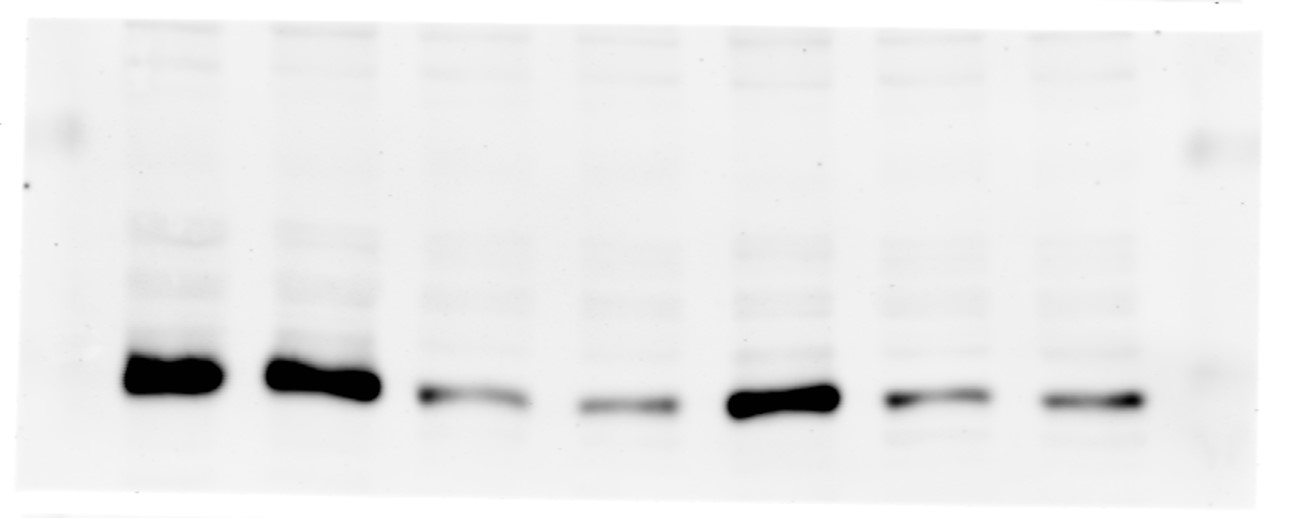

Supplement: Figure 5—source data 3. [file elife-85754-fig5-data3.zip › Figure 5- souce data 3/Fig 5C PIMT.jpg]

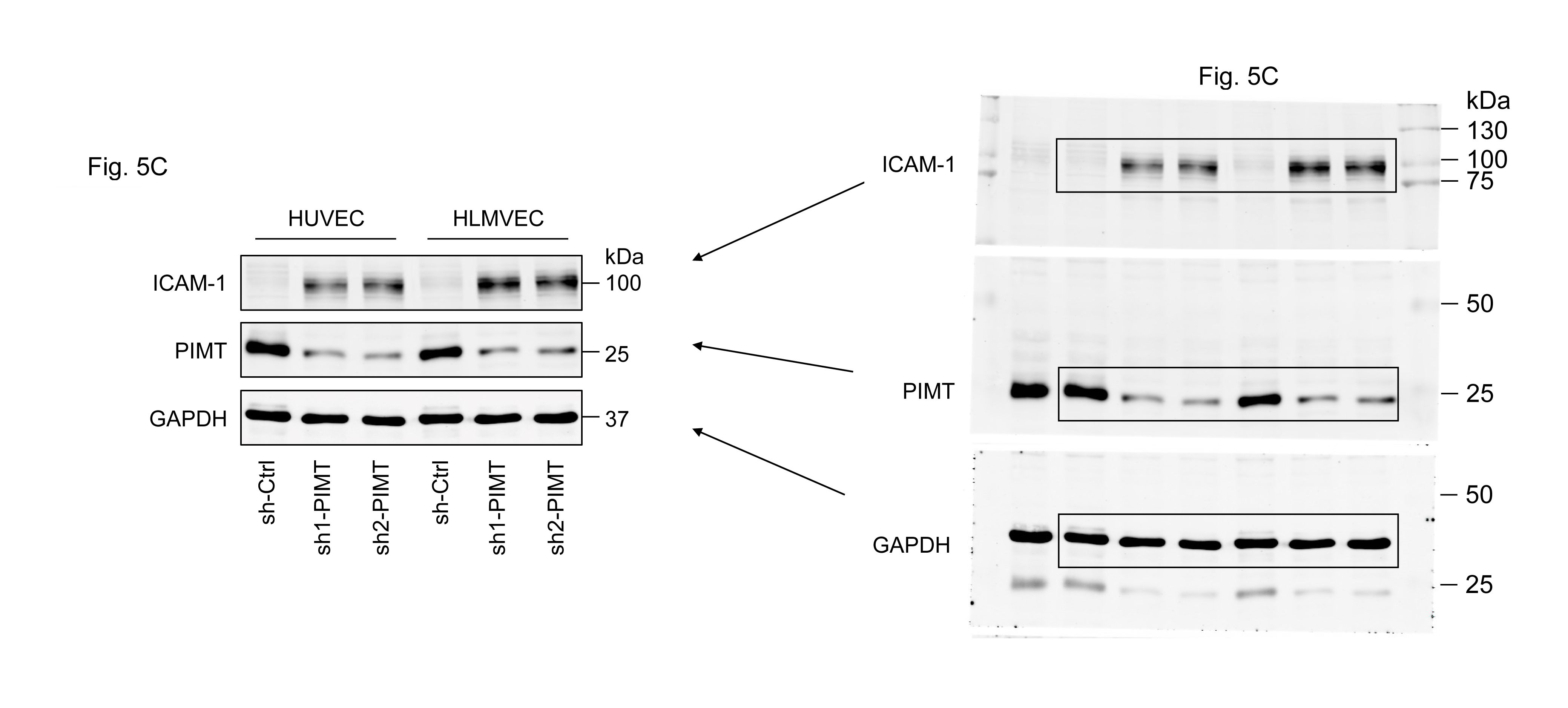

Supplement: Figure 5—source data 3. [file elife-85754-fig5-data3.zip › Figure 5- souce data 3/Figure 5C.tif]

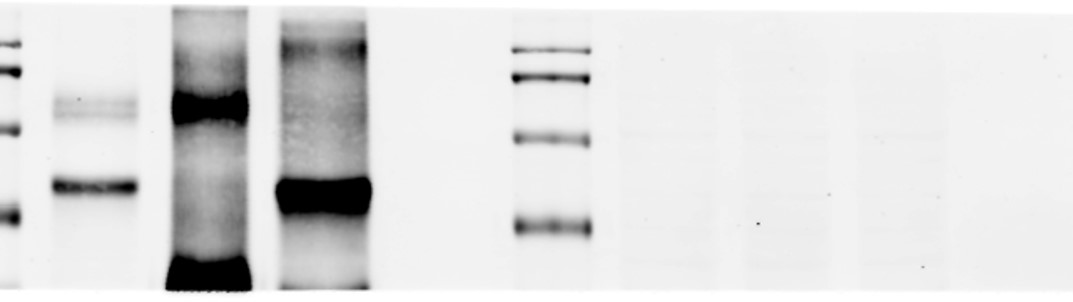

Supplement: Figure 5—source data 4. [file elife-85754-fig5-data4.zip › Figure 5- souce data 4/Fig 5D ICAM-1.jpg]

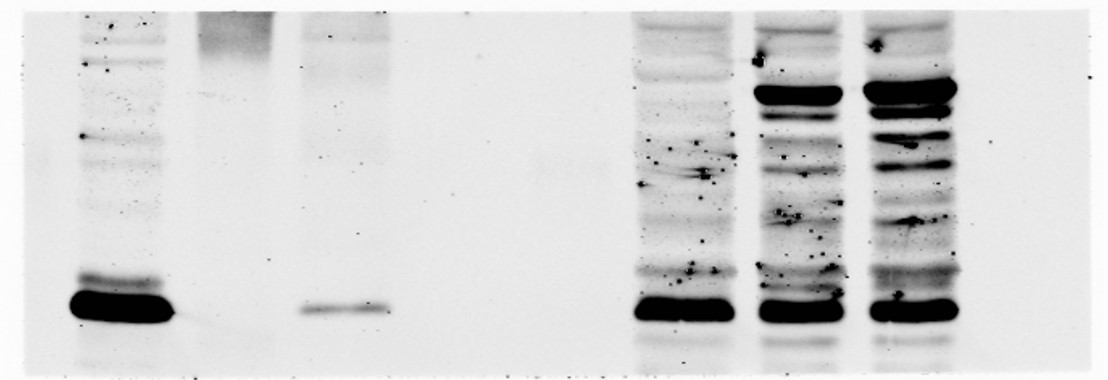

Supplement: Figure 5—source data 4. [file elife-85754-fig5-data4.zip › Figure 5- souce data 4/Fig 5D PIMT.jpg]

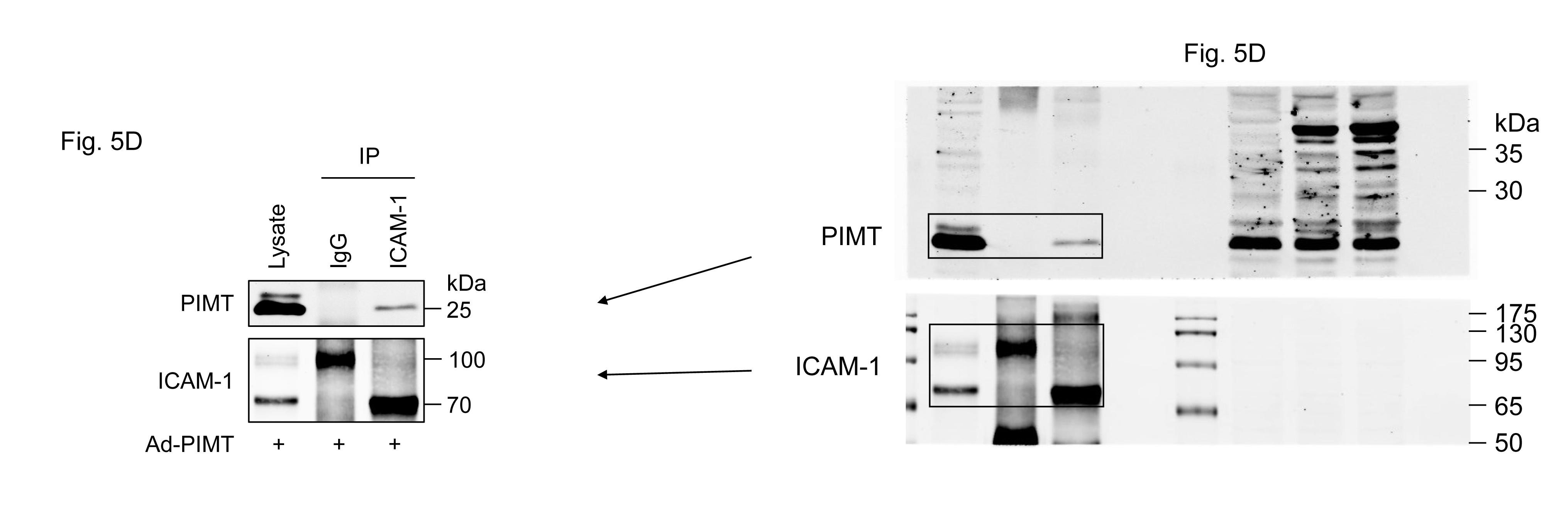

Supplement: Figure 5—source data 4. [file elife-85754-fig5-data4.zip › Figure 5- souce data 4/Figure 5D.tif]

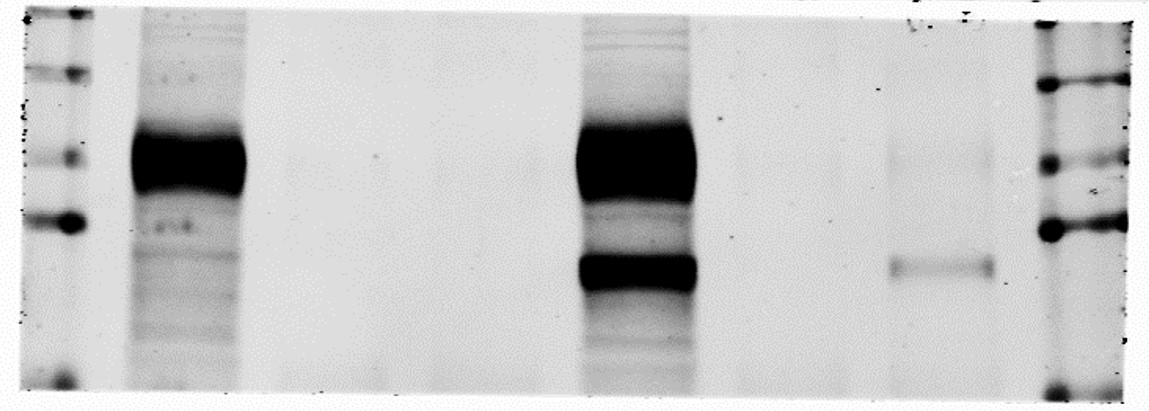

Supplement: Figure 5—source data 5. [file elife-85754-fig5-data5.zip › Figure 5- souce data 5/Fig 5E ICAM-1.jpg]

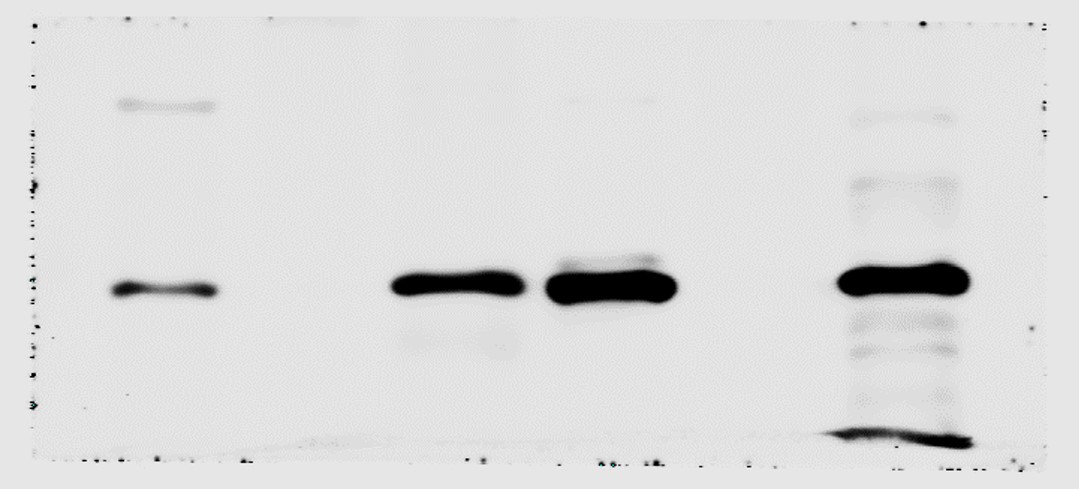

Supplement: Figure 5—source data 5. [file elife-85754-fig5-data5.zip › Figure 5- souce data 5/Fig 5E PIMT.jpg]

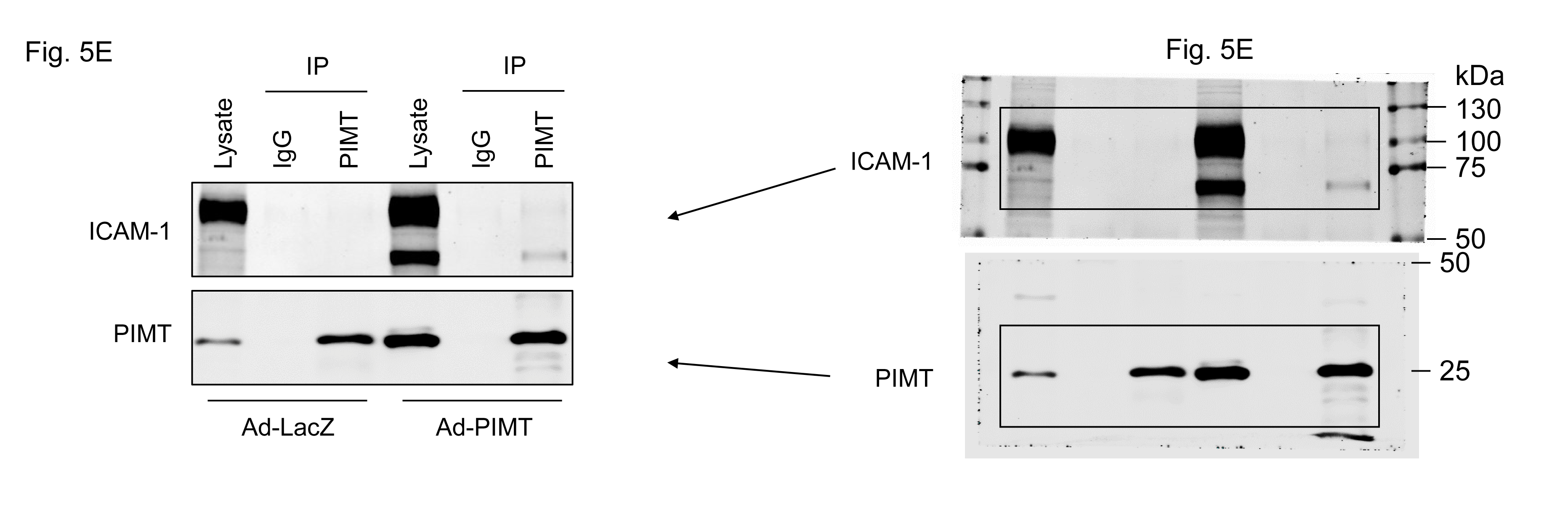

Supplement: Figure 5—source data 5. [file elife-85754-fig5-data5.zip › Figure 5- souce data 5/Figure 5E.tif]

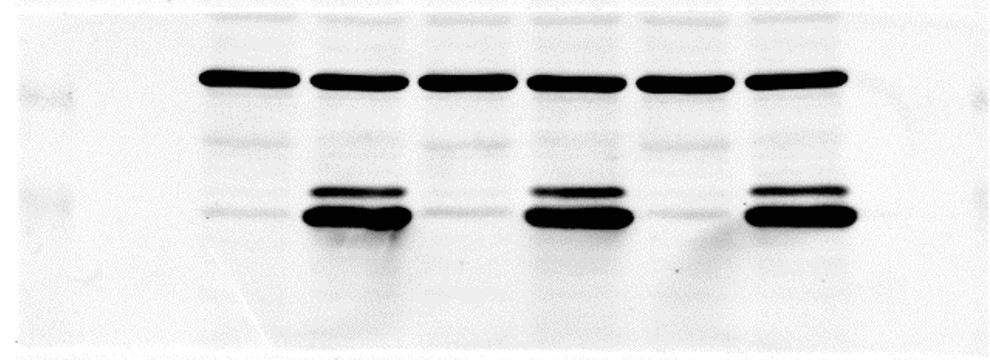

Supplement: Figure 5—figure supplement 1—source data 1. [file elife-85754-fig5-figsupp1-data1.zip › Figure 5-figure supplement 1-source data 1/Fig 5 S1A GAPDH.jpg]

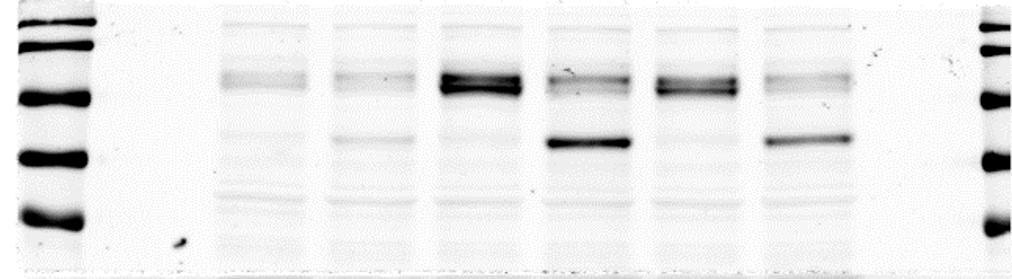

Supplement: Figure 5—figure supplement 1—source data 1. [file elife-85754-fig5-figsupp1-data1.zip › Figure 5-figure supplement 1-source data 1/Fig 5 S1A ICAM-1.jpg]

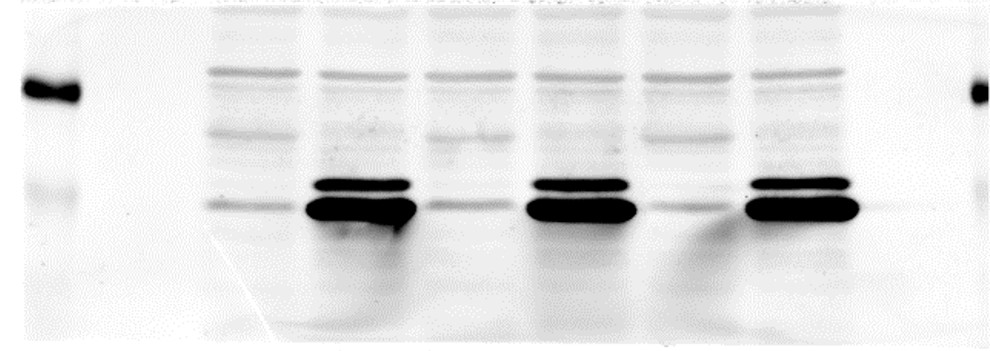

Supplement: Figure 5—figure supplement 1—source data 1. [file elife-85754-fig5-figsupp1-data1.zip › Figure 5-figure supplement 1-source data 1/Fig 5 S1A PIMT.jpg]

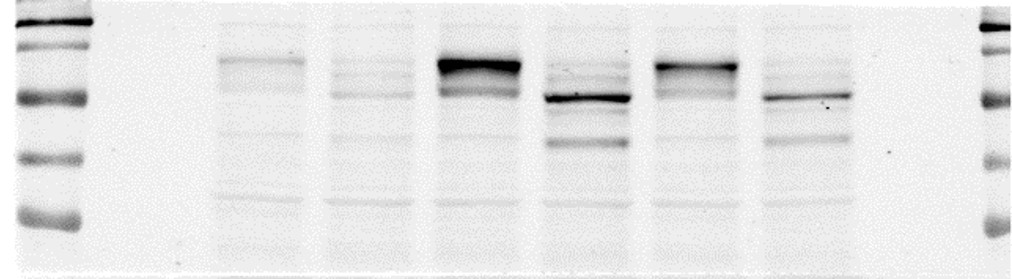

Supplement: Figure 5—figure supplement 1—source data 1. [file elife-85754-fig5-figsupp1-data1.zip › Figure 5-figure supplement 1-source data 1/Fig 5 S1A VCAM-1.jpg]

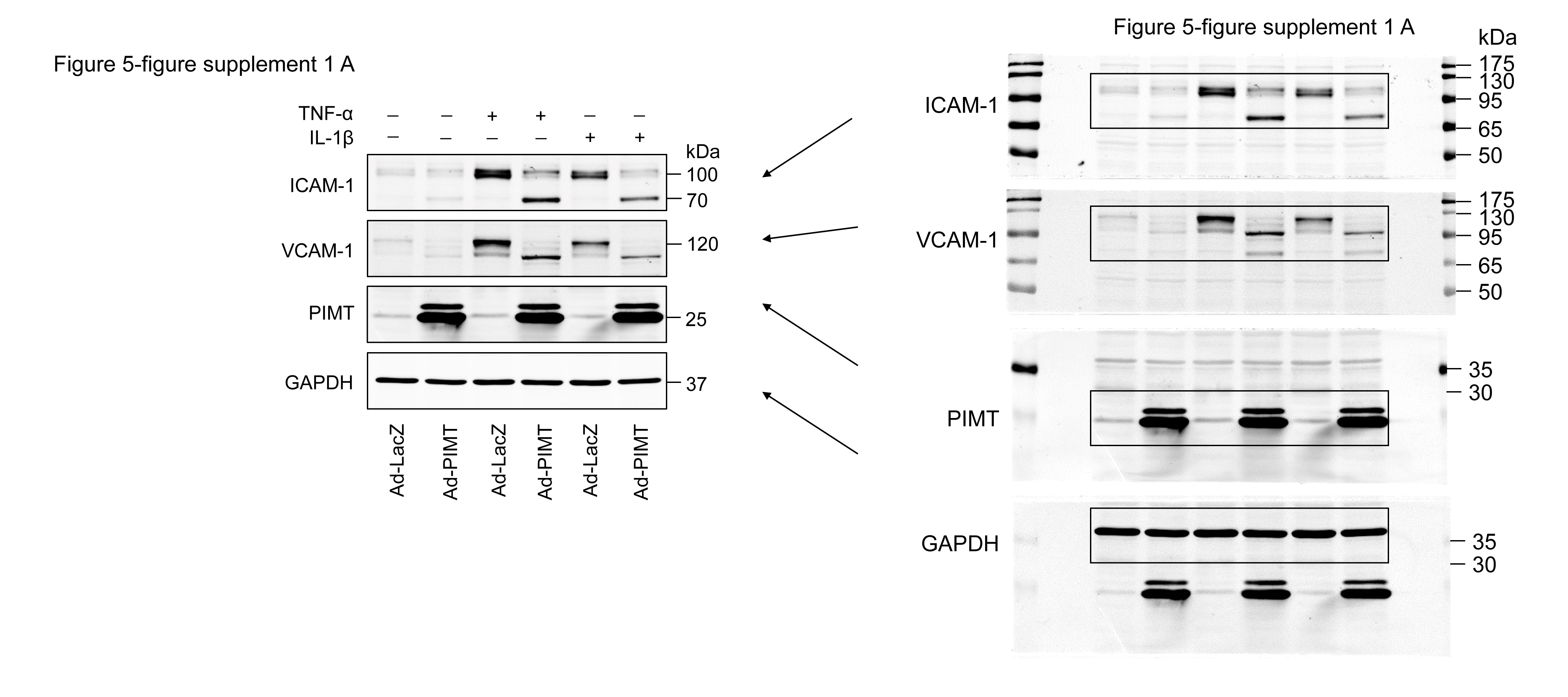

Supplement: Figure 5—figure supplement 1—source data 1. [file elife-85754-fig5-figsupp1-data1.zip › Figure 5-figure supplement 1-source data 1/Figure 5-figure supplement 1A.tif]

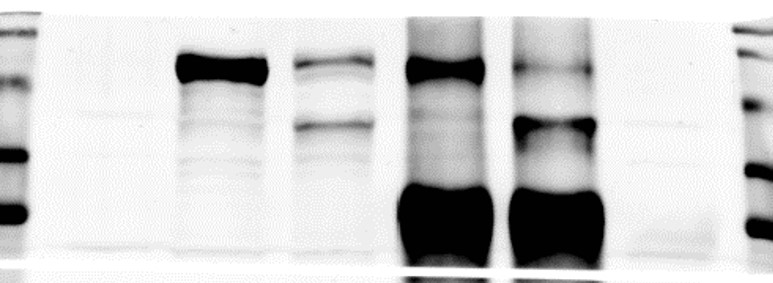

Supplement: Figure 5—figure supplement 1—source data 2. [file elife-85754-fig5-figsupp1-data2.zip › Figure 5-figure supplement 1-source data 2/Fig 5 S1B ICAM-1.jpg]

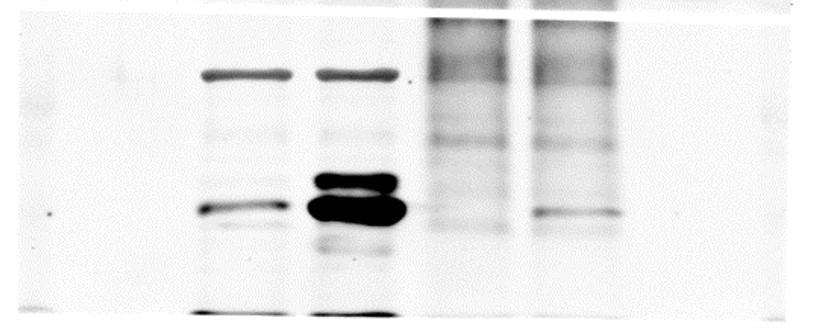

Supplement: Figure 5—figure supplement 1—source data 2. [file elife-85754-fig5-figsupp1-data2.zip › Figure 5-figure supplement 1-source data 2/Fig 5 S1B PIMT.jpg]

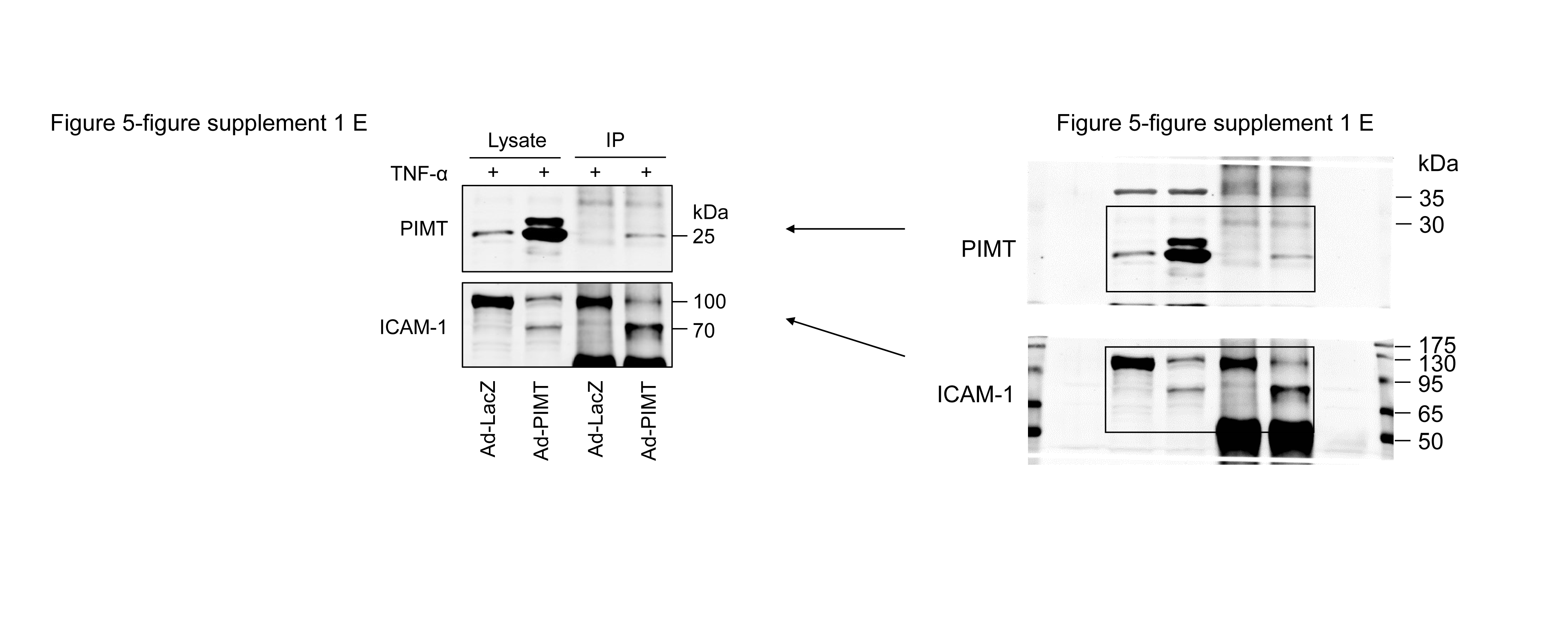

Supplement: Figure 5—figure supplement 1—source data 2. [file elife-85754-fig5-figsupp1-data2.zip › Figure 5-figure supplement 1-source data 2/Figure 5-figure supplement 1B.tif]

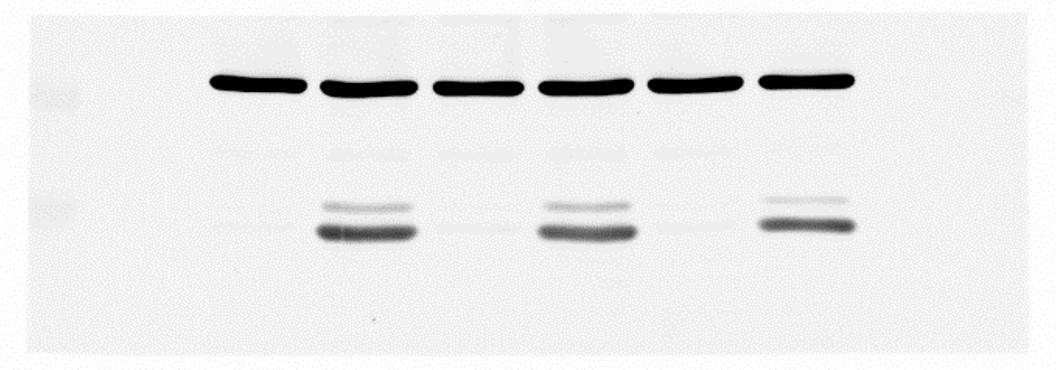

Supplement: Figure 6—source data 1. [file elife-85754-fig6-data1.zip › Figure 6- souce data 1/Fig 6B GAPDH.jpg]

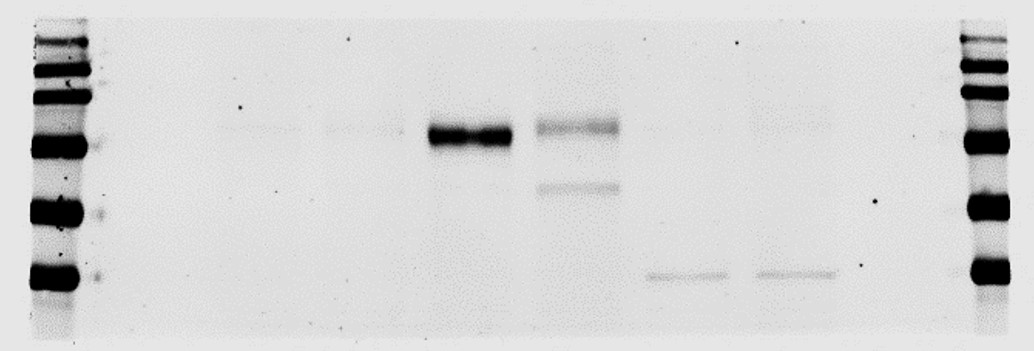

Supplement: Figure 6—source data 1. [file elife-85754-fig6-data1.zip › Figure 6- souce data 1/Fig 6B ICAM-1.jpg]

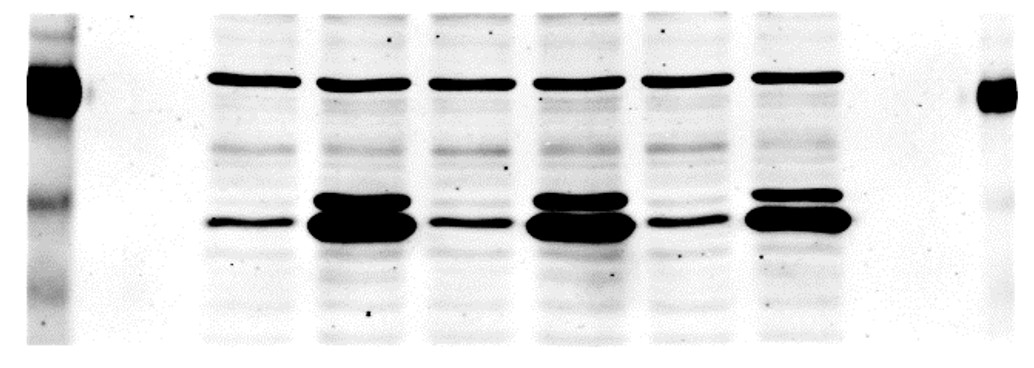

Supplement: Figure 6—source data 1. [file elife-85754-fig6-data1.zip › Figure 6- souce data 1/Fig 6B PIMT.jpg]

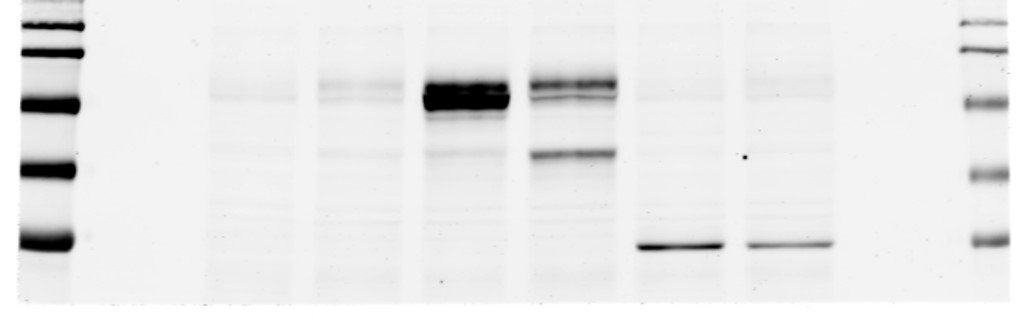

Supplement: Figure 6—source data 1. [file elife-85754-fig6-data1.zip › Figure 6- souce data 1/Fig 6B VCAM-1.jpg]

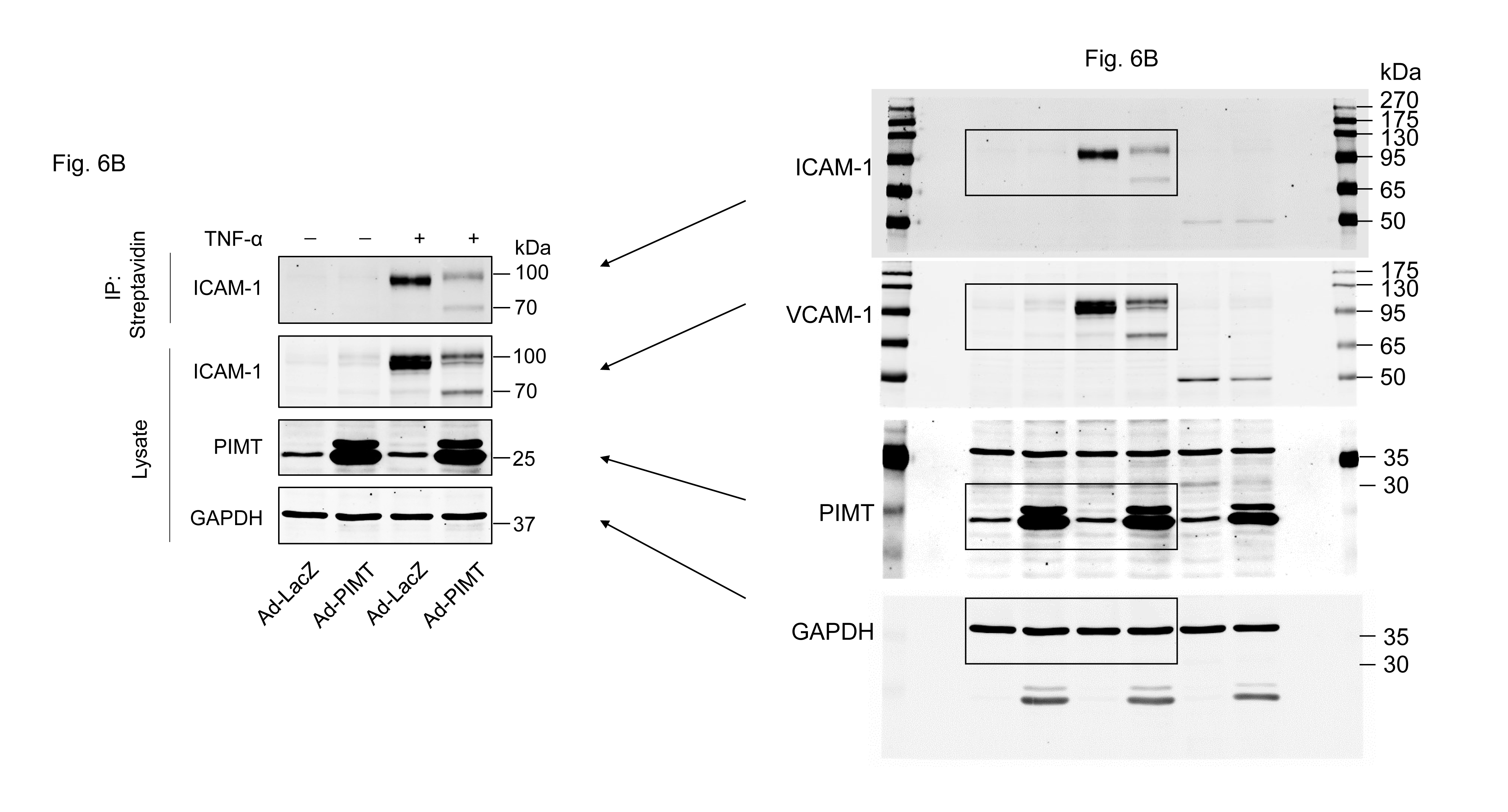

Supplement: Figure 6—source data 1. [file elife-85754-fig6-data1.zip › Figure 6- souce data 1/Figure 6B.tif]

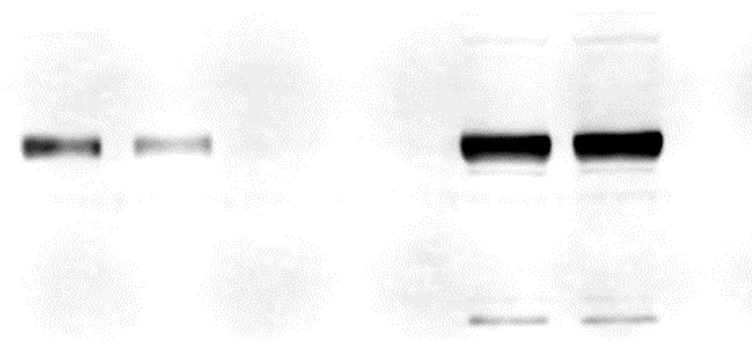

Supplement: Figure 6—source data 2. [file elife-85754-fig6-data2.zip › Figure 6- souce data 2/Fig 6C Ac-atubulin.jpg]

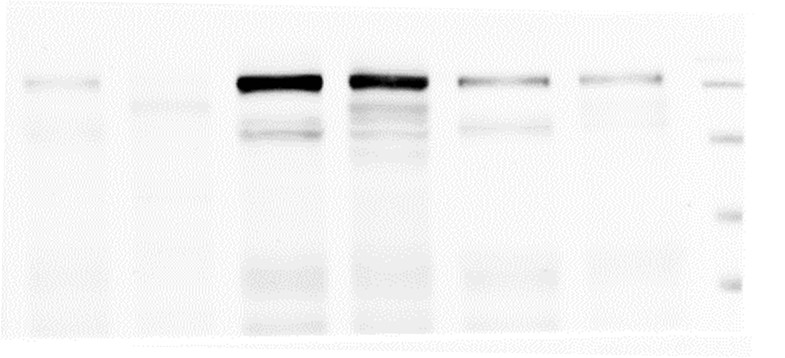

Supplement: Figure 6—source data 2. [file elife-85754-fig6-data2.zip › Figure 6- souce data 2/Fig 6C Cd31.jpg]

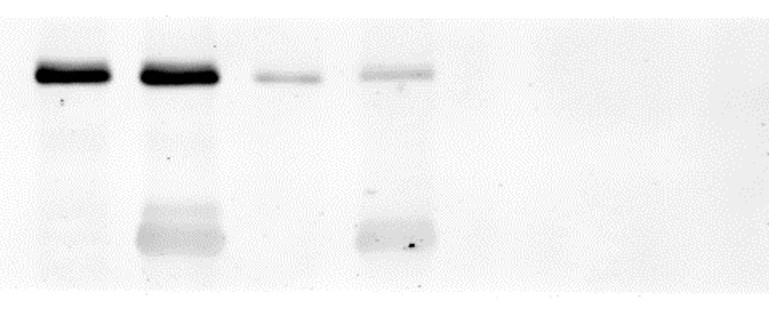

Supplement: Figure 6—source data 2. [file elife-85754-fig6-data2.zip › Figure 6- souce data 2/Fig 6C GAPDH.jpg]

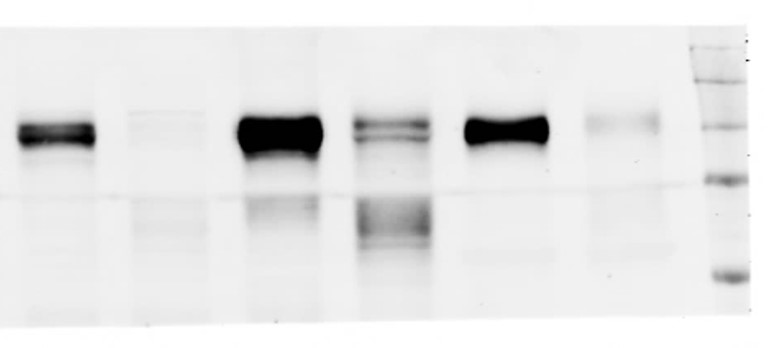

Supplement: Figure 6—source data 2. [file elife-85754-fig6-data2.zip › Figure 6- souce data 2/Fig 6C ICAM-1.jpg]

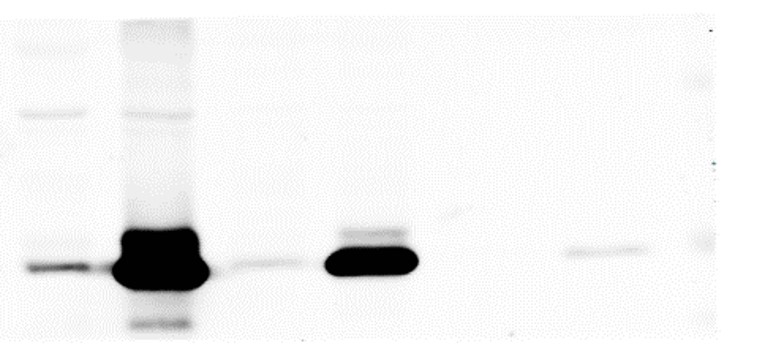

Supplement: Figure 6—source data 2. [file elife-85754-fig6-data2.zip › Figure 6- souce data 2/Fig 6C PIMT.jpg]

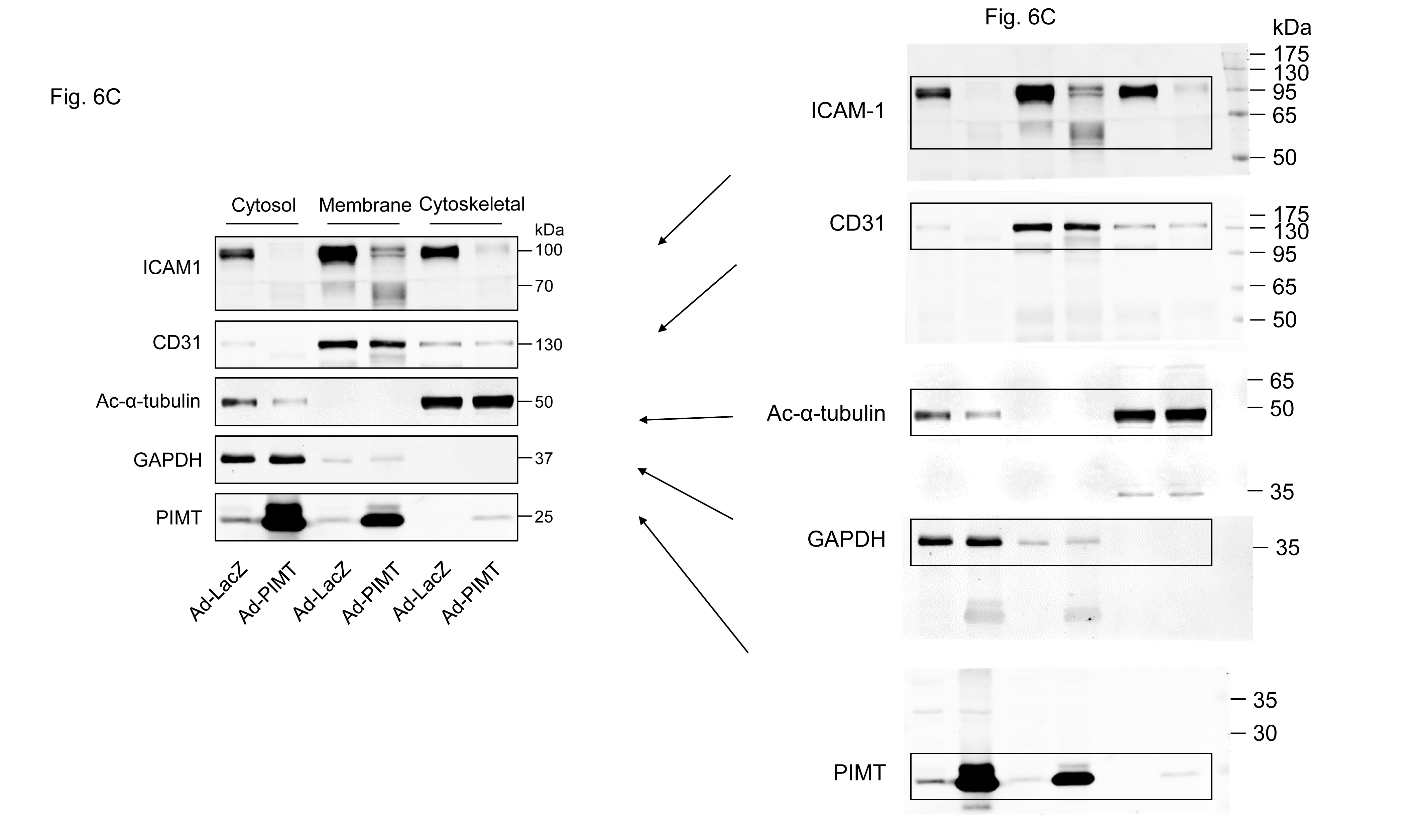

Supplement: Figure 6—source data 2. [file elife-85754-fig6-data2.zip › Figure 6- souce data 2/Figure 6C.tif]

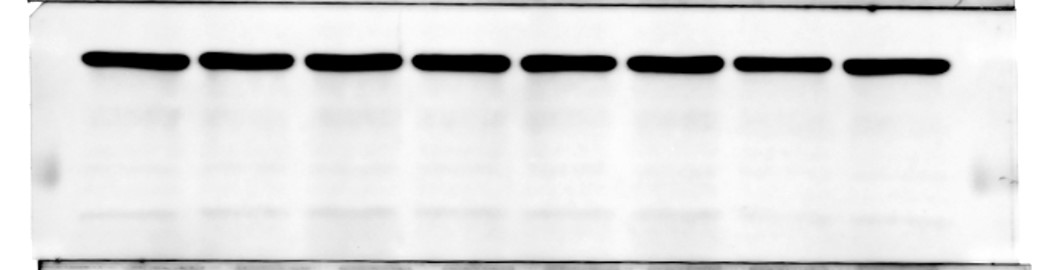

Supplement: Figure 6—source data 3. [file elife-85754-fig6-data3.zip › Figure 6- souce data 3/Fig 6D AdLacZ GAPDH.jpg]

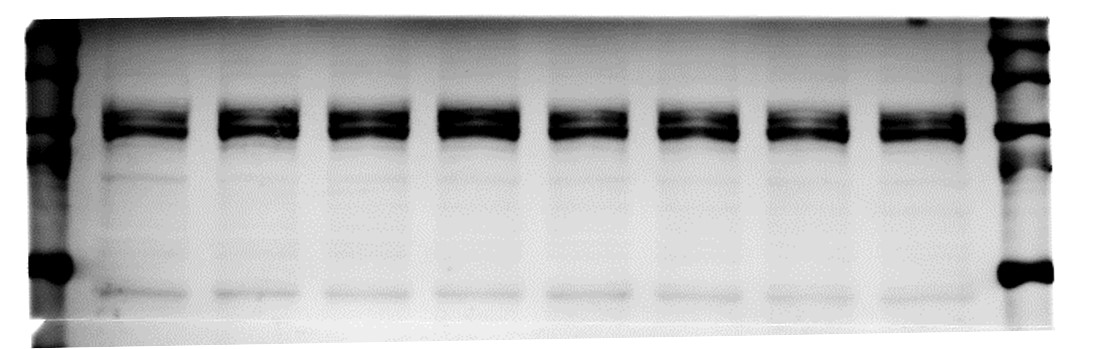

Supplement: Figure 6—source data 3. [file elife-85754-fig6-data3.zip › Figure 6- souce data 3/Fig 6D AdlacZ ICAM-1.jpg]

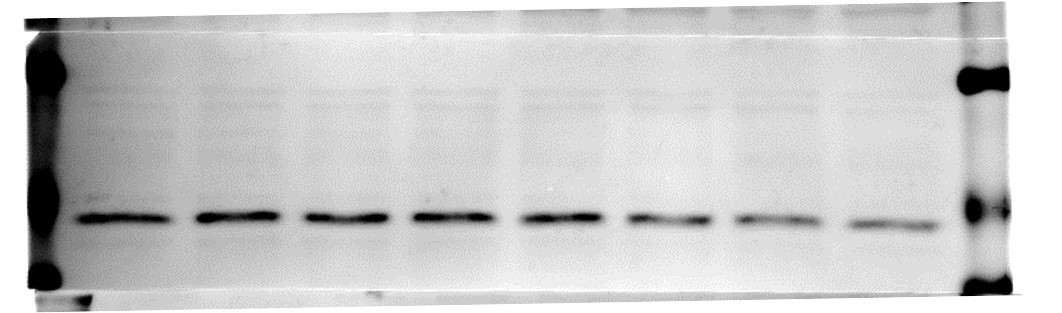

Supplement: Figure 6—source data 3. [file elife-85754-fig6-data3.zip › Figure 6- souce data 3/Fig 6D AdLacZ PIMT.jpg]

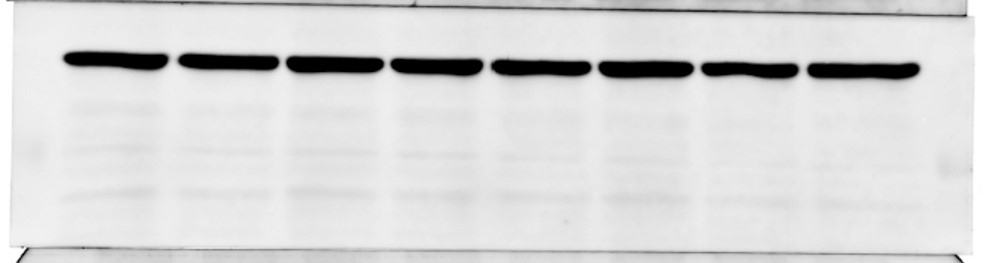

Supplement: Figure 6—source data 3. [file elife-85754-fig6-data3.zip › Figure 6- souce data 3/Fig 6D AdPIMT GAPDH.jpg]

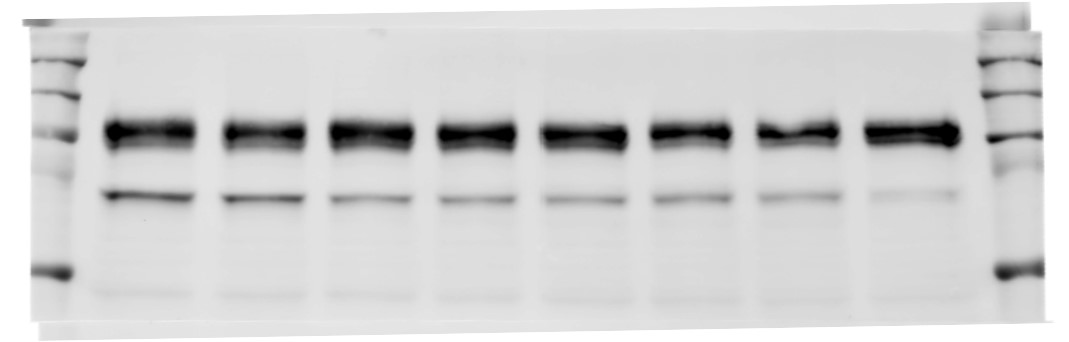

Supplement: Figure 6—source data 3. [file elife-85754-fig6-data3.zip › Figure 6- souce data 3/Fig 6D AdPIMT ICAM-1.jpg]

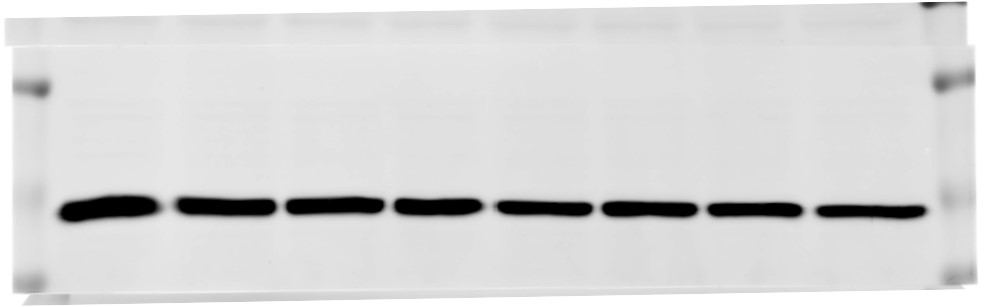

Supplement: Figure 6—source data 3. [file elife-85754-fig6-data3.zip › Figure 6- souce data 3/Fig 6D AdPIMT PIMT.jpg]

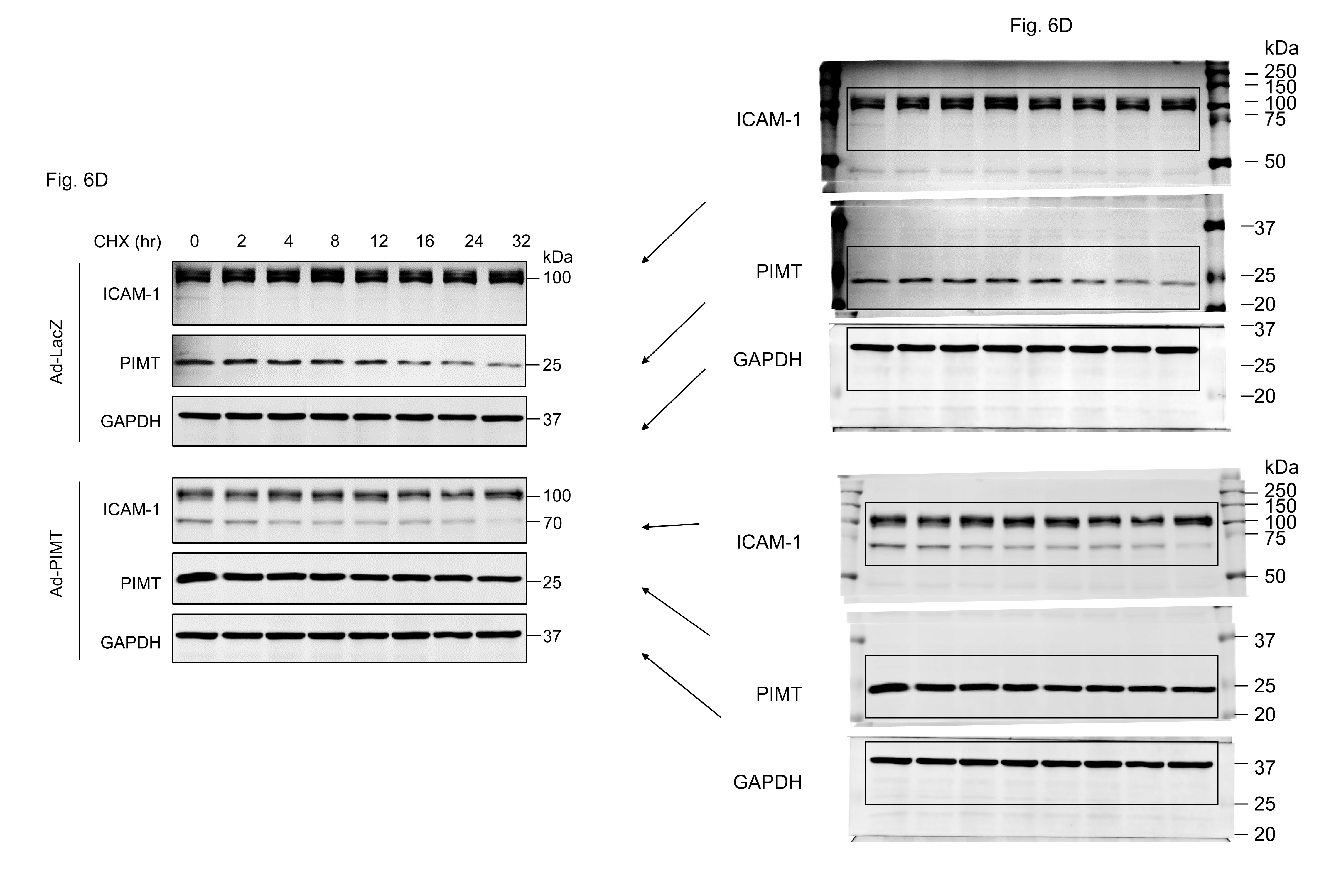

Supplement: Figure 6—source data 3. [file elife-85754-fig6-data3.zip › Figure 6- souce data 3/Figure 6D.tif]

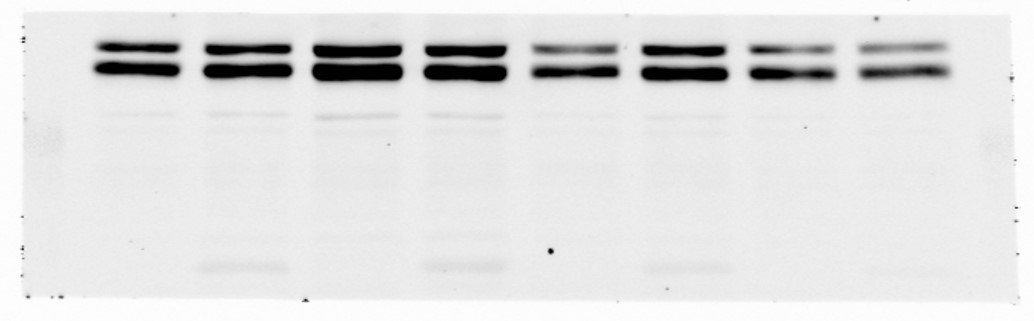

Supplement: Figure 6—source data 4. [file elife-85754-fig6-data4.zip › Figure 6- souce data 4/Fig 6F ERK.jpg]

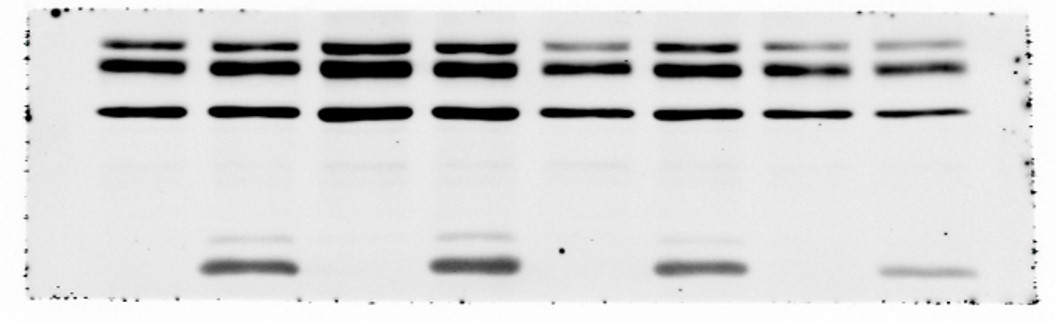

Supplement: Figure 6—source data 4. [file elife-85754-fig6-data4.zip › Figure 6- souce data 4/Fig 6F GAPDH.jpg]

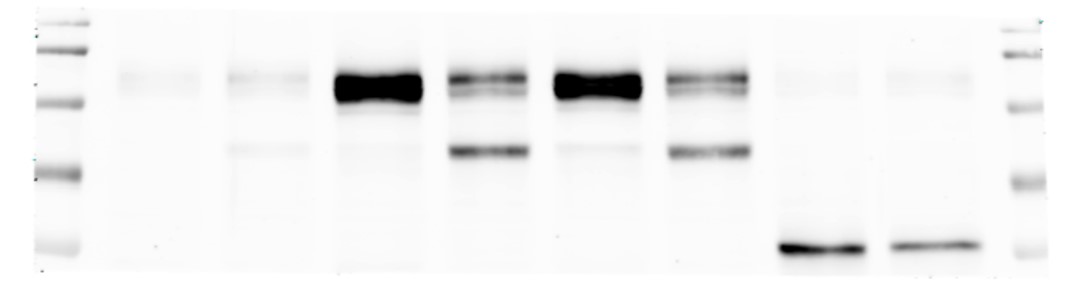

Supplement: Figure 6—source data 4. [file elife-85754-fig6-data4.zip › Figure 6- souce data 4/Fig 6F ICAM-1.jpg]

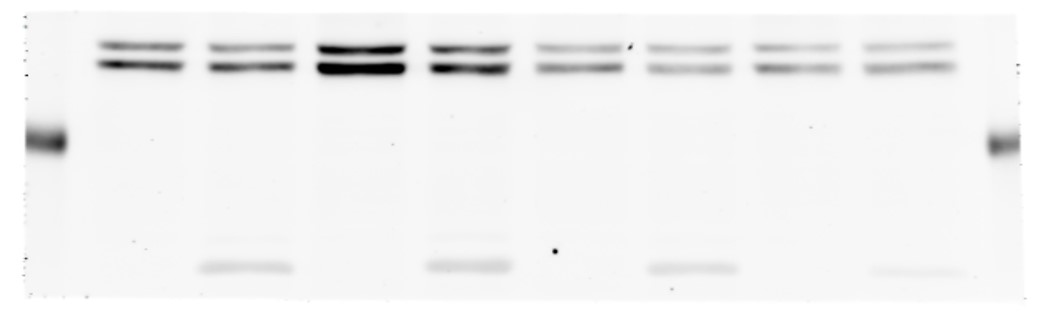

Supplement: Figure 6—source data 4. [file elife-85754-fig6-data4.zip › Figure 6- souce data 4/Fig 6F P-ERK.jpg]

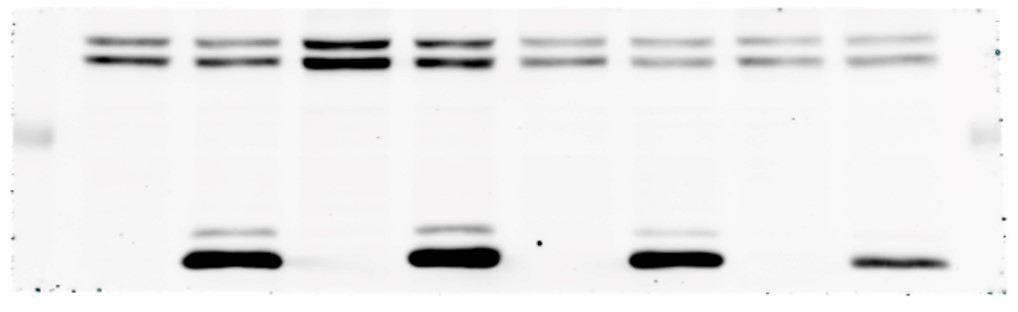

Supplement: Figure 6—source data 4. [file elife-85754-fig6-data4.zip › Figure 6- souce data 4/Fig 6F PIMT.jpg]

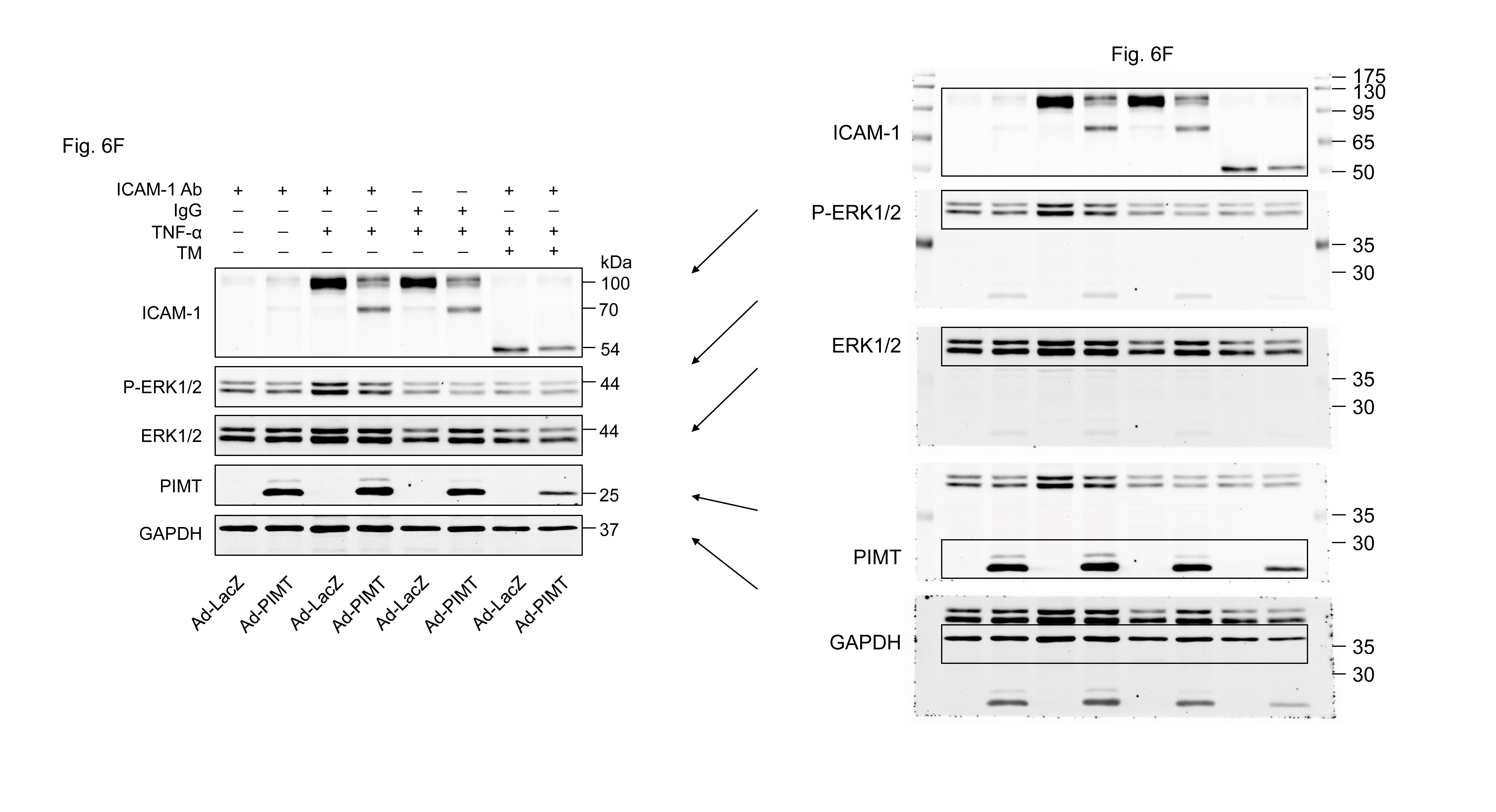

Supplement: Figure 6—source data 4. [file elife-85754-fig6-data4.zip › Figure 6- souce data 4/Figure 6F.tif]
